# Zero-shot prediction of mutation effects with multimodal deep representation learning guides protein engineering

**DOI:** 10.1038/s41422-024-00989-2

**Published:** 2024-07-05

**Authors:** Peng Cheng, Cong Mao, Jin Tang, Sen Yang, Yu Cheng, Wuke Wang, Qiuxi Gu, Wei Han, Hao Chen, Sihan Li, Yaofeng Chen, Jianglin Zhou, Wuju Li, Aimin Pan, Suwen Zhao, Xingxu Huang, Shiqiang Zhu, Jun Zhang, Wenjie Shu, Shengqi Wang

**Affiliations:** 1 Bioinformatics Center of AMMS, Beijing, China; 2 State Key Laboratory of Reproductive Medicine and Offspring Health, Women’s Hospital of Nanjing Medical University, Nanjing Maternity and Child Health Care Hospital, Nanjing Medical University, Nanjing, Jiangsu China; 3 Zhejiang Lab, Hangzhou, Zhejiang China; 4 iHuman Institute, ShanghaiTech University, Shanghai, China; 5 School of Life Science and Technology, ShanghaiTech University, Shanghai, China

**Keywords:** Bioinformatics, Protein folding

## Abstract

Mutations in amino acid sequences can provoke changes in protein function. Accurate and unsupervised prediction of mutation effects is critical in biotechnology and biomedicine, but remains a fundamental challenge. To resolve this challenge, here we present Protein Mutational Effect Predictor (ProMEP), a general and multiple sequence alignment-free method that enables zero-shot prediction of mutation effects. A multimodal deep representation learning model embedded in ProMEP was developed to comprehensively learn both sequence and structure contexts from ~160 million proteins. ProMEP achieves state-of-the-art performance in mutational effect prediction and accomplishes a tremendous improvement in speed, enabling efficient and intelligent protein engineering. Specifically, ProMEP accurately forecasts mutational consequences on the gene-editing enzymes TnpB and TadA, and successfully guides the development of high-performance gene-editing tools with their engineered variants. The gene-editing efficiency of a 5-site mutant of TnpB reaches up to 74.04% (vs 24.66% for the wild type); and the base editing tool developed on the basis of a TadA 15-site mutant (in addition to the A106V/D108N double mutation that renders deoxyadenosine deaminase activity to TadA) exhibits an A-to-G conversion frequency of up to 77.27% (vs 69.80% for ABE8e, a previous TadA-based adenine base editor) with significantly reduced bystander and off-target effects compared to ABE8e. ProMEP not only showcases superior performance in predicting mutational effects on proteins but also demonstrates a great capability to guide protein engineering. Therefore, ProMEP enables efficient exploration of the gigantic protein space and facilitates practical design of proteins, thereby advancing studies in biomedicine and synthetic biology.

## Introduction

Growing evidence from molecular evolution suggests that mutations in protein sequences are often associated with changes in protein function, which may lead to enzyme deficiencies,^1^ human diseases^2^ and viral escape.^3,4^ Deciphering the effects of mutations is thus important in many fields of biological sciences, particularly for the design of protein variants with enhanced or novel functions. Recent efforts have demonstrated that learning the effects of mutations aids in protein engineering and has the potential to overcome the challenges of directed evolution and rational protein design.^5–7^ By navigating the fitness landscape of the target protein and identifying a small set of advantageous mutations, mutation effect prediction could diminish the labor-intensive procedures stemming from multi-round random variation and screening,^8^ as well as reduce reliance on expert knowledge about protein structure and function during the rational design of proteins.^9,10^

Despite the importance, accurate modeling of mutation effects is a fundamental challenge due to the intricate interactions among numerous residues and the complex nature of mutational epistasis.^11,12^ Recent advances in high-throughput experimental technologies, such as deep mutational scanning (DMS),^13^ have led to significant improvements in the parallel assessment of mutations.^14,15^ However, due to the considerations of scale and costs, experimentally traversing the gigantic space of all possible protein sequences (${\sum }_{i=\,1}^{L}(({\prod }_{j=1}^{i}\left(L+1-j\right)\times 19)/i!)$ for a protein of length *L*, where *i* is the number of mutations, *i*! is the factorial of *i*) remains unfeasible. Substantial efforts have previously been made to predict mutational effects. Traditional modeling approaches aim to approximate mutational effects using one or a small subset of protein properties. For instance, variations in the physicochemical properties of amino acids may be used to estimate mutation tolerance.^16^ Alignment-based methods leverage evolutionary properties by identifying conserved regions or mutational patterns within multiple sequence alignments (MSAs).^17–19^ Stability predictors primarily operate on the principle of protein folding energy to assess functional changes resulting from mutations.^20^ Supervised learning methods learn the mapping from sequences or structures to a specific protein property using annotated datasets.^3,21,22^ While these methods are undoubtedly useful for predicting mutation effects, their performance is contingent on the depth of MSAs, the availability of labeled datasets, or the type of proteins.

Propelled by the swift advancement of natural language-processing techniques, sequence-based representation learning models or protein language models (e.g., Unirep,^7^ ESM^23^ and ProtTrans^24^) emerge as an unsupervised and MSA-free approach to predict mutation effects.^25,26^ Despite these promising developments, the accurate prediction of mutation effects remains a challenge. This is largely due to the lack of detailed structure context in these models, which is more evolutionarily conserved than sequences and includes crucial long-range contact information for protein functionality.^27^ The recently published AlphaMissense^28^ has demonstrated remarkable efficacy in predicting the pathogenicity of missense variants through the utilization of protein structure context. Nonetheless, its reliance on MSAs introduces a significant time burden in searching and processing MSAs.^29^

In this study, we introduce Protein Mutational Effect Predictor (ProMEP), a multimodal and MSA-free method that enables zero-shot prediction of mutation effects. To accurately predict the effects of mutation, we first develop a deep representation learning model as a base module in ProMEP, which integrates both sequence and structure context by tapping into ~160 million proteins in the AlphaFold protein structure database.^30^ ProMEP achieves state-of-the-art (SOTA) performance in predicting the effects of mutations. Owing to the MSA-free nature, ProMEP is 2–3 orders of magnitude faster than AlphaMissense, and demonstrated superior performance for proteins where MSAs are unavailable. Besides, ProMEP accurately predicted the mutational consequence on editing enzymes TnpB^31^ and TadA,^32,33^ and successfully guided the development of high-performance gene-editing tools based on their engineered variants. The gene-editing efficiency of the 5-site mutant TnpB increased to 74.04% at the *RNF2* site 1. For the 15-site mutant TadA, the corresponding base editing tool exhibited an A-to-G conversion frequency of up to 77.27% at the *HEK* site 7 A6 while significantly reducing bystander and off-target effects compared to ABE8e.^32^ Collectively, ProMEP not only demonstrates superior performance in predicting the mutational effects of proteins in a zero-shot manner, but also establishes intelligent strategies to engineer proteins with enhanced functionality and minimal experimental burden. ProMEP enables high-throughput and cost-effective exploration of the vast uncharted realms of protein space, as well as facilitates intelligent protein engineering and design.

## Results

### A multimodal deep representation learning model for proteins

To integrate both sequence and structure information of proteins, we developed a multimodal deep representation learning model (Supplementary information, Fig. S1a; Materials and methods) with ~659.3 million parameters. The model was trained on ~160 million AlphaFold2 structures by completing the missing elements from corrupted input using both sequence and structure information (Materials and methods). We utilized the protein point cloud as a novel representation of protein structures, allowing ProMEP to incorporate structure context at atomic resolution (Materials and methods; Supplementary information, Fig. S1b, c). Besides, we adopted a rotation- and translation-equivariant structure embedding module to capture this structure context, which is invariant to three-dimensional (3D) translations and rotations (Materials and methods; Supplementary information, Fig. S1d).

To evaluate the performance of our proposed model, a thorough assessment was conducted using 15 datasets containing protein annotations, including the Enzyme Commission (EC) number, gene ontology (GO) terms, and protein–protein interactions (PPIs) (Materials and methods). Leading deep representation learning methods that solely utilize sequence (e.g., UniRep^7^ and ESM^23^) or structure (e.g., GearNet^33^), and existing shallow multimodal methods (e.g., DeepFRI^34^) that integrate sequence and simplified structure information were used for comparison. Our proposed model demonstrates SOTA performance across all seven function annotation datasets and eight PPI prediction datasets (Supplementary information, Fig. S2 and Tables S1, S2). Through robustness tests conducted on 4 function annotation datasets and 3 PPI prediction datasets (Materials and methods), we observed that our multimodal representations can capture functional properties even in proteins with low sequence similarity or low structure similarity (Supplementary information, Fig. S3). Extensive generalization tests (Materials and methods) demonstrated that our multimodal representations facilitated one-shot function prediction (Supplementary information, Fig. S4a, b) and generalized well across species (Supplementary information, Fig. S4c).

Collectively, our proposed model illuminates a multimodal approach to learn both sequence and structure context from massive protein datasets. It summarizes arbitrary protein structures into semantically rich representations approximating protein functions and achieves superior and generalizable performance across comprehensive benchmarks.

### Zero-shot prediction of mutation effects on proteins based on multimodal representations

**Fig. 1:**
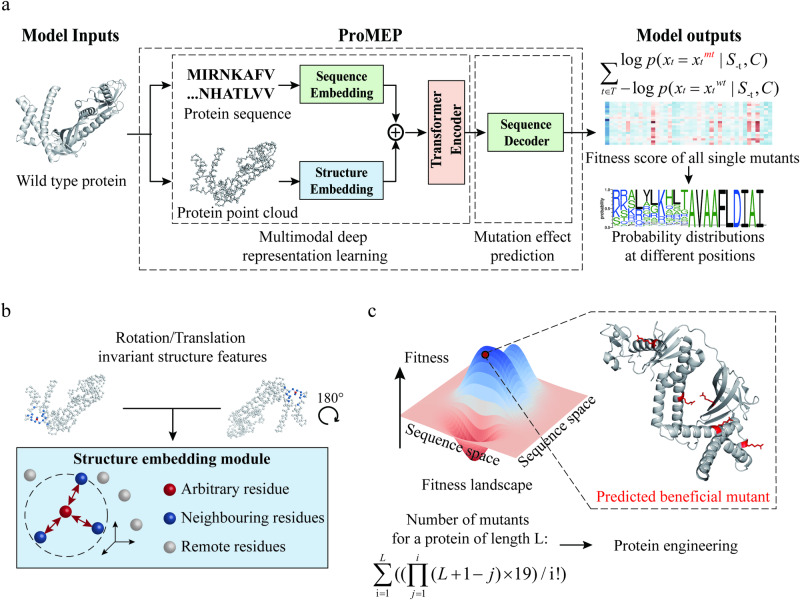
A multimodal mutation effect predictor for protein engineering tasks. **a** ProMEP combines the sequence context and the structure context of a protein to accurately predict mutation effects in a zero-shot manner. It takes an arbitrary WT protein as input and uses the pre-trained multimodal deep representation learning model to calculate semantic-rich representations for each amino acid of a protein. Specifically, for arbitrary mutations, ProMEP first extracts both sequence embeddings and structure embeddings from the WT protein. These embeddings are then aligned and fed into the pre-trained transformer encoder to generate protein representations at residual resolution. With the sequence decoder, fine-grained protein representations are eventually decomposed into the conditional probabilities on each amino acid under the contexts of both sequence and structure. Effects of an arbitrary mutation can be interpreted as the difference in predicted log-likelihood between the mutated sequence and the WT sequence. A customized protein point cloud is adopted to introduce protein structure context at atomic resolution. **b** 3D translations and rotations of the input protein structure will not affect the structure context of a protein. ProMEP applies a rotation- and translation-equivariant structure embedding module to guarantee such invariance. **c** ProMEP can be used to guide protein engineering without the requirements for labeled datasets or a holistic understanding of the protein structure and molecular function. It enables the user to recognize beneficial (multiple) mutants by efficiently traversing the protein fitness landscape.

Based on our multimodal deep representation learning model, we proposed ProMEP (Fig. 1a) to predict the mutation effects on proteins in a zero-shot manner. The log-ratio heuristic, which compares the probabilities of wild-type (WT) and mutated amino acids, has proven effective in estimating mutation effects.^18,25,26^ While previous methods calculate this score solely conditioning on sequence context, our multimodal architecture allows ProMEP to quantify the log-likelihood of protein variants with combinational sequence and structure contexts (Fig. 1b). By comparing probabilities of the WT sequence and the mutant sequence, ProMEP could accurately depict the protein fitness landscape and guide protein engineering by recognizing beneficial (multiple) mutants (Fig. 1c).

**Fig. 2:**
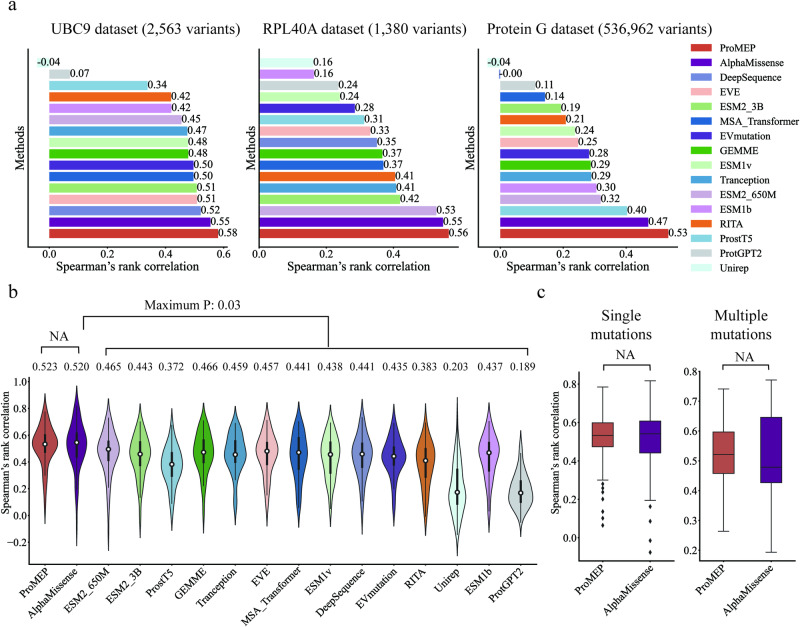
Performance of ProMEP in the prediction of mutation effects. **a** Spearman’s rank correlation between predicted mutation effects and experimental measurements across three representative DMS datasets (Materials and methods). Compared with the current SOTA methods, ProMEP achieves significantly better performance on all proteins (*P* value < 0.00001). **b** Spearman’s rank correlation of ProMEP on the ProteinGym benchmark, which contains 1.43 million variants covering 53 proteins derived from prokaryotes, human and other eukaryotes. ProMEP shows comparable performance with AlphaMissense and achieves superior performance than a comprehensive suite of baselines (*P* value < 0.05). **c** Performance comparison between ProMEP and AlphaMissense on single mutations (left) and multiple mutations (right) in the ProteinGym benchmark.

To benchmark whether ProMEP could predict mutation effects for proteins spanning diverse functions, we sourced three representative proteins for which experimental measurements of protein variant effects are available: the SUMO-conjugating enzyme UBC9 dataset,^35^ the RPL40A dataset^36^ and the immunoglobulin G-binding protein G dataset^12^ (Materials and methods). Spearman’s rank correlation between the model predictions and the experimental measurements was utilized as the standard metric to evaluate the performance of the model.^26,28,37^ Leading deep learning methods for mutation effect prediction were evaluated for comparison, including both MSA-based methods (e.g., AlphaMissense^28^ and EVE^37^) and MSA-free methods (e.g., ESM2_3B and ESM2_650M,^29^ ESM1v^25^ and Tranception^26^). ProstT5,^38^ a structure-enhanced protein language model was also evaluated as a baseline method. ProMEP shows the best correlation with experimental measurements compared to other methods on all three datasets (Fig. 2a). Especially for the protein G dataset that contains multiple mutations, ProMEP achieves a Spearman’s rank correlation of 0.53 compared with 0.47 for the next-best model, AlphaMissense.

To validate the generalization ability of ProMEP in predicting mutation effects, we assessed model predictions against the ProteinGym benchmark.^26^ 1.43 million variants from all 53 proteins derived from prokaryotes, human and other eukaryotes collected in the ProteinGym benchmark were included. These proteins are measured by different assays, range in length (72–2016 aa) and take part in diverse biological processes (e.g., response to antibiotic, transcription and catalysis). Despite this huge divergence, ProMEP achieves an average Spearman’s rank correlation of 0.523, on par with AlphaMissense (average Spearman’s rank correlation of 0.520, *P* value = 0.91; *t*-test, two-sided) and ESM2_3B (average Spearman’s rank correlation of 0.443, *P* value = 0.03; *t*-test, two-sided) (Fig. 2b). For datasets containing mutants with multiple mutation sites, ProMEP consistently demonstrates comparable performance to AlphaMissense (average Spearman’s rank correlation of 0.522 vs 0.518, *P* value = 0.95; *t*-test, two-sided) (Fig. 2c). Evaluations on protein structure resolution demonstrated that ProMEP achieves similar performance when structures predicted by AlphaFold2 or ESMFold^29^ are used (Supplementary information, Fig. S5). Besides, ProMEP also tolerates structural noise and achieves superior performance relative to GEMME,^39^ which is the third-best model during evaluation, even when 5-Å jitter is introduced in predicted structures.

Taken together, ProMEP demonstrates the ability to accurately interpret the underlying impact of mutations. The exceptional prediction efficiency and generalization ability of ProMEP imply its potent potential in predicting mutational effects in proteins without prior knowledge.

### Characterization of sequence and structure contexts captured by ProMEP

**Fig. 3:**
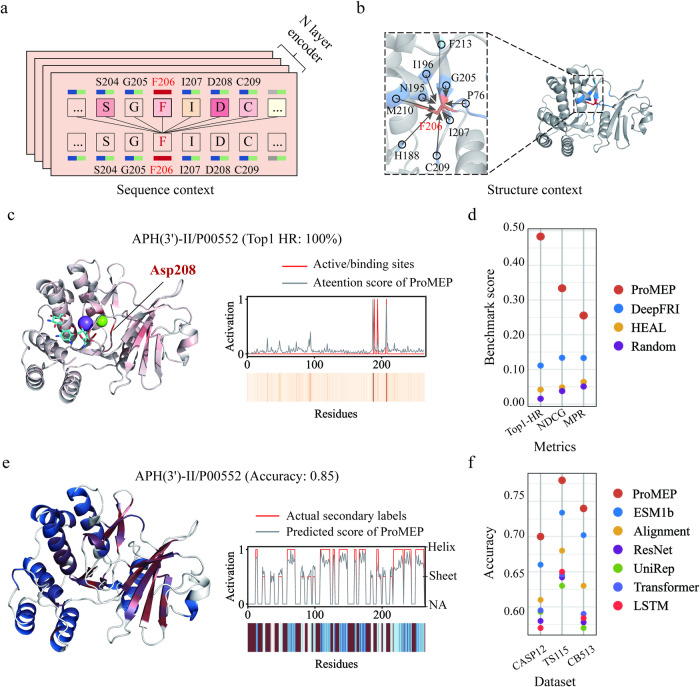
ProMEP captures both the sequence context and the structure context of a protein. **a**, **b** An example of the sequence context (**a**) and the structure context (**b**) of a protein during mutation effect prediction. For an arbitrary amino acid, we refer to the sequence context as those sequentially nearby amino acids with a maximum context size of 1024. We refer to the structure context as those spatially nearest neighbor amino acids with a maximum number of 30. **c** Visualization of interactions between sequential amino acids on the real structure of APH(3′)-II (UniProt accession ID: P00552). Left: more salient residues are highlighted in red in the presented structure. Right: the quantified interaction score of each position is presented in the functional site identification map. Actual functional sites (active/binding sites) are labeled as the red line. The interaction scores are labeled as the gray line. **d** Generalization tests of sequence context perception ability of ProMEP on a functional site identification benchmark (Materials and methods). We report the Top-1 HR, NDCG and MRR of each method. **e** Visualization of the secondary structure context on the real structure of APH(3′)-II. Left: residues with a higher probability corresponding to β-sheet are highlighted in red, and a higher probability corresponding to α-helix are highlighted in blue. Right: the quantified prediction score of each position is presented in the secondary structure heat map. Actual secondary labels are indicated as the red line. The predicted scores of ProMEP are labeled as the gray line. **f** Generalization tests of secondary structure context perception ability of ProMEP on an 8-class secondary structure classification benchmark. Other pre-trained protein language models (e.g., ESM1b and UniRep) and an alignment-based method (Alignment) are evaluated. We report the accuracy of each method on three test sets.

The multimodal architecture enables ProMEP to detect both the interaction between sequentially nearby amino acids (sequence context) (Fig. 3a) and the interaction between spatially nearby amino acids (structure context) (Fig. 3b). To interpret these contexts at different scales, we made an in-depth analysis of ProMEP.

First, we assessed the perception ability of the sequence context in ProMEP. Since ProMEP utilizes the attention mechanism to capture the sequence context, we quantified the attention score for all amino acids in a protein and analyzed whether this score is related to the functional sites (Materials and methods), which play a crucial role in molecular interactions and are vital for modeling the effects of mutations.^40,41^ For instance, the aminoglycoside 3′-phosphotransferase (APH(3′)-II) dataset within the ProteinGym benchmark encompasses the functional site annotations of the protein. We observed that ProMEP placed the most attention on Asp208, which serves as a Mg^2+^-binding site of APH(3′)-II (Fig. 3c), and achieved a top-1 hit ratio (Top-1 HR) of 100%. Similar phenomena were noted in other proteins in the ProteinGym benchmark (Supplementary information, Fig. S6a). We also constructed a functional site identification benchmark, which contains 1325 proteins randomly selected from Swiss-Prot, to examine the sequence context perception ability of ProMEP (Materials and methods). Although identifying functional sites in a protein can be challenging without expert knowledge of its structure and molecular function, we found that the amino acid receiving the most attention in ProMEP is likely to correspond to a functional site (with a 48.30% Top-1 HR within the Swiss-Prot dataset). Besides, ProMEP demonstrates superior performance than two structure-based functional site prediction baselines^34,42^ on all three metrics, including the normalized discounted cumulative gain (NDCG), and the mean reciprocal rank (MRR) (Fig. 3d).

Subsequently, we examined the ability of ProMEP to capture the local secondary structure context at each position within a protein. The accuracy of the mapping between the ProMEP representation of each amino acid and the corresponding actual secondary structure labels was used as our measurement (Materials and methods). For APH(3′)-II which also encompasses experimentally determined tertiary structure, ProMEP achieves an accuracy of 0.85 in capturing the actual secondary structure (Fig. 3e). To further validate its performance, we assessed ProMEP’s ability to classify secondary structures using standardized benchmarks, including CASP12, TS115 and CB513 (Materials and methods). In-silico comparisons were conducted with various baselines, which include pre-trained protein language models and alignment-based methods. ProMEP outperforms all baseline methods across three common datasets (Fig. 3f). For CB513, ProMEP achieves an accuracy of 0.74 compared with 0.70 for the next-best model, ESM1b. Furthermore, the evaluation on three additional benchmarks confirms its superior capability to accurately capture local structure context, including B-factor and solvent-accessible surface area (Supplementary information, Fig. S6b; Materials and methods).

Additionally, we assessed the perception capability of ProMEP in terms of the global protein folding context by employing a multi-class fold classification benchmark. This benchmark consists of 13,265 domains that were carefully selected from the Structural Classification of Proteins-extended (SCOPe) v2.07 database (Materials and methods). In comparison to previous structure-based models, which were trained based on either contact maps or protein graphs, the representations generated by ProMEP exhibit a remarkable ability to be accurately categorized into the correct fold classes (Supplementary information, Fig. S6c). More specifically, ProMEP demonstrates a substantial improvement in the classification of fold classes that are sparse in nature, such as multi-domain proteins (class e, F1 score of 0.92 of ProMEP vs 0.71 of the next-best model, GraSR) and small proteins (class g, F1 score of 0.92 of ProMEP vs 0.62 of the next-best model, GraSR).

Finally, the ablation study of ProMEP on three datasets demonstrates that both the sequence context learned by the sequence embedding module and the structure context learned by the structure embedding module markedly contribute to the improved performance (Supplementary information, Fig. S7). Collectively, these findings demonstrated that ProMEP is capable of capturing both the sequence context and the structure context of a protein. Furthermore, the quantitative and visually interpretable multimodal context provides significant insights for biologists to understand the molecular function of both annotated and unannotated proteins.

### Predicting mutation effects for proteins with low number of homologs using ProMEP

**Fig. 4:**
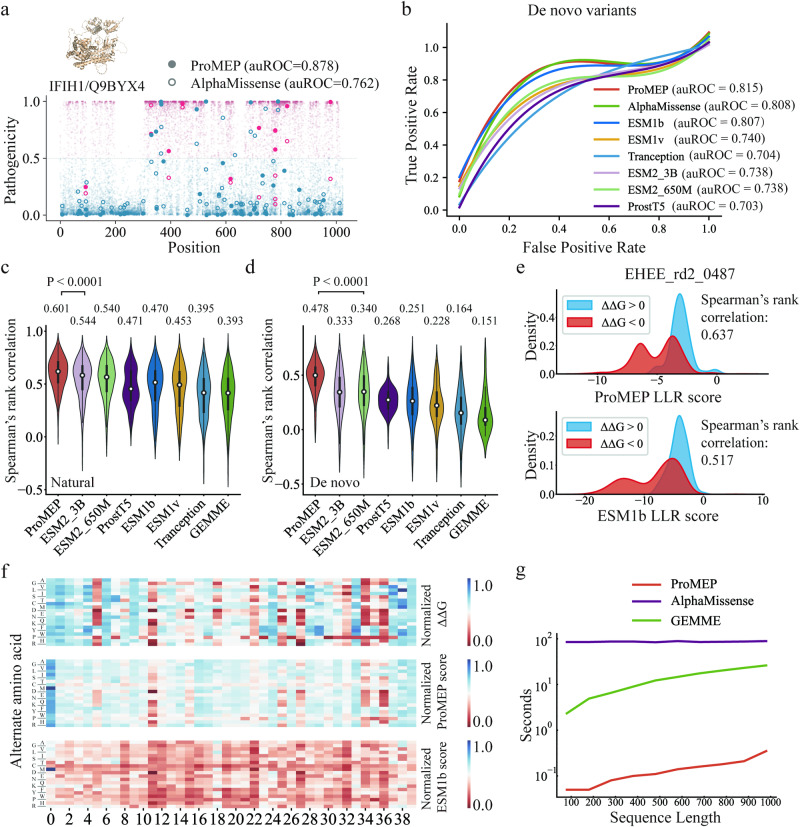
ProMEP is an MSA-free method that benefits less-studied or de novo designed proteins. **a** A comparison of prediction performance, assessed through the auROC metric, was conducted between ProMEP and AlphaMissense using example proteins selected from ClinVar. The protein name is presented in the format “[HUGO symbol]/[Uniprot accession ID]”. Missense variants, depicted as points, are graphically represented against ProMEP pathogenicity scores on the *y*-axis and amino acid positions on the *x*-axis. Variants predicted as likely pathogenic are denoted in red, while those predicted as likely benign are shown in blue. If a variant has a clinical label in ClinVar, it is portrayed as a brighter circle. Solid circles signify variants predicted by ProMEP, whereas hollow circles represent variants predicted by AlphaMissense. **b** The performance of ProMEP on the pathogenicity prediction benchmark, which is composed of de novo variants identified within both patients and healthy controls participating in the DDD cohort. **c**, **d** The performance of ProMEP on a stability benchmark. Other MSA-free methods (e.g., ESM2_3B, ProstT5 and Tranception) and an MSA-based method (GEMME) are evaluated as performance baselines. We report the Spearman’s rank correlation of each method on natural (**c**) and de novo protein (**d**) domains, respectively. **e** The distribution of mutation effect scores (log-likelihood ratio, LLR) of ProMEP and ESM1b across two sets of variants on a de novo designed protein domain (EHEE_rd2_0487). ΔΔG values are used to distinguish different classes of variants. **f** Heat maps of measured ΔΔG and predicted effects of amino acid substitutions on a de novo designed protein domain (EHEE_rd2_0487). **g** The speed of ProMEP vs AlphaMissense and GEMME in processing proteins with sequence lengths up to 1000 (Materials and methods).

While AlphaMissense and other MSA-based methods depend on MSA to predict the impacts of mutations, ProMEP is an MSA-free method that can be used to explore unseen protein space. To validate this ability of ProMEP, we first evaluated ProMEP on proteins with low number of homologous sequences derived from the pathogenicity prediction benchmark (Materials and methods). AlphaMissense was evaluated as a representative MSA-based method. Six leading MSA-free methods (i.e., ESM2_3B, ESM2_650M, ProstT5, ESM1b, ESM1v and Tranception) were also evaluated. We observed that, without fine-tuning, ProMEP can accurately classify pathogenic variants for proteins with < 100 high similarity sequences (Fig. 4a; Supplementary information, Fig. S8). For protein Q9BYX4 that is encoded by gene *IFIH1*, ProMEP achieves an area under the receiver operating characteristic curve (auROC) of 0.878 compared with 0.762 for AlphaMissense. For de novo variants from Deciphering Developmental Disorders (DDD) cohort,^43^ ProMEP also demonstrates comparable performance to AlphaMissense (Fig. 4b).

Furthermore, we sourced a stability benchmark that contains de novo designed proteins for evaluation.^15^ Specifically, it consists of 776,000 high-quality folding stability values for all single amino acid variants and selected double mutants from 331 natural and 148 de novo designed protein domains. Most of these de novo designed protein domains exhibit a maximum identity (max ID) of 60% with any publicly available natural protein in the non-redundant (NR) protein sequence database (Supplementary information, Fig. S9a). We predicted the structures of all 479 protein domains via ESMFold.^29^ Since AlphaMissense did not provide pre-trained model weights, it was not evaluated on this dataset. Instead, we used GEMME^39^ for performance comparison, which is also an MSA-based method and performs quite well in the ProteinGym benchmark. For each protein, we calculated the Spearman’s rank correlation between model predictions and the measured thermodynamic folding stability (ΔG) of missense variants. Notably, irrespective of whether the protein domains are natural or de novo designed, ProMEP demonstrates significantly superior performance compared to other methods (Fig. 4c, d). For natural protein domains, ProMEP outperforms ESM2_3B with an average Spearman’s rank correlation of 0.601 compared to 0.544 (*P* < 0.0001, *t*-test, two-sided). Specifically, in the case of de novo designed proteins, ProMEP achieves an average Spearman’s rank correlation of 0.478, a substantial improvement over the average Spearman’s rank correlation of 0.340 attained by ESM2_650M (*P* < 0.0001, *t*-test, two-sided), the next-best MSA-free model. GEMME could not predict the effects of mutations on the majority of de novo proteins (131/148) because of the deficiency of MSAs.

To further elucidate the functional implications of ProMEP predictions, we compared the model’s fitness score with changes in protein thermodynamic stability (ΔΔG) of missense variants on individual de novo designed proteins. Across all mutants of de novo designed proteins, the median ΔΔG is −0.62 kcal/mol (Supplementary information, Fig. S9b), indicating that a missense variant is typically more unstable than the WT. For example, on three de novo designed proteins that share a max ID of < 10%, the distribution of ProMEP fitness scores shows a substantial difference between stable (ΔΔG > 0) and unstable (ΔΔG < 0) variants (Fig. 4e; Supplementary information, Fig. S9c–e). We visualized the fitness score on EHEE_rd2_0487 (40 aa, max ID < 10%) and compared the normalized measurements of ΔΔG with the normalized prediction scores of both ProMEP and ESM1b (Fig. 4f). Notably, ProMEP exhibits a higher degree of concordance between its predictions and the empirically measured ΔΔG values compared to ESM1b, implying a closer association between ProMEP predictions and protein fitness.

In addition to predicting mutation effects for low-homology proteins, ProMEP enables more efficient predictions of mutation effects. While AlphaMissense and other MSA-based methods require expensive time to search and process MSAs, ProMEP avoids this bottleneck by learning sequence context and structure context from massive datasets. Analogous to the evaluation strategy used in the previous work,^29^ ProMEP makes a prediction on a protein with 1000 residues in 0.3 s, 296 times faster than AlphaMissense (Materials and methods; Fig. 4g). The speed advantage of ProMEP primarily comes from bypassing MSA processing and improvements in model architecture. Instead of utilizing multi-layer Evoformers for processing MSAs like AlphaMissense, ProMEP uses a multimodal architecture to process a single sequence. On shorter sequences, we observed a ~1700-fold improvement in the speed of model prediction.

Overall, these observations indicate that ProMEP could be beneficial for estimating mutation effects on proteins with a limited number of homologs, especially for de novo designed proteins where MSA-based approaches lack statistical power.

### ProMEP-guided engineering of RNA-directed nuclease TnpB

ProMEP exhibits a remarkable proficiency in precisely forecasting the consequences of mutations within proteins, indicating its applicability for advancing protein engineering endeavors aimed at amplifying specific functionalities. We experimentally validate this ability of ProMEP using the gene editing-related enzymes, which have elicited considerable interest due to their vast potential in applications ranging from therapeutic interventions for various diseases to agricultural breeding practices.^44–46^ We first focused on the transposase-related RNA-guided nuclease TnpB,^31,47^ whose relatively low editing activity in mammalian cells limits its wide applications.

**Fig. 5:**
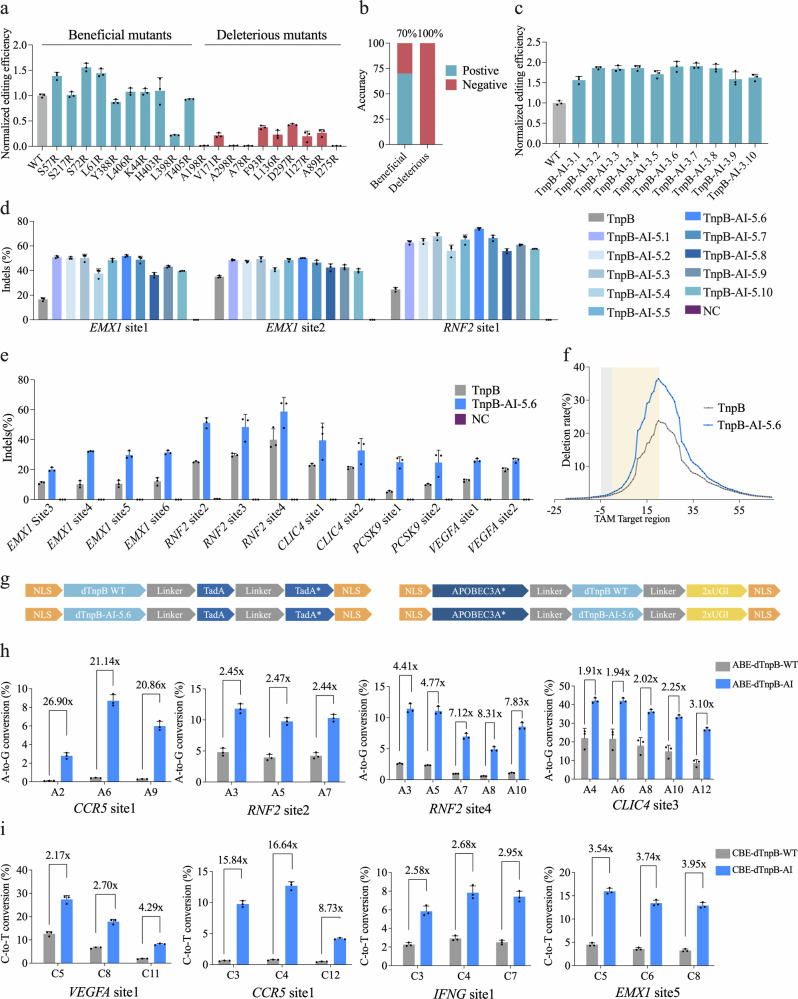
The engineering of TnpB guided by ProMEP enhances its editing efficiency in mammalian cells. **a** Editing efficiency of TnpB variants harboring either top 10 beneficial or deleterious mutations at *EMX1* site 1 is presented as the comparative fold change in InDel efficiency of these single-mutation variants relative to that of the WT TnpB. **b** Accuracy of the identification of both beneficial and detrimental single mutations in TnpB by ProMEP. **c** Editing efficiency of TnpB variants with triple mutations at *EMX1* site 1. Data are presented as the fold change in InDel efficiency for TnpB variants with triple mutations relative to that of WT TnpB InDel efficiency. **d** The editing efficiency of WT TnpB and its quintuple mutants was assessed at three endogenous genomic loci in HEK293T cells. NC negative control. **e** Comparison of the editing efficiencies of TnpB and TnpB-AI-5.6 at 13 genomic loci in human HEK293T cells. NC negative control. **f** Distribution of deletions generated by the WT TnpB and TnpB-AI-5.6 in HEK293T cells at the *AGBL1* site 1. The average efficiency of three biological replicates is symbolized by a single dot. **g** Schematic construct designs for ABEs derived from dTnpB and dTnpB-AI-5.6 with the WT TadA and ABE8e (TadA*), and miniature CBEs derived from dTnpB and dTnpB-AI-5.6 with the mutant APOBEC3A* (Y130F) and uracil glycosylase inhibitor (UGI). **h** A-to-G conversion efficiency in endogenous loci with ABEs derived from dTnpB and dTnpB-AI-5.6. **i** C-to-T conversion efficiency in endogenous loci with CBEs derived from dTnpB and dTnpB-AI-5.6. For **a**, **c**–**e**, **h**, **i**, data are means ± SD from three independent biological replicates.

Since structure-guided substitution of amino acid residues with arginine (R) has been shown to improve the editing activity of CRISPR-Cas proteins in mammalian cells,^48–52^ we applied ProMEP to predict the fitness score of all X-to-R mutants (e.g., S72R) of TnpB. Thus, the top 10 X-to-R mutants with the highest (beneficial) fitness scores or the bottom 10 X-to-R mutants with the lowest (deleterious) fitness scores were selected for experimental validation. The results showed that 7 out of the top 10 beneficial mutants exhibit increased activity of TnpB, indicating an accuracy rate of 70%, while all of the top 10 deleterious mutants show decreased activity, indicating an accuracy rate of 100% (Fig. 5a, b). Among 10 beneficial mutants identified by ProMEP, S72R exhibits the highest editing efficiency and results in ~1.56-fold activity improvement relative to the WT (Fig. 5a). We also analyzed the probability distributions of all amino acid types at different positions (Supplementary information, Fig. S10 and Data S1). ProMEP exhibits a high degree of confidence in predicting top-ranked beneficial mutations (e.g., S57R and S217R) as well as all deleterious mutations. For deleterious mutations (e.g., A198R and V171R), the WT amino acids predominate the probability distribution.

Furthermore, we used ProMEP to predict the effects of multi-site mutations. All triple X-to-R mutants of TnpB (8,510,740 mutants) were analyzed. All of the top 10 beneficial mutants exhibit at least 1.5-fold improvement in editing efficiency relative to the WT (*P* value < 0.05) (Fig. 5c). Then, we applied ProMEP to predict the editing efficiency of all quintuple X-to-R mutants (Materials and methods). The top 10 mutants were selected and named TnpB-AI-5.n(1–10). TnpB-AI-5.n(1–10) all demonstrate robust activity at three endogenous sites in HEK293T cells (Fig. 5d). Notably, TnpB-AI-5.6 (TnpB-D191A/S72R/K84R/E168R/K251R/V374R) shows a significantly increased editing efficiency at the *EMX1* site 1, from 16.64% in the WT to 51.78%. Concurrently, TnpB-AI-5.6 shows an enhanced gene-editing efficiency at *RNF2* site 1, reaching up to 74.04%, in contrast to 24.66% observed in the WT (Fig. 5d). Based on prior structural data analysis,^53,54^ the amino acid mutation sites of TnpB-AI-5.6 are located within the REC, WED and RuvC domains (Supplementary information, Fig. S11a). To further evaluate the characteristics of TnpB-AI-5.6, we chose a panel of 13 target loci from five genes and compared the editing activities between TnpB-AI-5.6 and TnpB-WT. The editing efficiencies of TnpB-AI-5.6 at these 13 loci are 1.31–4.73 times higher than those of TnpB-WT (Fig. 5e). Meanwhile, analysis of insertion and deletion (InDel) patterns reveals that TnpB-AI-5.6 induces larger deletions compared to the WT (Fig. 5f; Supplementary information, Fig. S11b). The analysis of off-target effects demonstrated that the enhanced enzymatic activity of TnpB-AI-5.6 concurrently results in a degree of non-specific cleavage (Supplementary information, Fig. S11c).

To determine whether the enhanced activity of TnpB-AI-5.6 could enable efficient base editing, we constructed nuclease-deactivated TnpBs by introducing the D191A mutation (dTnpB-WT representing TnpB-D191A and dTnpB-AI-5.6 representing TnpB-D191A/S72R/K84R/E168R/K251R/V374R). Futhermore, we constructed adenine base editor (ABE) and cytosine base editor (CBE) based on dTnpB-WT and dTnpB-AI-5.6, respectively (Fig. 5g). Testing at four endogenous sites in HEK293T cells, we found that ABE-dTnpB-AI exhibits the highest A-to-G conversion efficiency of 42.07% at the *CLIC4* site 3 A6, outperforming ABE-dTnpB-WT’s efficiency of 21.61% (Fig. 5h). Across all editing sites, ABE-dTnpB-AI achieves efficiency improvements ranging from 1.91- to 26.9-fold compared to ABE-dTnpB-WT (Fig. 5h). Consistently, CBE-dTnpB-AI achieves efficiency improvements ranging from 2.17- to 16.64-fold compared to CBE-dTnpB-WT at four target sites (Fig. 5i). Together, our results indicate that ProMEP can efficiently forecast the mutational effect of TnpB, and can further guide the engineering of TnpB to generate a versatile genome editor.

### ProMEP-guided engineering of adenosine deaminase TadA

**Fig. 6:**
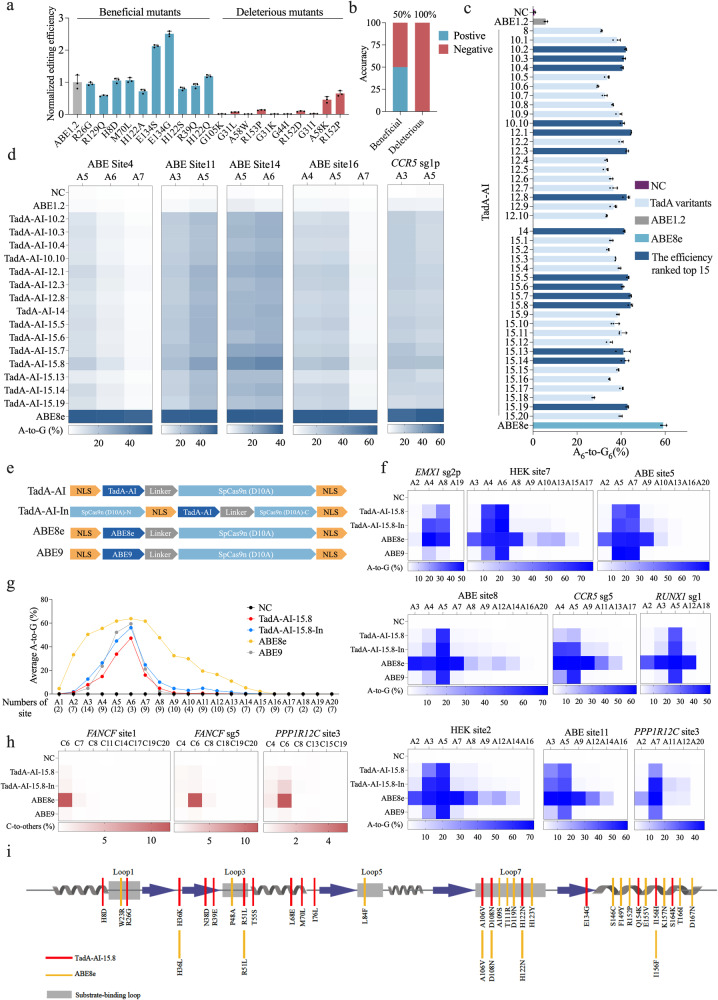
ProMEP identifies beneficial mutations from the gigantic sequence space in the engineering of TadA. **a** Editing efficiency of TadA variants harboring top 10 single beneficial mutations or deleterious mutations at an endogenous genomic locus (*PD1* sg4). Data shown are the comparative fold change in A-to-G conversion efficiency of TadA single mutants relative to ABE1.2. **b** Accuracy of the identification of both beneficial and deleterious single mutations in TadA by ProMEP. **c** Base editing efficiency of ABE1.2, ABE8e and TadA multi-site mutants at *PD1* sg4 in HEK293T cells. NC negative control. **d** Base editing efficiency of ABE1.2, ABE8e and TadA multi-site mutants at five endogenous genomic loci in HEK293T cells. **e** The architecture of TadA-AI-15.8, TadA-AI-15.8-In, ABE8e and ABE9. **f** The A-to-G conversion efficiency of TadA-AI-15.8, TadA-AI-15.8-In, ABE8e and ABE9 was examined at nine endogenous genomic loci in HEK293T cells. **g** Average A-to-G conversion efficiency of TadA-AI-15.8, TadA-AI-15.8-In, ABE8e and ABE9 at the 23 target sites. Data are means from three independent experiments. **h** The C-to-T/G/A conversion efficiency of TadA-AI-15.8, TadA-AI-15.8-In, ABE8e and ABE9 is examined at three endogenous genomic loci in HEK293T cells. NC negative control. **i** Secondary structure elements of the TadA enzyme are shown. The locations of the substrate-binding loops are indicated in gray, and the mutations in TadA-AI-15.8 and ABE8e are highlighted in red and yellow, respectively. In **d**, **f**, **h**, the heat map represents the average editing percentage derived from three independent experiments. For **a**, **c**, data are means ± SD from three independent biological replicates.

To examine the applicability of ProMEP in the engineering of other enzymes or target proteins, we focused on the tRNA adenosine deaminase TadA,^55,56^ which acquired a new function as a deoxyadenosine deaminase^57^ with the double mutation (A106V, D108N). Fusing this mutant with Cas9 nickase (D10A) creates an adenine base editor (ABE1.2) with limited A-to-G editing ability. To develop a precise and efficient ABE, we re-evolved ABE1.2 through ProMEP. We used ProMEP to compute the fitness scores for all 3173 single mutants of ABE1.2, and selected the top 10 beneficial mutants for validation. Five of the ten beneficial mutants exhibit average editing efficiencies greater than ABE1.2, indicating an accuracy rate of 50%, while the editing efficiencies for all top 10 deleterious mutants are lower than ABE1.2, indicating an accuracy rate of 100% (Fig. 6a, b). Especially, the E134S and E134G variants demonstrate significant improvements in editing efficiency of 2.12-fold and 2.50-fold compared to ABE1.2 (*P* value < 0.05) (Fig. 6a), respectively. The analysis of probability distributions of all amino acid types at different positions (Supplementary information, Fig. S12 and Data S2) shows high confidence of ProMEP in predicting beneficial and deleterious mutants. The WT amino acids of deleterious mutations (e.g., G105K and G31L) predominate the probability distribution, resembling the case of TnpB.

With the success in predicting activities of high-order mutants of TnpB, we directly utilized ProMEP to construct 41 TadA high-order mutants with at least ten mutations (Supplementary information, Fig. S13a). To differentiate these variants, we named them TadA-AI-n.x, where n represents the number of mutations contained within each variant on the basis of ABE1.2 and x numbers the variants harboring the same number of mutations (e.g., TadA-AI-10.2 indicates the 2nd TadA variant comprising ten mutations in addition to A106V and D108N). We assessed the activity of these new ABE variants in HEK293T cells and found that among all the evaluated TadA multi-site mutants, 18 of them exhibit an editing efficiency exceeding 40% at the *PD1* sg4 A6 position (Fig. 6c). Subsequently, the top 15 ABE variants were further validated at five additional endogenous sites in HEK293T cells. The result shows that the editing efficiency of TadA-AI-15.8 consistently surpassed those of other evaluated multi-site mutants at these five sites (Fig. 6d).

Next, we used TadA-AI-15.8 to construct an ABE using designs similar to ABE8e and ABE9.^9^ Also, we inserted TadA-AI-15.8 between nCas9 P1249 and E1250 (TadA-AI-15.8-In) (Fig. 6e).^58,59^ TadA-AI-15.8, TadA-AI-15.8-In, ABE8e and ABE9 were compared in parallel for their A-to-G editing efficiency at nine endogenous sites (Fig. 6f). Further improvement of editing efficiency was observed for TadA-AI-15.8-In compared to TadA-AI-15.8 (47.54% vs 16.31% on *CCR5* sg5 A5 and 77.27% vs 72.62% on *HEK* site 7 A6), which is consistent with prior studies showing that the embedding of TadA into nCas9 enhances the editing capabilities of ABEs.^58,59^ Upon integrating the average A-to-G conversion frequency across 23 endogenous sites, TadA-AI-15.8-In exhibits high editing activity at positions A5 and A6 of the editing window, with bystander editing effects close to those of ABE9 (44.88% vs 52.19% on A5 and 56.12% vs 59.46% on A6) (Fig. 6g). Next, we evaluated the cytosine deaminase activity of these ABEs. At three target sites, a significant decrease of cytosine conversion activity was observed for TadA-AI-15.8 and TadA-AI-15.8-In compared with ABE8e; the average editing efficiencies of TadA-AI-15.8, TadA-AI-15.8-In and ABE8e are 0.53%, 1.00% and 9.66% on position 6, respectively (Fig. 6h). The off-target effect analysis demonstrated that TadA-AI-15.8 and TadA-AI-15.8-In induced lower Cas9-dependent and Cas9-independent off-target effects than ABE8e (Supplementary information, Fig. S13b, c). Furthermore, RNA off-target analysis of TadA-AI-15.8 and TadA-AI-15.8-In shows 1.98-fold and 2.04-fold reduction compared to ABE8e (Supplementary information, Fig. S13d). Together, the ProMEP-evolved TadA variant TadA-AI-15.8 exhibits comparable editing activity and cytosine deaminase activity to those of ABE9 (Fig. 6g, h). Meanwhile, TadA-AI-15.8, with only four overlapped mutation sites with ABE8e on the basis of ABE1.2 (Fig. 6i; Supplementary information, Fig. S13e), demonstrates significantly diminished bystander and DNA/RNA off-target effects compared to ABE8e. In summary, our results demonstrated that ProMEP can accurately predict the mutational effects of TadA, and further guide the engineering of TadA with tens of mutations.

## Discussion

For efforts ranging from the design of new functional proteins, to the quantification of pathogenicity for less-studied protein variants, to the evolutionary prediction of new viruses, accurate and unsupervised prediction of mutation effects is critical to a wide range of applications. In this study, we present ProMEP for zero-shot prediction of mutation effects on proteins and demonstrate how ProMEP can be used to guide protein engineering (Fig. 1). A key aspect of this work distinguishing ProMEP from existing methods is its underlying multimodal deep representation model. Current leading approaches, such as DeepSequence,^18^ MSA-Transformer^60^ and Tranception,^26^ largely operate on sequence information of proteins for mutation effect prediction. In comparison, we hypothesize that leveraging structure context will improve the accuracy of mutation effect prediction since protein functions are largely encoded in its tertiary structures. To this end, we first develop a multimodal deep learning model that systematically learns both sequence context and structure context from ~160 million proteins with reliable structures (Supplementary information, Figs. S1–S4). We address the challenges of learning structure context at atomic accuracy from hundreds of millions of protein structures via our proposed protein point cloud and the multimodal architecture. By modeling the protein fitness landscape under the contexts of both sequence and structure, ProMEP significantly outperforms traditional sequence-based methods in predicting mutation effects (Figs. 2, 3). Specifically, for low-homology proteins or de novo designed proteins, ProMEP shows consistently superior performance (Fig. 4).

ProMEP uses protein structures predicted by ESMFold^29^ or AlphaFold2^61^ to predict mutation effects. This is a major advantage that enables ProMEP to predict mutation effects for any protein as long as its amino acid sequence is available for structure prediction. Since experimentally determined 3D structures are not available for most proteins,^62^ this is critical for the application of ProMEP. The results show that, compared with using experimentally determined 3D structures, ProMEP still achieves competitive performance in modeling mutation effects based on predicted structures, especially those predicted by AlphaFold2 (Supplementary information, Fig. S5). Moreover, robustness tests demonstrate that ProMEP could tolerate 5-Å jitter in predicted structures and still outperforms the current leading methods. On the one hand, these results demonstrate that incorporating structure information is important to accurately decode mutational effects. On the other hand, as structure prediction methods keep evolving, combining more accurate predicted structures could further improve the performance of ProMEP. In addition, without necessitating the structures of all protein variants, ProMEP uses a single WT protein structure for mutation effect prediction. This strategy allows ProMEP to introduce structure context selected through evolution, and explore gigantic protein space without sacrificing computational efficiency.

Benefiting from the increasing availability of protein structures predicted by AlphaFold2 and other advanced computational methods, the integration of protein structures into protein language models has emerged as a pressing question within the community. However, only limited efforts have been made.^38,63–66^ For instance, ProstT5^38^ and SaProt^63^ utilize Foldseek^67^ to represent protein structures as token sequences, subsequently employing these sequences as training data to fine-tune or retrain existing protein language models. While these methodologies open new avenues for the development of structure-enhanced protein language models, they have not yet been extensively employed for learning structural context on a large scale, nor have they been rigorously evaluated in the context of real-world protein engineering challenges. In comparison to these approaches, this paper introduces a novel and multimodal approach for predicting mutation effects. Through advancements in model architecture, the scale of training data, training objectives and downstream evaluations, our proposed method attains SOTA performance across a series of benchmarks and facilitates practical protein engineering tasks.

Both ProMEP and the recently published AlphaMissense aim to utilize structure context for mutation effect prediction, but they apply diverse strategies to achieve this goal. AlphaMissense is built on the AlphaFold methodology, which allows AlphaMissense to directly extract structure context from protein sequences and predict the effect of mutation. While this is a major advantage in AlphaMissense, it also leads to requirements for MSAs. In contrast, ProMEP is an MSA-free method that learns multimodal context from millions of proteins and then uses the learned knowledge to extract structure context from predicted structures. Our evaluation demonstrates that ProMEP achieves comparable performance with AlphaMissense on multiple benchmarks. As MSA-free structure prediction methods (e.g., ESMFold^29^ and RGN2^68^) are emerging, the MSA-free approach of ProMEP has two notable advantages. First, it allows ProMEP to predict mutation effects for proteins where MSAs are unavailable, such as proteins with low number of homologs or de novo designed proteins (Fig. 4a–f). Second, ProMEP inference is 2–3 orders of magnitude faster than AlphaMissense because of architecture improvement and no need to process the MSA branch (Fig. 4g). In summary, compared with AlphaMissense, ProMEP provides a fast and accurate method for mutation effect prediction, enabling the exploration of the gigantic protein fitness landscape in practical timescales.

Traditional directed evolution involves the iterative process of randomly constructing different mutants and screening for improved variants, followed by experimental quantification or qualitative screening of individual variants to identify the best ones. However, this process entails labor-intensive experiments and is often constrained by the throughput of screening and selection methods. Natural language-processing techniques are evolving rapidly. The previous research endeavors have sought to explore the application of protein language models in protein engineering tasks.^69,70^ However, these endeavors have predominantly focused on either developing new protein language models without assessing their applicability to practical protein engineering tasks or employing existing models without further innovation. In contrast, this study has advanced in both aspects. ProMEP can accurately predict the consequences of multi-site mutations without any preliminary samples, thereby manifesting remarkable predictive accuracy. This heralds a transformative paradigm in protein engineering, obviating the need for annotated datasets or an exhaustive grasp of protein structural and functional intricacies, and enabling expeditious protein evolution in a computational milieu. Utilizing ProMEP-guided protein engineering, we develop the small yet highly active TnpB quintuple mutants, and further derive base editors based on these variants, which comply with the packaging volume constraints of adeno-associated virus. Concurrently, the 15-site mutation engineering of the deaminase TadA guided by ProMEP further demonstrates the generalization performance of our model, providing advantageous tools for gene editing and therapy. Recent exploratory efforts in metagenomics have yielded a significant discovery of Cas proteins and deaminases,^71,72^ each exhibiting unique properties, providing ProMEP with an abundant repository of initial templates. As a straightforward and cost-effective tool for protein engineering, ProMEP offers an intelligent strategy to enhance protein functions, facilitating swift and efficient engineering modifications to generate novel proteins, and exhibits outstanding ability in the design of protein variants with multiple-site mutations.

Currently, ProMEP can quantify the effect of multiple amino-acid substitutions in arbitrary protein sequences, but cannot handle InDels, which could also cause functional changes and affect organismal adaptations.^73,74^ Switching the training objective from masked-language-modeling to next-token-prediction could tackle this issue, but might require a larger scale architecture and larger training dataset to develop an optimal next-token-prediction model.^75^ Due to the current limitation of context size (Materials and methods), ProMEP works the best for proteins shorter than 1024 aa, which covers ~95.88% Uniparc sequences.^76^ For proteins longer than this threshold, such as the spike protein of SARS-CoV-2 (1273 aa), ProMEP needs to split the protein into overlapped segments and run multiple times to capture the whole sequence context and structure context. With the advent of natural language-processing techniques such as recurrent memory transformer,^77^ we will update a long-context and InDels-compatible version of ProMEP in the future. Presently, ProMEP incorporates both sequence context and structure context of a protein monomer for predicting mutation effects. Notably, the inclusion of PPIs or the prediction of binding affinities between multiple proteins or molecules has not been integrated as a module within ProMEP. The incorporation of PPIs is a promising avenue to broaden the utility and advancement of our model.

In conclusion, ProMEP is a general and MSA-free computational method that enables zero-shot prediction of mutation effects on diverse proteins. ProMEP achieves SOTA performance in a comprehensive suite of benchmarks, with a tremendous improvement in speed. Importantly, ProMEP demonstrates great potential in guiding protein engineering with tens of mutations. ProMEP will enable the exploration of the vast uncharted realms of protein space and greatly benefit studies in biomedicine and synthetic biology.

## Materials and methods

### Construction of protein point cloud

An arbitrary protein structure can be represented as a contact map (a L × L matrix where each element is the distance between two residues of a protein with length L) or a graph (e.g., nodes are residues, and the edges are inter-residue interactions). While a contact map disregards atomic structural information (e.g., coordinates of each atom), a graph contains fine-grained details in protein structures but is often computationally expensive. Point cloud is a commonly used format for 3D data and has shown tremendous success in many areas, including computer vision, autonomous driving and robotics.^78^ A point cloud can be represented as an unordered set of 3D points {P_i_| i = 1, …, n}, where all points are homogeneous and each point P_i_ is a vector of its (*x*, *y*, *z*) coordinates. Compared with contact map and protein graph, point cloud has the advantage of preserving atomic geometric information without sacrificing computational efficiency.

Compared with the naïve point cloud, which is unordered and homogeneous, our proposed protein point cloud consists of ordered and heterogeneous points that are extracted from its raw structure. Specifically, each point corresponds to the alpha C atom of an amino acid. In addition to the 3D coordinates of each point (*x, y, z*), the type of residue each point belongs to (*R*) and the position of each residue in the protein sequence of length L (*P*) is attached as point features.

Definition of each point in protein point cloud: [*x, y, z, R, P*],

R ∈ (G, A, V, L, I, S, T, C, M, D, E, N, Q, R, K, F, Y, W, P, H)

P ∈ (1, 2, 3, …, L)

### Architecture of the multimodal deep representation learning model

To decipher protein functions at the residual resolution, we develop a multimodal protein representation learning model (~659.3 million parameters). It applies an encoder–decoder architecture to simultaneously learn sequence context and structure context from millions of proteins (Supplementary information, Fig. S1). For a protein of length L, the encoder takes the masked sequence and the masked protein point cloud as input and generates a K-dimensional feature vector for each amino acid. The latent representations (L × K) are then fed into the decoder to complete the missing elements of the corrupted sequence and protein point cloud. K is set to 1280 during training and inference.

The sequence embedding module, the transformer encoder module and the sequence decoder module apply similar networks to that of the current protein language models.^23,24^ Specifically, the transformer encoder module is a 33-layer stacked Transformer, and each layer consists of one layer normalization block, one 20-head attention block and one feed-forward network. The sequence decoder, comprising two linear layers with GELU activation and layer normalization, serves to decode the multimodal features (L × K) of a protein into the probability distribution of each token in the alphabet (L × 33).

The global features of a protein tertiary structure should be invariant to arbitrary input poses, which means that 3D translations and rotations of the input protein structure should not affect the output. To guarantee such invariance, we chose the NVIDIA-optimized version of SE(3)-Transformer^79^ as the structure embedding module, which contains one 8-head attention block interspersed with one normalization module, one TFN layer and one max pooling layer. We used 1 layer SE(3)-Transformer for large-scale training. The structure decoder employs a three-layer multi-layer perceptron (MLP) network with ReLU activation to decode the multimodal features (L × K) of a protein into the 3D coordinates of each alpha C atom (L × 3).

To capture the structure context of a protein, the structure embedding module first calculates the K nearest neighborhoods centered on each point as well as their relative positions. Next, an equivariant weight matrix is built upon the Clebsch–Gordon coefficient and spherical harmonics to guarantee the equivariance of point features during transformation. Third, the attention mechanism is applied to pass features between adjacent points. Finally, point features are aggregated and pooled to output the final structure context.

### Model training

We used proteins from the AlphaFold protein structure database as the self-supervised training dataset. It contains ~200 million structures predicted by AlphaFold2. We removed proteins shorter than 64 aa and those with average pLDDT (predicted Local Distance Difference Test) score lower than 70. It deserves to be mentioned that we did not impose a minimum threshold on the average pLDDT of a predicted structure during the inference stage. We randomly selected ~0.5 million proteins for validation. The final training dataset contains ~160 million proteins. Both amino acid sequence and protein point cloud were extracted from the raw protein structure for multimodal training. Since ~95.88% Uniparc sequences contain fewer than 1024 aa, we set the context size to 1024. For proteins longer than 1024 aa, we sampled the start position of amino acids from uniform distribution [1, n – x + 1] where n is the length of protein minus 1024, and x is sampled from uniform distribution [0, n]. For proteins shorter than 1024 aa, padding tokens were appended to their sequence, and random alpha C atoms selected from the raw structures were appended to the extracted protein point clouds.

The extracted amino acid sequence and protein point cloud were then corrupted and recovered by the proposed multimodal model during training. To mask the protein sequence, we randomly sampled 15% of tokens from the sequence after tokenization as utilized in BERT^80^ and ESM.^23^ Each of the sampled token was replaced with a special mask token with 80% probability, a randomly chosen alternate amino acid token with 10% probability, and the original input token (i.e., no change) with 10% probability. To mask the protein point cloud, we referred to the commonly used mask ratio in current point completion networks^81,82^ and chose to mask 256 points (25% of 1024) from the original data. Specifically, we calculated the central point of the protein and chose 256 nearest neighbor points centered on it. We masked the coordinates of these points and trained the proposed multimodal network to automatically recover them.

The loss function is a sum of a categorical cross-entropy (CE) loss and a permutation-invariant chamfer distance (CD) loss.^83^ In particular, the CE loss measures the differences between the model’s predictions and the true token for masked amino acid sequence. The CD loss quantifies the completion results by calculating the average nearest squared distance between the recovered protein point cloud and the ground truth. By minimizing the CE loss and the chamfer distance loss, our proposed model learns high-order representations of a protein in a self-supervised manner.

All layers except the transformer encoder module are initialized from a zero-centered normal distribution with a standard deviation of 0.02. The transformer encoder module is initialized with parameters of ESM1b.^23^ We trained the multimodal deep representation learning model for 380 K steps using Adam optimizer (β1 = 0.9, β2 = 0.999) at initial learning rate 1e−4 with batch size of 480. The learning rate increases linearly during a warm-up period of 10,000 steps. Afterward, the learning rate follows an inverse square root decay schedule. The training was conducted on 15 nodes interconnected with InfiniBand, where each node contains 8 NVIDIA A100 GPUs.

### Benchmarking multimodal representations with function-related datasets

We used 15 function-related datasets (see Supplementary information, Table S3) to benchmark the performance of our multimodal deep representation learning model compared to a comprehensive suite of baselines. We used the standard partition of each dataset and ensured that the training set and the test set were non-redundant. Protein representations generated by our model are either fed into an MLP or integrated into a customized model to make final predictions. According to the downstream network, two types of representations are used, including the residual-level representation of the protein (protein length × 1280), and the molecular-level representation that averaged across the length of the protein (1280). Details are introduced as follows.

#### EC annotation tasks

EC number^84^ is a commonly used classification scheme that specifies the catalytic function of an enzyme by four digits. Four diverse datasets were used for benchmarking. The EC-PDB dataset, which was constructed by Gligorijevi´c et al.,^34^ consists of 19,198 non-redundant proteins and covers 538 third and fourth levels EC numbers of the EC tree. It was partitioned into training, validation and test sets, with approximate ratios of 80%/10%/10%. Proteins in the test set have varying degrees of sequence identity and structure identity (30%, 40%, 50%, 70% and 95%) to the training set (see Supplementary information, Tables S4, S5). Hermosilla et al.^85^ constructed the EC-384 dataset, which contains 37,428 proteins from 384 fourth-level EC numbers. The entire dataset was split into training, validation and test sets. In addition, proteins in each set do not have more than 50% of sequence similarity with proteins from the other sets. The EC-New-392 dataset and the EC-Price-149 dataset are test sets used in contrastive learning-enabled enzyme annotation (CLEAN).^86^ Specifically, EC-New-392 consists of 392 proteins covering 177 different EC numbers from Swiss-Prot released after April 2022. EC-Price-149 is a collection of 149 proteins validated by experiments described by Price et al.^87^ We replaced the raw input of CLEAN with representations generated by our proposed model and kept the model unchanged. Then we used the same training set to train CLEAN (denoted by Ours-CLEAN) and tested its performance on both EC-New-392 and EC-Price-149.

#### GO annotation tasks

GO annotations capture statements about how a gene functions at the molecular level (MF), where in the cell it functions (CC) and what biological processes (BP) it is involved.^88,89^ The GO-MF, GO-CC and GO-BP datasets were constructed by Gligorijević et al.^34^ They only selected GO terms with at least 50 and no more than 5000 samples, forming ~36 K non-redundant PDB chains that cover 489, 320 and 1943 GO terms at different hierarchies in MF, CC and BP, respectively. We used the same partitioning scheme to split these protein sequences into training, validation and test sets, with approximate ratios of 80%/10%/10%. Proteins in the test set have varying degrees of sequence identity and structure identity (30%, 40%, 50%, 70% and 95%) to the training set (see Supplementary information, Tables S4, S5). Furthermore, each protein in the test set has corresponding experimentally determined PDB structures and at least one experimentally determined annotation. Each GO term was treated as a separate label during training and testing. Hierarchies and distances between GO terms were not considered.

#### Cross-species PPI prediction tasks

The cross-species PPI prediction task involved experiments conducted on the D-SCRIPT dataset,^90^ which is derived from the STRING database. This dataset comprehensively covers PPIs across various species, including human, mouse, fly, yeast and *Escherichia coli*. In the human subset, there are ~38,000 PPIs in the training set and 25,000 in the test set. All PPIs from other species were integrated into the test set, with 22,000 PPIs for *E. coli* and 55,000 for fly, yeast and mouse. Negative samples were generated by randomly pairing proteins from the non-redundant set, with their quantity being ten times that of the positive samples. This methodology aligns with the understanding that true PPIs are infrequent. For predictive modeling, a human PPI-trained model was employed to predict PPIs in the test set. Importantly, all protein structures used in this study were sourced from the AlphaFold protein structure database.

#### Virus–human PPI prediction tasks

The virus–human PPI prediction task is conducted in our study. We employed three datasets curated by Dong et al.^91^ to assess the performance of our model in predicting virus–human PPIs. These datasets encompass interactions between human proteins and virus proteins such as Ebola and H1N1. Each dataset comprises thousands of human proteins and hundreds of virus proteins (refer to Supplementary information, Table S6 for details). The structures of human proteins were obtained from the AlphaFold protein structure database, while the prediction of virus protein structures was carried out using ESMFold. The number of both negative and positive PPIs for training in the Ebola dataset was 11,341, while 150 non-redundant PPIs were used as the test set. In the H1N1 dataset, the training split includes 10,858 negative and positive PPIs, while the test split comprises 381 non-redundant instances of negative and positive interactions. In the DENOVO dataset, the training set is composed of 5020 positive samples and 4734 negative samples, and the test split consists of 425 non-redundant instances of positive and negative PPIs.

#### Multi-class PPI prediction tasks

The multi-class PPI prediction task is undertaken using two datasets, namely SHS148K and STRING, curated by GNN-PPI.^92^ These datasets comprise 44,488 and 593,397 multilabel PPIs, respectively. Both two datasets employ two heuristic evaluation schemes based on the PPI network for splitting. Specifically, the Breadth-First Search (BFS) and Depth-First Search (DFS) algorithms are utilized to explore ~20% of the PPIs, forming the test set. All PPIs are categorized into seven types: activation, inhibition, reaction, binding, expression, catalysis and post-translational modifications. Each pair of interacting proteins is associated with at least one of these labels. The protein structures utilized in the analysis are retrieved from the AlphaFold protein structure database, encompassing 5189 proteins in SHS148K and 15,335 proteins in STRING.

For EC-PDB, EC-384, GO-BP, GO-MF, GO-CC, PPI-Mouse, PPI-Fly and PPI-*E. coli*, we respectively constructed an MLP classifier as described in ref. ^33^ to decode the representations generated by different methods (see Supplementary information, Tables S1, S2). For PPI-SHS148K and PPI-STRING, we constructed an eight-layer stacked transformer with a hidden size of 256. While CNN, ResNet, LSTM and Transformer were initialized randomly and trainable,^93,94^ the parameters of other pre-trained models were frozen during training. In particular, pre-trained parameters of UniRep,^7^ ESM1b^23^ and ProtT5^24^ were downloaded and used. Models and results of other pre-trained models were obtained from corresponding publications (Supplementary information, Table S7).^33,85,92,95–99^

For the rest of the datasets, our proposed model acted as a plugin model that generated latent representations for proteins and utilized existing customized models for further prediction. Specifically, we used CLEAN,^86^ for EC-New-392 and EC-Price-149. We replaced the raw input of CLEAN with representations generated by our proposed model and kept the model unchanged. For PPI-DENOVO, PPI-EBOLA and PPI-H1N1, we used multimodal representations as the input of the graph model proposed by Dong et al.^91^ and kept all hyper-parameters unchanged. Results of other methods were obtained from corresponding publications.

We probed the robustness of our proposed model in learning sequence–function and structure–function relationships from proteins with low sequence/structure similarity on downstream tasks. Specifically, BLAST was used to align the protein sequences of the test set to the training sequences and computed the identity score. We used TMScore^100^ to assess the topological similarity between protein structures of the test set and the training set. Five similarity cutoffs were used to partition each test set into multiple groups (see Supplementary information, Tables S4, S5).

### Multimodal context benchmarking

We used three benchmarks to investigate whether the sequence context and the structure context of a protein could be captured by ProMEP.

#### Protein sequence context

We evaluated the micro-structure perception ability of our proposed multimodal network by quantifying its attention on functional sites. During training and inference, it applied the attention mechanism^101^ to probe sequence context and structural context. Calculating the attention score between residues of a protein allowed us to identify key positions that the model focused on. We randomly selected 10,000 proteins from Swiss-Prot and filtered out proteins without functional site annotations. We used the 80% identity-filtered subset, which contains 1325 proteins. We ranked all residues based on the attention score during the evaluation. Specifically, we computed the average attention score across all 33 layers and 20 heads during the quantification of the attention score for each amino acid in the sequence. Examples of attention visualization are shown in Fig. 3c and Supplementary information, Fig. S6a.

DeepFRI,^34^ HEAL^42^ and a random approach (denoted as Random) were used as baselines. Specifically, DeepFRI is a graph convolutional network that employs a pre-trained protein language model to extract sequence features and further constructs a residue graph to predict protein functions. In addition, DeepFRI could identify the contribution of each residue to the predicted function. We utilized the official model checkpoints of DeepFRI and used the Molecular Function branch for evaluation. HEAL is a deep learning model for protein function prediction, which could capture structural features via a hierarchical graph transformer. We downloaded the pre-trained parameters of HEAL and applied the gradient-weighted Class Activation Map (grad-CAM)^102^ to rank the activation score of each residue. Random is a baseline strategy that randomly ranks the importance of each residue. We reported the average performance of Random across five runs. We used the commonly used Top-1 HR, NDCG and MRR as evaluation metrics.

#### Local structure context

To benchmark the ability of the proposed multimodal network to capture local structure context, we used the dataset constructed by Klausen et al.^103^ as the training and validation set. It contains 10,837 crystal structures obtained from PDB that filtered at the 25% identity threshold as well as the 2.5 Å resolution threshold. Among these structures, 10,337 structures were used as the training set, and 500 randomly selected structures were left out as the validation set. CB513,^104^ CASP12^104^ and TS115^105^ were used as test sets, which contain 507, 21 and 115 non-redundant structures, respectively. All proteins in the test set share no more than 25% sequence identity with the proteins present in the training set. Each amino acid of each structure was mapped to 8-class (Q8) secondary structure labels as described in ref. ^106^ Our proposed model acted as an encoder that generated residual-level representations, which were then fed into an MLP classifier as described in ref. ^94^ It is important to acknowledge that during the pre-training stage, our proposed multimodal deep representation learning model processed unlabeled protein structures without prior knowledge of the actual secondary structure labels assigned to each amino acid. Results of other methods were obtained from ref. ^94^ (Supplementary information, Table S7).

We also constructed two small-scale datasets to further benchmark multimodal representations in the condition of scarce training samples. The PDB-100 dataset consists of 100 single-domain proteins that are randomly selected from the SCOP database.^107^ For each protein, we obtained its structure and secondary structure annotations for each of the positions from the PDB.^62^ In addition to the SCOP-100 dataset, we constructed the CATH-100 dataset by randomly selecting 100 proteins from the dataset collected by Zhou et al.^108^ Each protein in CATH-100 has experimentally determined 3D structure as well as annotated B-factor and solvent-accessible surface area for each of the positions. While PDB-100 is a 3-class classification task, CATH-100 corresponds to two regression tasks. We compared the performance of our proposed multimodal network with several leading methods, including UniRep, ESM1b and ProtT5. For each task, we constructed a random forest model to make predictions on the basis of representations generated by each of these methods, respectively. We report the performance of each model in a 5-fold cross-validation manner on the entire dataset. We used the random forest model trained on SCOP-100 for subsequent secondary structure benchmarking and visualization.

#### Global structure context

The SCOPe database organizes protein domains into multiple hierarchies, including Family, Superfamily and Fold.^107^ In particular, the basis of classification for Folds is purely structural. As described in ref. ^109^ we used the 40% identity-filtered subset of SCOPe v2.07 as the benchmark set. It contains 13,265 domains that can be classified into seven classes (see Supplementary information, Table S8). We constructed a five-layer MLP (batch size: 24, learning rate 3e−5, dropped out ratio 0.2, Adam optimizer) as the decoder to classify multimodal representations to a specific fold class. We report the average F1 score of the 5-fold cross-validation results on the entire dataset. Leading structure-based methods were used as baselines, including GraSR and DeepFold. GraSR uses a contrastive learning framework to capture protein features from a protein graph of protein structure.^109^ DeepFold extracts structural motif features from protein contact maps via a deep convolutional neural network.^110^ SGM^103^ and SSEF^104^ are classical structural classification tools that uses 30 global backbone topological measures and frequencies of 1500 secondary structure triplets to encode protein structures, respectively. The performance of these methods are obtained from refs. ^109,110^ (Supplementary information, Table S7).

### Zero-shot protein fitness modeling

ProMEP quantifies the log-likelihood of protein variants under the context of both sequence and structure. The calculation is shown in the equation. To model the fitness of a protein, the WT sequence *S* and structure context *C* are fed into ProMEP, which in turn outputs a sequence of log probabilities. We calculated the conditional probabilities of mutated amino acid *mt* and WT amino acid *wt* at each mutational position *t*. The sum over all mutated positions *T* is the final fitness score of a protein variant.

$${\sum}_{t\in T}\log p({x}_{t}={x}_{t}^{{mt}}{{{{{\rm{|}}}}}}{S}_{-t},C)-\log p({x}_{t}={x}_{t}^{{wt}}{{{{{\rm{|}}}}}}{S}_{-t},C)$$

We first evaluated ProMEP on three representative datasets. Specifically, the UBC9 dataset was constructed by Weile et al.^35^ and comprises experimental data obtained from growth-based complementation assays, where the fitness of 2563 protein variants with single mutations was estimated. Roscoe et al.^36^ constructed the RPL40A dataset that contains 1380 single mutations by quantifying yeast growth rate as a measure of experimental fitness. The protein G dataset^12^ contains 1045 single mutations and 535,917 double mutations with measured binding affinity.

We then used 66 DMS datasets that cover 53 proteins from the ProteinGym benchmark^26^ for the generalization test. All proteins derived from prokaryotes, human and other eukaryotes collected in the ProteinGym benchmark were included. Nineteen proteins derived from viruses were not used for evaluation because of the biases in the pre-training dataset.^30^ We used ESMFold to predict the structure of the WT protein in these datasets. For each WT protein, we collected ~300 homologous sequences from the NR database with sequence identity lower than 80% and predicted their structures via ESMFold. We then used these homologous samples to fine-tune ProMEP for 3 epochs with a learning rate of 1e−4. The fine-tuning procedure enables ProMEP to gain a better understanding of sequence and structure contexts from homologies sampled through evolution. AlphaMissense, which is the best method for mutation effect prediction, was used as a leading baseline. The SOTA protein language model, ESM2_3B and ESM2_650M, as well as the structure-enhanced protein language model, ProstT5, were also evaluated as baseline models. To predict mutation effects with ProtsT5, we first extracted residue embeddings of each protein, and then used VESPA to calculate the logits score. The predicted score of multiple mutations in ProtsT5 was obtained by adding the score of each single mutation. Results of other baseline methods were obtained from the ProteinGym benchmark (Supplementary information, Table S7).^26^

### Pathogenic variant classification

The datasets employed for the pathogenicity prediction task are derived from two distinct sources: the ClinVar test set^111^ and de novo variants identified in individuals with rare diseases.

The ClinVar test set comprises a total of 30,884 pathogenic and 51,988 benign variants, distributed across 7951 proteins. To validate the ProMEP and AlphaMissense models on proteins characterized by shallow MSA depths, proteins with fewer high similarity sequences were selected. Specifically, we ascertained the number of high similarity sequences for proteins within the ClinVar dataset by aligning them with the Non-Redundant Protein Database using BLAST. Hits with an identity below 0.95 or coverage below 0.5 were systematically filtered out. We adhered to the criteria established by AlphaMissense,^28^ wherein proteins are retained only if they meet a minimum threshold of five benign and five pathogenic variants. We chose proteins with high similarity sequences < 100.

The second dataset is composed of de novo variants identified within both patients and healthy controls participating in the DDD cohort.^43^ This dataset comprises a total of 410 variants distributed across 156 proteins.

In the pathogenicity prediction task, ProMEP is not fine-tuned. For a given position *i* within the protein, the pathogenic likelihood of a residue undergoing mutation from amino acid type *w* to type *m* is calculated as follows:

1 $${P}_{i}\left({w|m}\right)={{{{{\rm{Sigmoid}}}}}}\left({{logit}}_{i}^{w}-{{logit}}_{i}^{m}-\tau \right)$$

where *logit* represents the output of ProMEP, *τ* signifies a handcraft threshold. Specifically, *τ* is set to 6 during evaluation. A variant is considered pathogenic if *P*_*i*_ (w|m) > 0.5.

In addition to AlphaMissense, we performed a comparative analysis with several MSA-free baseline models, including ESM1b, ESM1v and Tranception. Pathogenicity calculations for ESM1b and ESM1v were conducted utilizing Eq. 1, with *τ* maintained consistently in accordance with ProMEP guidelines. Equation 1 is similarly applied to Tranception; however, *τ* is specifically set to 0. It is noteworthy that the hyperparameter *τ* exclusively impacts the accuracy metric, but with no discernible influence on the auROC metric.

### ProMEP vs AlphaMissense and GEMME timing experiments

We randomly collected proteins with lengths from 100 aa to 1000 aa from UniProt. We compared the model inference of ProMEP and AlphaMissense. Time costs of preparing input for the model (e.g., searching MSAs from sequence databases in AlphaMissense, or predicting protein structure from protein sequence) and model initialization (e.g., Jax graph compilation times in AlphaMissense, or pre-trained weight loading times in ProMEP) were excluded. Since the trained AlphaMissense model weights are not released, we used randomly initiated weights during evaluation and did not count the weight loading times in AlphaMissense. We evaluated the model inference time of predicting mutation effects via ProMEP or AlphaMissense. For each length, we report the average time costs of at least three proteins. All experiments were run on a single NVIDIA V100 GPU.

### ProMEP-guided protein engineering of TnpB

We began by ranking protein variants with single mutation. Specifically, we constructed a virtual saturation mutagenesis library that only contains single variants (7752 variants). We then ranked all variants via the calculated fitness score. Since X-to-R mutations (e.g., S72R) are commonly used in the engineering of CRISPR-Cas proteins, we chose the top 10 beneficial X-to-R variants and top 10 deleterious X-to-R variants from the entire ranked list for further evaluation. We also constructed a virtual mutagenesis library that consists of triple X-to-R mutants (8,510,740 mutants). Again, we calculated the fitness score of each variant. According to the experimental data from top 10 beneficial X-to-R single mutants, we filtered out mutants that contain neutral or negative mutations (Y388R, S217R, L398R, T405R, L406R, K44R and H403R) from top-ranked beneficial mutants. The top 10 beneficial triple mutants from the mutagenesis library were selected for further evaluation. *P* values were derived by a two-tailed Student’s *t*-test. All statistical analyses were performed on *n*  =  3 biologically independent experiments.

To generate variants with more mutations, we fine-tuned ProMEP based on the experimental results of TnpB mutants. The primary aim is to distinguish beneficial mutations from deleterious ones. In particular, TnpB mutants exhibiting a fold change > 1.2 were designated as positive samples, while those displaying a fold change < 0.8 were classified as negative samples. The fine-tuning dataset, consisting of 27 samples, was divided into a training set (80%) and a validation set (20%). We used a binary CE loss during the fine-tuning process:

$${{{{{\rm{loss}}}}}}=-\left(y\log P\left(x\right)+\left(1-y\right)\left(\log \left(1-P\left(x\right)\right)\right)\right)$$

Specifically, the label of each sample is denoted as *y* (where *y* = 1 signifies beneficial mutations and *y* = 0 signifies deleterious mutations). $P\left(x\right)={{\mbox{Sigmoid}}}\left({m}_{t}-{w}_{t}\right)$, is the predicted fitness score determined by ProMEP, in which *w*_*t*_ and *m*_*t*_ is the logits of WT and mutation types, respectively.

### ProMEP-guided protein engineering of TadA

We constructed a virtual saturation mutagenesis library that only contains single mutation for TadA. To preserve its specialty as a deoxyadenosine deaminase, mutations at positions 106 and 108 were filtered out. We calculated the fitness scores for the rest 3135 variants and chose the top 10 beneficial variants and top 10 deleterious variants for further evaluation.

To guide the screening of TadA mutants with more than 10 mutations, we utilized ProMEP to identify the top 40 beneficial single mutants and assessed their editing efficiency (Supplementary information, Data S3). Based on these experimental data, we constructed two variants, namely, TadA-AI-8 and TadA-AI-14, which incorporate 8 and 14 mutations, respectively. Subsequently, we used ProMEP to investigate three subspace of TadA mutants, and selected 40 mutants with the highest fitness scores.

### Plasmid vector construction

Plasmid amino acid sequences are listed in Supplementary information, Data S4. The *TnpB* gene was optimized for expression in human cells through codon optimization, and the optimized sequence was synthesized for vector construction by Sangon Biotech. We inserted the ultimately optimized sequence into the pST1374 vector, which contains the CMV promoter and a nuclear localization signal. The construction of TnpB mutants is achieved through site-directed mutagenesis. PCR amplifications were performed using Phanta Max Super-Fidelity DNA Polymerase (Vazyme). Following digestion with *Dpn*I (New England BioLabs), the PCR products were then ligated using 2× MultiF Seamless Assembly Mix (ABclonal). Ligated products were transformed into DH5α *E. coli* cells. The success of the mutations was confirmed via Sanger sequencing. The modified plasmid vectors were purified using a TIANpure Midi Plasmid Kit (TIANGEN). ABE-dTnpB-WT and ABE-dTnpB-AI were generated as described previously, albeit with procedural modifications.^112^ TadA-TadA* sequences were fused at 3′-region of dead TnpB (TnpB^D191A^) or dead TnpB-AI-5.6 (TnpB^D191A/S72R/K84R/E168R/K251R/V374R^) with 32-aa linkers using 2× MultiF Seamless Assembly Mix (ABclonal). The CBE-dTnpB-WT and CBE-dTnpB-AI vectors were constructed by insertion of dTnpB or dTnpB-AI between human APOBEC3A(Y130F^113^) and two UGIs. Various mutants of TadA sequences were synthesized by Sangon Biotech. Then, TadA and its variants were cloned into a vector containing nCas9(D10A) and nuclear localization signal. All guide RNA plasmids were cloned using T4 DNA Ligase (New England Biolabs). Oligos for targeting spacers were annealed and ligated into *Bsa*I (New England BioLabs)-digested PGL3-U6 backbone vectors. The spacer sequences of guide RNA used in the study are shown in Supplementary information, Data S5. The final constructed vectors were all validated for accuracy by Sanger sequencing.

### Cell culture and transfection

HEK293T cells were maintained in Dulbecco’s modified Eagle medium (Gibco) supplemented with 10% fetal bovine serum (Gemini) and 1% penicillin–streptomycin (Gibco) in an incubator (37 °C, 5% CO_2_). For InDel analysis, HEK293T cells were transfected at 80% confluency with a density of ~1 × 10^5^ cells/well in a 24-well plate. For TnpB InDel analysis, 500 ng of TnpB plasmid, 500 ng of reRNA plasmid were co-transfected into HEK293T cells using ExFect Transfection Reagent (Vazyme). For base editing, 500 ng of base editor plasmid and 500 ng of sgRNA plasmid were co-transfected into HEK293T cells using ExFect Transfection Reagent following the manufacturerʼs protocol.

### DNA extraction and deep sequencing

The transfected cells as described above, are washed with PBS (Gibco) and extracted using QuickExtract DNA Extraction Solution (Lucigen). Samples are incubated at 65 °C for 60 min and heat-inactivated at 98 °C for 3 min. The lysed products were used as templates for the first round PCR (PCR1). PCR1 is conducted with PCR1-primers (see Supplementary information, Data S5) to amplify the genomic region of interest using Phanta Max Super-Fidelity DNA Polymerase (Vazyme). PCR1 was performed under the following cycle conditions: 98 °C for 3 min, (98 °C 15 s, 60 °C 15 s, 72 °C 30 s) × 29, 72 °C for 3 min. Following the confirmation of successful PCR1 amplification through gel electrophoresis, the PCR1 products were pooled in equal moles and then purified, getting them ready for the second round of PCR (PCR2). The PCR2 products were amplified using index primers (Vazyme) and purified by FastPure Gel DNA Extraction Mini Kit (Vazyme) for sequencing on the Illumina NovaSeq platform. PCR2 was performed under the following cycle conditions: 98 °C for 45 s, (98 °C 15 s, 60 °C 15 s, 72 °C 30 s) × 6, 72 °C for 3 min. InDel frequencies, A-to-G or C-to-T conversions at each target site were analyzed using CRISPResso2 (https://github.com/pinellolab/CRISPResso2).

### DNA off-target analysis

To evaluate the specificity of TnpB, TnpB-AI and TadA variants, we employed CRISPR RGEN Tools (Cas-OFFinder, http://www.rgenome.net/cas-offinder/) to predict potential off-target sites. For TnpB, the PAM of research was set to “TTGAT” and the mismatches were set to 5. For base editing, the PAM was defined as “NGG” with a mismatch tolerance of 4. Subsequently, we retrieved 1000-bp sequences encompassing these potential off-target sites from UCSC (https://genome.ucsc.edu/) and designed suitable primers for amplifying these specific sequences. Targeted deep sequencing was conducted to evaluate off-target efficiencies. The primers used to amplify potential off-target sites are listed in Supplementary information, Data S6.

### R-loop assay for Cas9-independent DNA off-target analysis

In general, a base editor-expressing plasmid and SpCas9 sgRNA were co-transfected into HEK293T cells in 24-well plates along with nSaCas9 and nSaCas9 sgRNA plasmids at each R-loop site, followed by culturing for 72 h. After 72 h, the transfected cells were digested with 0.25% trypsin (Gibco). Genomic DNA was isolated utilizing QuickExtract DNA Extraction Solution (Lucigen).

### RNA sequencing experiments

For the RNA sequencing experiment, HEK293T cells were seeded in 10-cm dishes. 10 μg base editor plasmid and 10 μg of sgRNA plasmid were co-transfected into HEK293T cells using ExFect Transfection Reagent. After 3 days, cells were washed with 1× PBS, lysed with RNAiso Plus (TaKaRa). Total RNA was extracted utilizing the Trizol method, subsequently assessed for purity using NanoDrop One, and its integrity was evaluated with Agilent 2100. Following this, mRNA enrichment and purification from eukaryotic total RNA were executed with the VAHTS mRNA Capture Beads kit (Vazyme), and RNA was fragmented through ion interruption to attain insert sizes ranging from 250 bp to 450 bp. cDNA first-strand synthesis is conducted using fragmented RNA as a template, succeeded by second-strand cDNA synthesis employing the first-strand cDNA as a template, and subsequent double-stranded cDNA end repair and dA-tailing. Following the ligation of universal adapters, bead-based purification was utilized, and fragment selection was executed for sizes ranging between 250 bp and 350 bp. PCR amplification was conducted, incorporating primer double-end indexes, and the products underwent bead-based purification to yield the complete library. Second-generation sequencing technology, leveraging the Illumina NovaSeq 6000 sequencing platform, was employed for paired-end sequencing of the library.

### Transcriptome-wide RNA analysis

The raw data were processed by fastp v0.23.4 with adapter trimming, low-quality base trimming (-q 20, -r, -W 10, -c), low complexity filtering and length filtering (-l 75). The clean data were aligned to the reference genome hg38 by using hisat2 v2.2.1. Samtools v1.18 was used to sort and index mapping results. The sorted mapping results underwent duplicate marking and base quality recalibration using MarkDuplicates, BaseRecalibrator and ApplyBQSR in GATK toolkit v4.2.5.0. The variants were detected using HaplotypeCaller. The single nucleotide polymorphism (SNP) variants were further filtered to retain SNP variants with base-quality score > 25, mapping quality score > 20, Fisher strand values < 30.0, qual by depth values > 2.0 and sequencing depth > 20. The depth for a given off-target edit should be at least 10× and these edits are required to have at least 99% of reads supporting the reference allele in the WT samples.

### Supplementary information

Supplementary information, Figure S1

Supplementary information, Figure S2

Supplementary information, Figure S3

Supplementary information, Figure S4

Supplementary information, Figure S5

Supplementary information, Figure S6

Supplementary information, Figure S7

Supplementary information, Figure S8

Supplementary information, Figure S9

Supplementary information, Figure S10

Supplementary information, Figure S11

Supplementary information, Figure S12

Supplementary information, Figure S13

Supplementary information, Table S1

Supplementary information, Table S2

Supplementary information, Table S3

Supplementary information, Table S4

Supplementary information, Table S5

Supplementary information, Table S6

Supplementary information, Table S7

Supplementary information, Table S8

Supplementary information, Data S1

Supplementary information, Data S2

Supplementary information, Data S3

Supplementary information, Data S4

Supplementary information, Data S5

Supplementary information, Data S6

## Data Availability

Protein structures used for training are publicly available in AlphaFold protein structure database (https://www.alphafold.ebi.ac.uk/). Public datasets that we used for performance evaluation are obtained from corresponding publications. Please refer to Materials and methods for more details. The deep sequencing data from this study have been submitted to the National Center for Biotechnology Information Sequence Read Archive database under accession number GSE261254, PRJNA1080466 and PRJNA1080297. Source data are provided with this paper (https://github.com/wenjiegroup/ProMEP).

## Code availability

The source codes of ProMEP are available at https://github.com/wenjiegroup/ProMEP. We predicted protein structures via ESMFold (https://github.com/facebookresearch/esm) and AlphaFold2 (https://github.com/deepmind/alphafold). Deep sequencing data were analyzed with CRISPResso2 (https://github.com/pinellolab/CRISPResso2). Protein structures were visualized with PyMOL (https://pymol.org/2/). Sequence alignments were visualized with ESPript (https://espript.ibcp.fr/ESPript/ESPript/). The probability distributions of all amino acid types at different positions were visualized with WebLogo (https://weblogo.threeplusone.com/).
