# Supplementary material for: Zero-shot prediction of mutation effects with multimodal deep representation learning guides protein engineering
Source: Cell Res. 2024 Jul 5;34(9):630–47. doi: 10.1038/s41422-024-00989-2 (PMC11369238; doi:10.1038/s41422-024-00989-2)
Supplement: Supplementary file 1 — Supplementary information, Figure S1 [file 41422_2024_989_MOESM1_ESM.pdf]

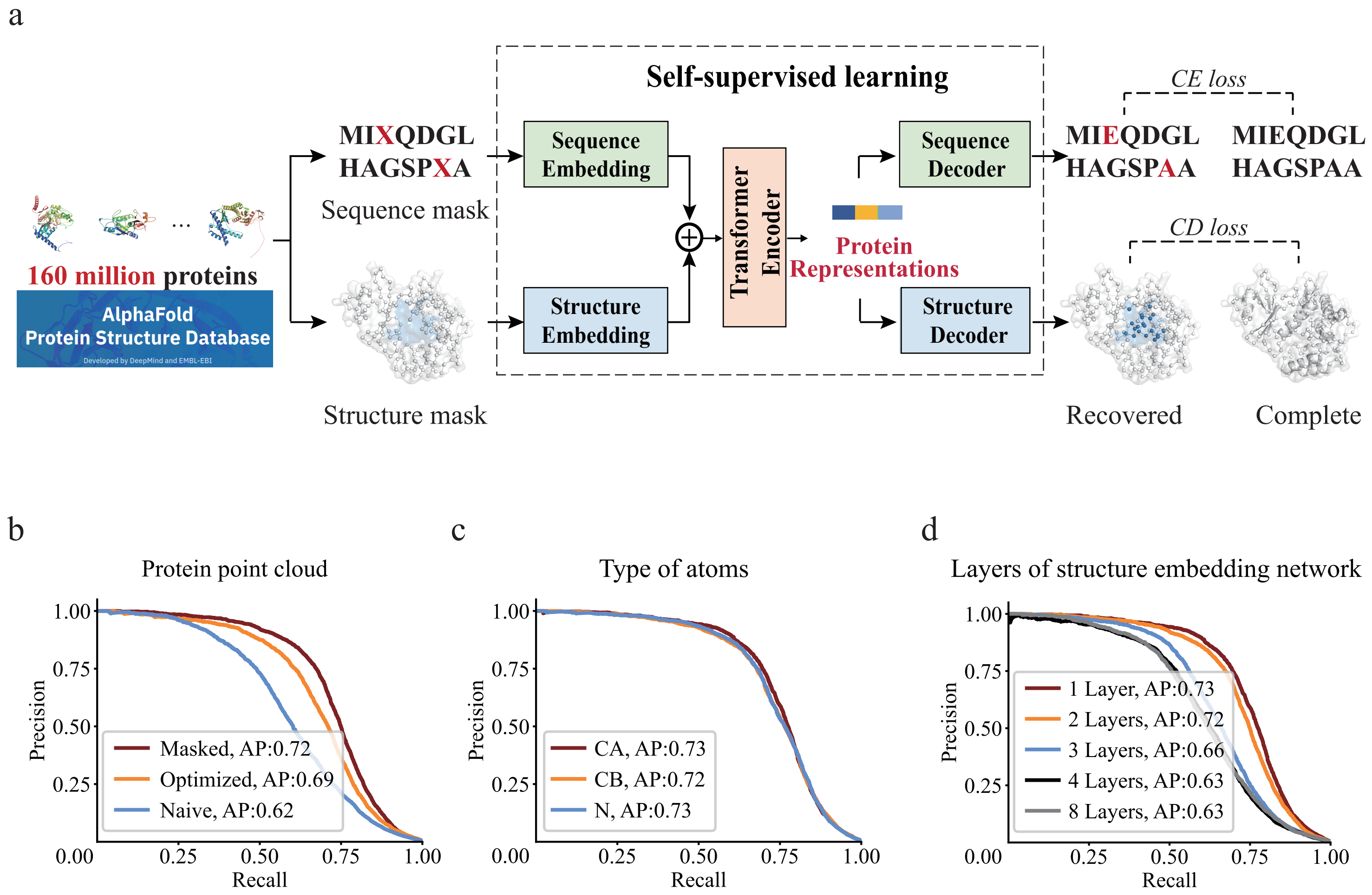


**Figure S1 | A multimodal deep representation learning network that encodes protein functions. a,** It utilizes an encoder-decoder framework to learn both sequence context and structural constraints from ~160 million proteins. During training, each protein is transformed into a corrupted amino acid sequence and a corrupted protein point cloud by replacing a fraction of elements with the mask token or zeroized point, respectively. By completing the missing elements of the corrupted sequence and protein point cloud, it is trained to generate the latent representations of a protein. The Cross-Entropy (CE) loss and the Chamfer Distance (CD) loss are used to measure the difference between the recovered protein sequence and structure to their corresponding complete ground truth, respectively. **b-c,** Design of the protein point cloud and the structure embedding module. For each type of protein point cloud, we train a sub-network that only contains the structure embedding module and structure decoder module. Each sub-network takes a specific type of point cloud as input to learn protein representations. The enzyme commission number annotation benchmark is used for performance evaluation (Methods). We compare the representations generated by the structure embedding module via an MLP classifier and report the average precision (AP). Performance of protein point cloud that contains diverse point features **(b)**. Compared with the naive unordered and homogeneous point cloud (Naive), the optimized protein point cloud (Optimized) is an ordered and heterogeneous set of alpha C atoms that belongs to each amino acid. The type of the residue and the connection between them are preserved. The protein point cloud without residue annotation is denoted as Masked. The optimized protein point cloud demonstrated significantly higher performance compared to the naive point cloud, which only includes coordinate information. Additionally, masking the type of residue attached to each point leads to further performance improvement due to the increased training difficulty. Type of atom used in the protein point cloud **(c).** Protein point cloud constructed by alpha C (CA) atoms, beta C (CB) atoms and N atoms are evaluated, respectively. The protein point cloud constructed using alpha C atoms outperforms those constructed using beta C and N atoms. Number of layers of the structure embedding module **(d).** The single-layer structure embedding module exhibits the best performance in generating structure embeddings of a protein. The masked protein point cloud constructed by alpha C atoms is used during the evaluation. Interestingly, protein representations generated by either a single SE(3)-Transformer layer or two SE(3)-Transformer layers demonstrated exceptional performance, consistent with the utilization of a 2-layer SE(3)-Transformer architecture in RoseTTAFold. This outcome might be attributed to the robust embedding capabilities of the SE(3)-Transformer layer, although there is a possibility of encountering overfitting with an abundance of layers.
