# Supplementary material for: Zero-shot prediction of mutation effects with multimodal deep representation learning guides protein engineering
Source: Cell Res. 2024 Jul 5;34(9):630–47. doi: 10.1038/s41422-024-00989-2 (PMC11369238; doi:10.1038/s41422-024-00989-2)
Supplement: Supplementary file 2 — Supplementary information, Figure S2 [file 41422_2024_989_MOESM2_ESM.pdf]

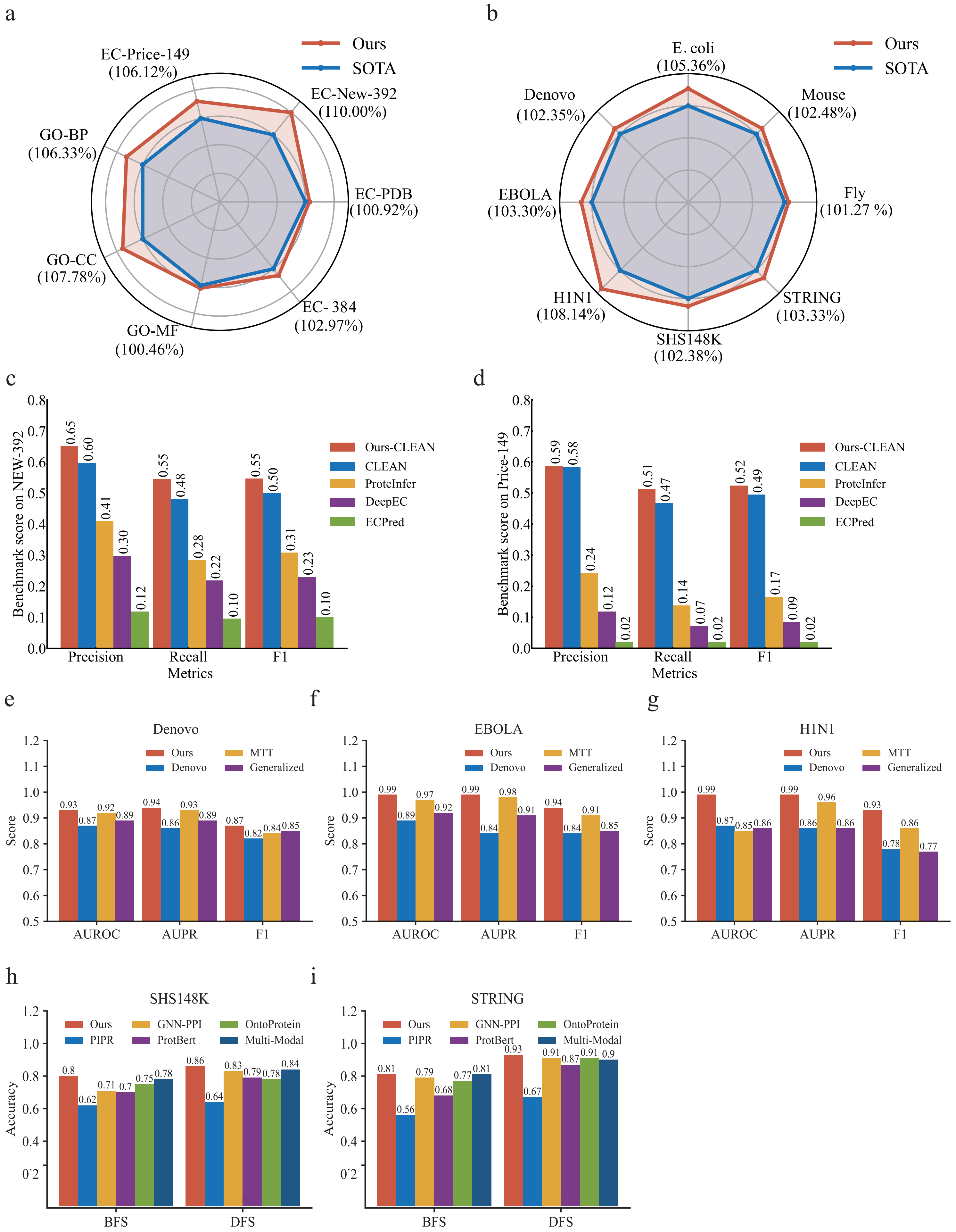
a
Ours
SOTA
EC-Price-149 (106.12%)
EC-New-392 (110.00%)
EC-PDB (100.92%)
EC- 384 (102.97%)
GO-MF (100.46%)
GO-CC (107.78%)
GO-BP (106.33%)
b
Ours
SOTA
E. coli (105.36%)
Mouse (102.48%)
Fly (101.27 %)
STRING (103.33%)
SHS148K (102.38%)
H1N1 (108.14%)
EBOLA (103.30%)
Denovo (102.35%)
c
Benchmark score on NEW-392
Ours-CLEAN
CLEAN
ProteInfer
DeepEC
ECPred
0.65 0.60 0.41 0.30 0.12
0.55 0.48 0.28 0.22 0.10
0.55 0.50 0.31 0.23 0.10
Precision
Recall
F1
Metrics
d
Benchmark score on Price-149
Ours-CLEAN
CLEAN
ProteInfer
DeepEC
ECPred
0.59 0.58 0.24 0.12 0.02
0.51 0.47 0.14 0.07 0.02
0.52 0.49 0.17 0.09 0.02
Precision
Recall
F1
Metrics
e
Denovo
Ours
Denovo
MTT
Generalized
Score
0.93 0.87 0.92 0.89
0.94 0.86 0.93 0.89
0.87 0.82 0.84 0.85
AUROC
AUPR
F1
f
EBOLA
Ours
Denovo
MTT
Generalized
Score
0.99 0.89 0.97 0.92
0.99 0.84 0.98 0.91
0.94 0.84 0.91 0.85
AUROC
AUPR
F1
g
H1N1
Ours
Denovo
MTT
Generalized
Score
0.99 0.87 0.85 0.86
0.99 0.86 0.96 0.86
0.93 0.78 0.86 0.77
AUROC
AUPR
F1
h
SHS148K
Ours
PIPR
GNN-PPI
ProtBert
OntoProtein
Multi-Modal
Accuracy
0.8 0.62 0.71 0.7 0.75 0.78
0.86 0.64 0.83 0.79 0.78 0.84
BFS
DFS
i
STRING
Ours
PIPR
GNN-PPI
ProtBert
OntoProtein
Multi-Modal
Accuracy
0.81 0.56 0.79 0.68 0.77 0.81
0.93 0.67 0.91 0.87 0.91 0.9
BFS
DFS

Figure S2 | Our proposed multimodal network achieves state-of-the-art performance on downstream protein function prediction tasks. a-b, Performance of the multimodal representations (denoted as Ours) compared to a suite of baselines, including ESM, ProTrans and UniRep. 7 protein function prediction datasets **(a)** and 8 PPI prediction datasets **(b)** are used for evaluation. Specifically, we report the Fmax score on the EC-PDB, GO-BP, GO-MF, and GO-CC datasets. F1 score is reported in EC-Price-149, EC-New-392, *Fly, Mouse, E. coli*, Denovo, *EBOLA* and *H1N1* datasets. Accuracy is reported in the EC-384, SHS148K, and STRING datasets. Both ESM and GearNet surpass an Fmax score of 0.86 on the EC-PDB dataset, exhibit significant performance enhancements compared to traditional deep learning approaches (Supplementary information, Table S1) and might approach peak performance levels. Additionally, in the case of the EC-384 dataset, both ESM and GearNet achieve an accuracy score exceeding 0.83. Our proposed model yields competitive outcomes when compared to current leading methodologies on these datasets and displays superior performance on datasets with greater potential for performance enhancement, such as EC-Price-149 and EC-New-392. While our proposed model outperforms current leading methods in the GO-BP and GO-CC datasets, the improvement in the GO-MF dataset is not as pronounced, likely due to the neglection of hierarchies and distances between GO terms. In the task of predicting PPIs, ProMEP demonstrates notable advancements on datasets such as *H1N1* and *E. coli* compared to preceding SOTA, thereby manifesting its superior generalization ability. The efficacy of ProMEP is particularly evident in contexts where there exist notable dissimilarities in distribution patterns between the test datasets and the training data. For instance, the *E. coli* test set exhibits fewer distributional similarities with the training set, whereas the Mouse and Fly datasets share more. Consequently, ProMEP's performance excels notably on the *E. coli* test set. **c,** We integrate our multimodal representations to CLEAN (denoted as Ours-CLEAN) and evaluate its performance on the New-392 database. **d,** Comparison of CLEAN-Ours, CLEAN, and other top-ranked models on the Price-149 database. **e-g,** Performance comparison for binary PPI prediction, including AUROC, AUPR, and F1 scores, on three virus-human PPI datasets, Denovo **(e),** *EBOLA* **(f),** and *H1N1* **(g). h-i,** Performance comparison for multi-class PPI prediction on the SHS148K **(h)** and STRING **(i)** dataset. All models are evaluated using two heuristic schemes: breadth-first search (BFS) and depth-first search (DFS).
