# Supplementary material for: Zero-shot prediction of mutation effects with multimodal deep representation learning guides protein engineering
Source: Cell Res. 2024 Jul 5;34(9):630–47. doi: 10.1038/s41422-024-00989-2 (PMC11369238; doi:10.1038/s41422-024-00989-2)
Supplement: Supplementary file 3 — Supplementary information, Figure S3 [file 41422_2024_989_MOESM3_ESM.pdf]

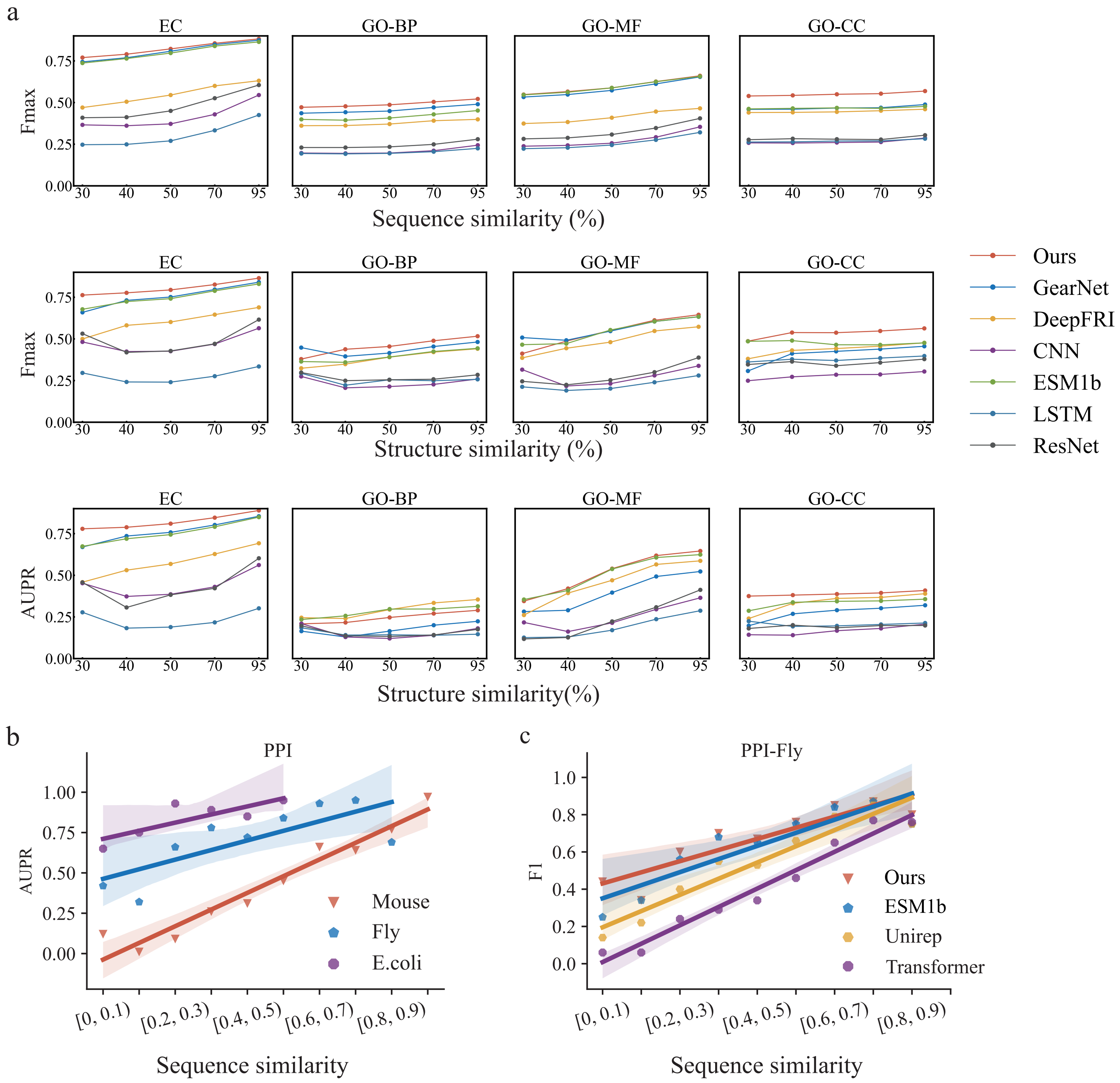

Figure S3 | Robustness tests on downstream tasks. a, Robustness test of our proposed model (denoted as Ours) and several important baselines under different sequence/structure similarity cutoffs between training and test sets (Methods). **b, P**erformance of our model on test PPIs of three species (*Mouse*, *Fly*, and *E.coli*) in D-SCRIPT with regard to the sequence identity to the training set. **c,** Performance comparison on the *Fly* PPIs of D-SCRIPT with regard to the sequence identity to the training set.
