# Supplementary material for: Zero-shot prediction of mutation effects with multimodal deep representation learning guides protein engineering
Source: Cell Res. 2024 Jul 5;34(9):630–47. doi: 10.1038/s41422-024-00989-2 (PMC11369238; doi:10.1038/s41422-024-00989-2)
Supplement: Supplementary file 4 — Supplementary information, Figure S4 [file 41422_2024_989_MOESM4_ESM.pdf]

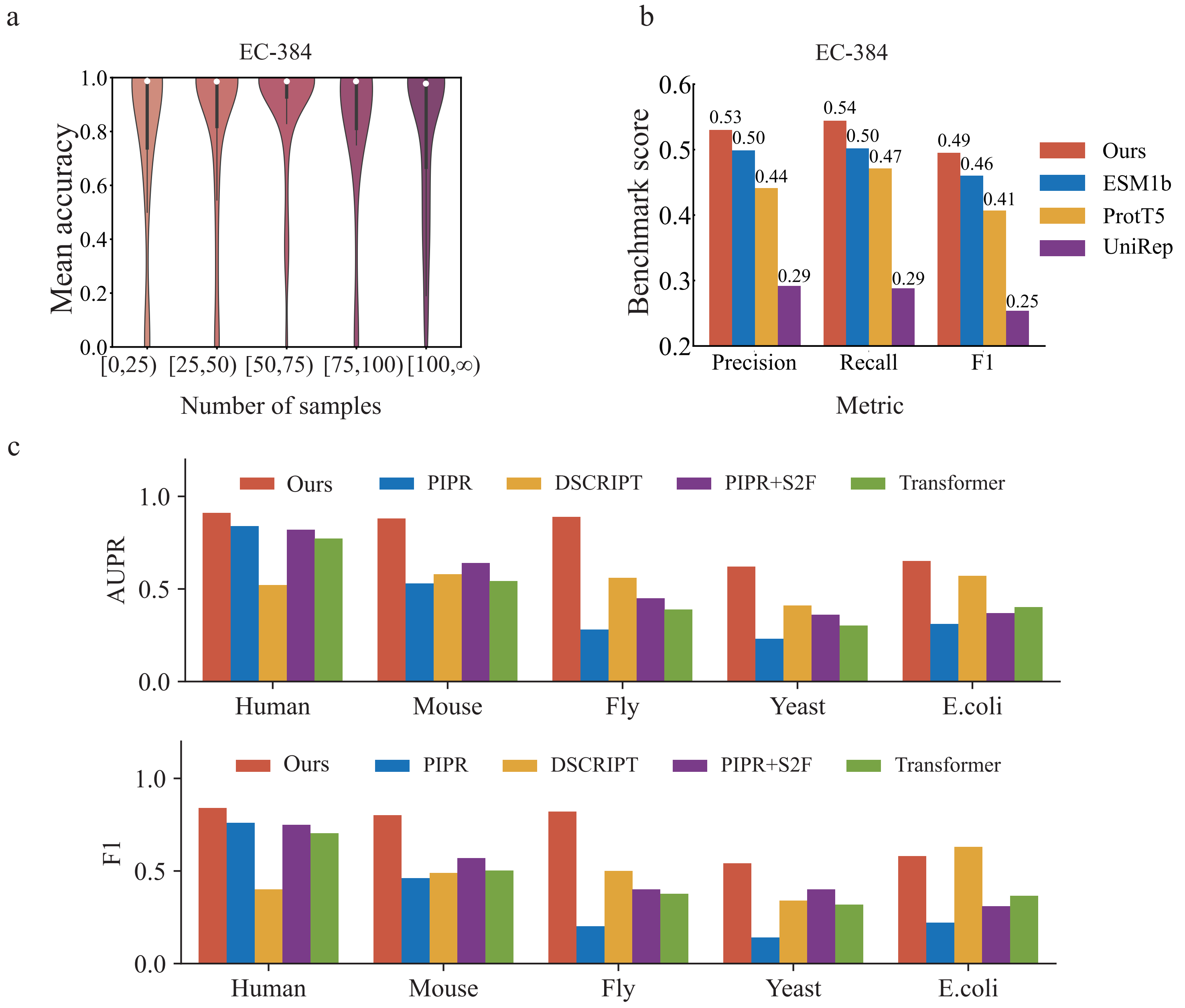

Figure S4 | Extensive generalization tests on downstream tasks. a, A violin plot that shows the mean accuracy of our proposed model on 384 EC classes with different numbers of proteins in the training set. **b,** Comparison of our proposed model (denoted as Ours) and several important baselines in one-shot EC number prediction. Each EC number in the training set contains only one protein. **c,** Performance comparison for binary PPI prediction on the cross-species PPI dataset.
