# Supplementary material for: Zero-shot prediction of mutation effects with multimodal deep representation learning guides protein engineering
Source: Cell Res. 2024 Jul 5;34(9):630–47. doi: 10.1038/s41422-024-00989-2 (PMC11369238; doi:10.1038/s41422-024-00989-2)
Supplement: Supplementary file 5 — Supplementary information, Figure S5 [file 41422_2024_989_MOESM5_ESM.pdf]

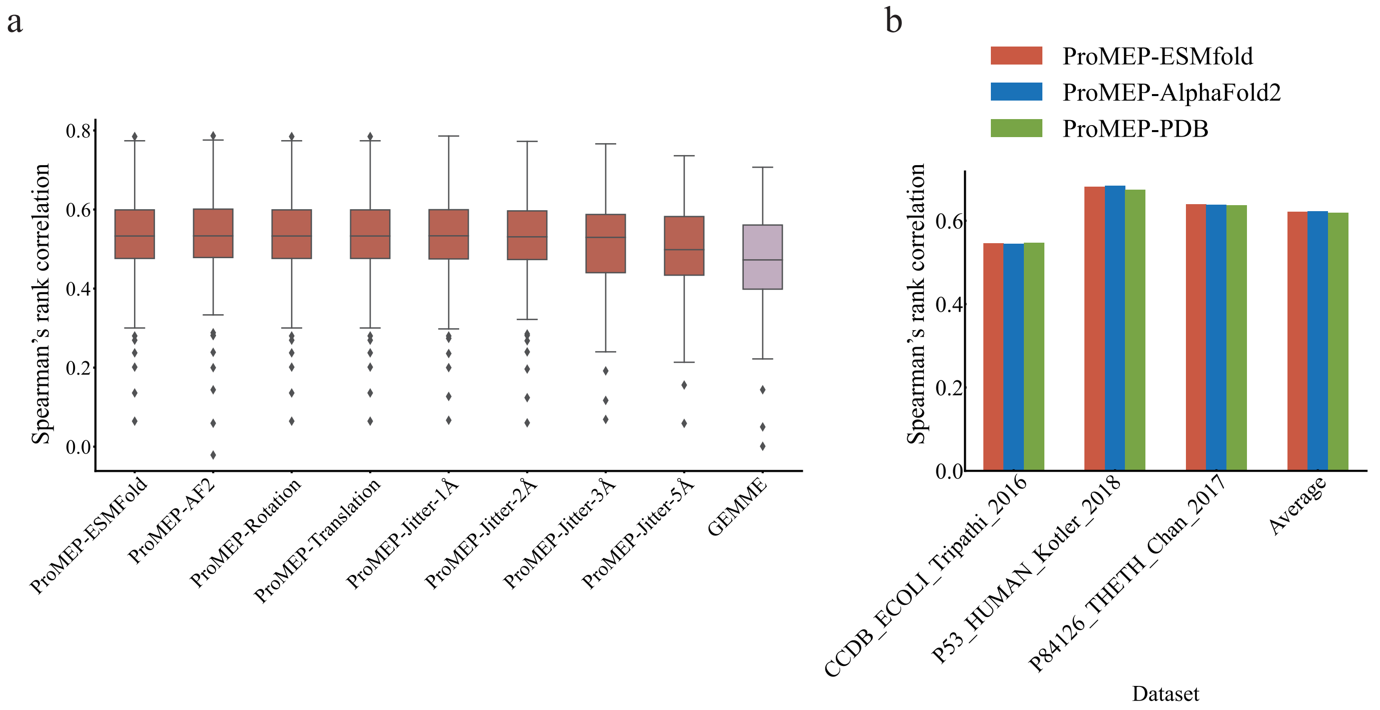

Figure S5 | Performance of ProMEP that uses different sources of protein structure data on the ProteinGym benchmark. a, ProMEP tolerates structure noises when predicting mutation effects. It shows similar performance when input structures are predicted by ESMFold (ProMEP-ESMFold) or AlphaFold2 (ProMEP-AF2). We also evaluate how ProMEP performs when 3D rotation, translation or structure noises are introduced to the input structures (Methods). **b,** Three proteins that have complete structures determined by experiments are further analyzed. Performance of ProMEP that uses structures predicted by ESMFold, AlphaFold2 and experiments-determined structures is annotated as ProMEP-ESMFold, ProMEP-AlphaFold2 and ProMEP-PDB, respectively.
