# Supplementary material for: Zero-shot prediction of mutation effects with multimodal deep representation learning guides protein engineering
Source: Cell Res. 2024 Jul 5;34(9):630–47. doi: 10.1038/s41422-024-00989-2 (PMC11369238; doi:10.1038/s41422-024-00989-2)
Supplement: Supplementary file 6 — Supplementary information, Figure S6 [file 41422_2024_989_MOESM6_ESM.pdf]

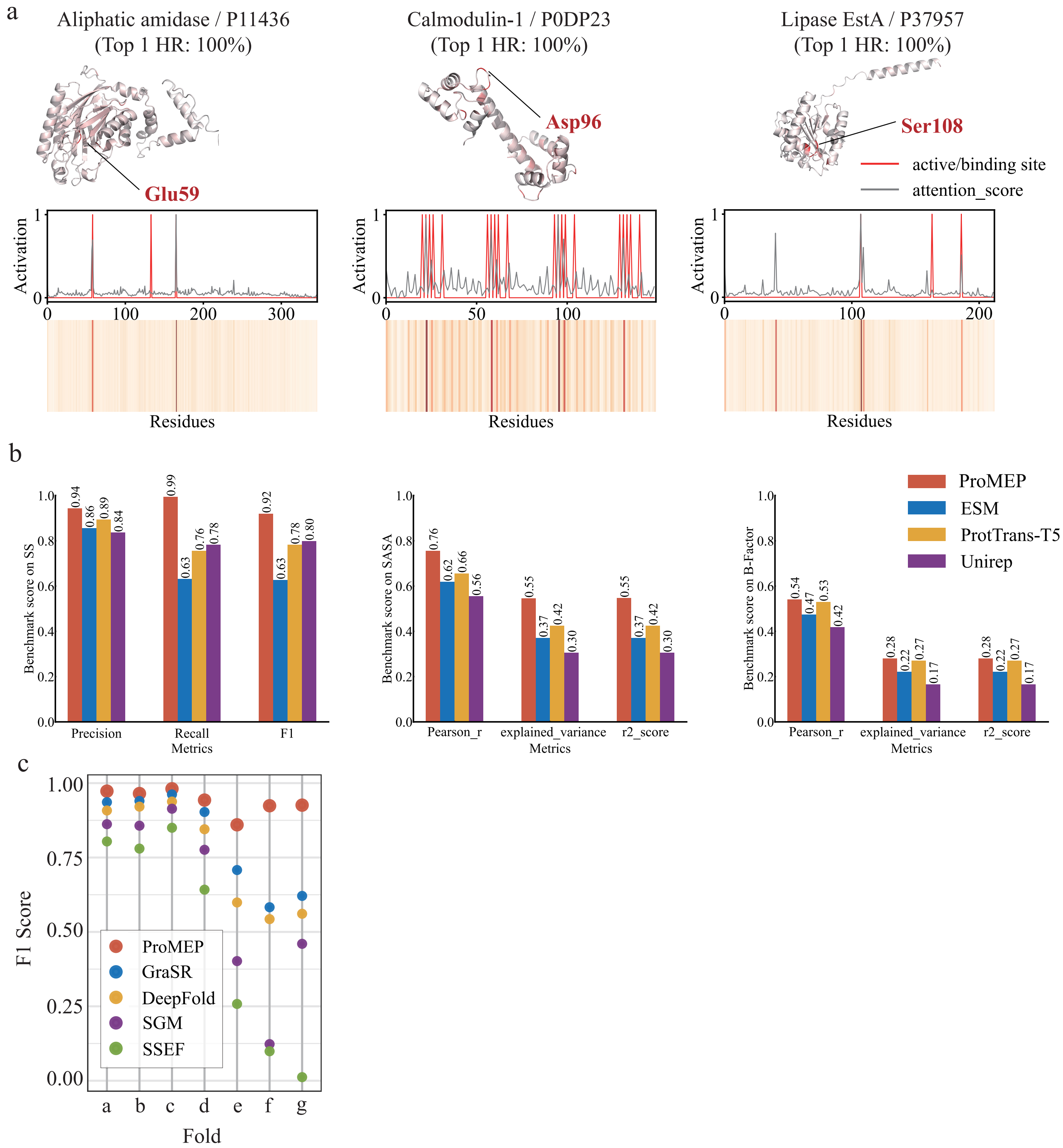

Figure S6 | Extensive evaluations of the multimodal context captured by ProMEP. a. Visualization of interactions between sequential amino acids (sequence context) on three proteins in the ProteinGym benchmark. Top: More salient residues are highlighted in red in the presented structure. Bottom: The quantified interaction score of each position is also presented in the functional sites identification map. Actual functional sites (Active/binding sites) are labeled as the red line. The interaction scores are labeled as the grey line. **b,** Three additional benchmarks related to local structure context, which include 3-class secondary structure classification on the PDB-100 dataset, solvent accessible surface area (SASA) prediction, and b-factor prediction on the CATH-100 dataset (Methods). Specifically, SASA and b-factor are closely related to protein folding and stability. We report the precision, recall and F1 score of different methods on the PDB-100 dataset. We report the Pearson correlation (Pearson_r), the explained variance and the R2 score (r2_score) of different methods on the CATH-100 dataset. **c,** Performance of ProMEP on a 7-class fold classification benchmark (Methods). It contains 13,265 domains that can be classified into seven classes, including all alpha proteins (*class a*), all beta proteins (*class b*), alpha and beta proteins (*a/b, class c*), alpha and beta proteins (*a+b, class d*), multi-domain proteins (*class e*), membrane and cell surface proteins and peptides (*class f*) and small proteins (*class g*). We report the F1 score of the 5-fold cross-validation results on the entire dataset.
