# Supplementary material for: Zero-shot prediction of mutation effects with multimodal deep representation learning guides protein engineering
Source: Cell Res. 2024 Jul 5;34(9):630–47. doi: 10.1038/s41422-024-00989-2 (PMC11369238; doi:10.1038/s41422-024-00989-2)
Supplement: Supplementary file 7 — Supplementary information, Figure S7 [file 41422_2024_989_MOESM7_ESM.pdf]

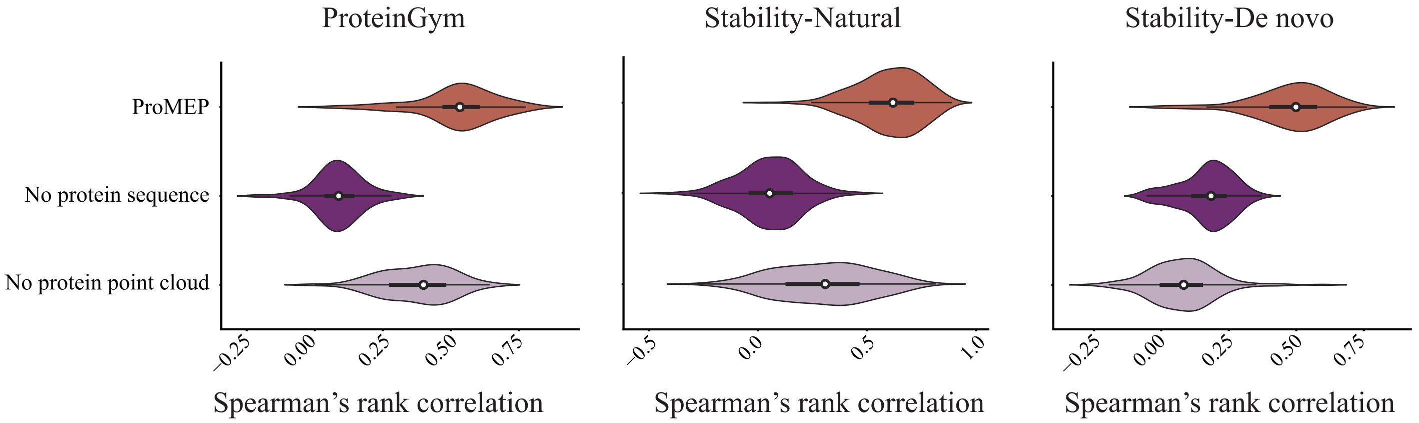

Figure S7 | Ablation study of ProMEP. We evaluate the relative contribution of key model architecture to the final model performance by removing corresponding modules. Three datasets from two benchmarks are used, including the ProteinGym benchmark and the stability benchmark. Specifically, the stability benchmark consists of 776,000 high-quality measured folding stabilities of 331 natural and 148 de novo designed protein domains. For de novo proteins with low sequence similarity to natural proteins, structure context is extremely important to accurately predict the effects of mutations.
