# Supplementary material for: Zero-shot prediction of mutation effects with multimodal deep representation learning guides protein engineering
Source: Cell Res. 2024 Jul 5;34(9):630–47. doi: 10.1038/s41422-024-00989-2 (PMC11369238; doi:10.1038/s41422-024-00989-2)
Supplement: Supplementary file 8 — Supplementary information, Figure S8 [file 41422_2024_989_MOESM8_ESM.pdf]

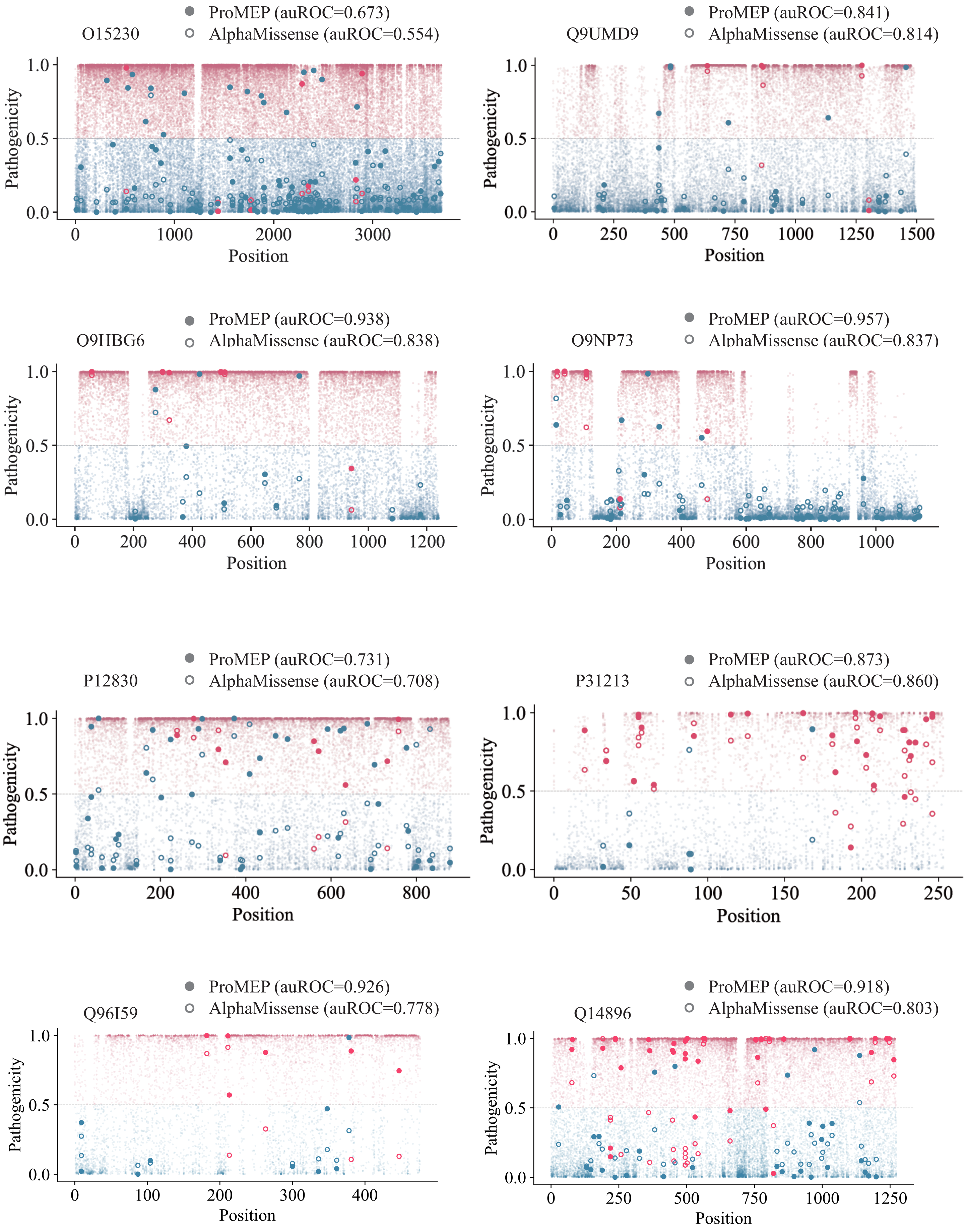

Figure S8 | A comparison of pathogenicity prediction performance on proteins with less than 100 high similarity sequences. Missense variants, depicted as points, are graphically represented against ProMEP pathogenicity scores on the y-axis and amino acid positions on the x-axis. Variants predicted as likely pathogenic are denoted in red, while those predicted as likely benign are indicated in blue. If a variant possesses a clinical label in ClinVar, it is portrayed as a brighter circle. Solid circles signify variants predicted by ProMEP, whereas hollow circles represent variants predicted by AlphaMissense.
