# Supplementary material for: Zero-shot prediction of mutation effects with multimodal deep representation learning guides protein engineering
Source: Cell Res. 2024 Jul 5;34(9):630–47. doi: 10.1038/s41422-024-00989-2 (PMC11369238; doi:10.1038/s41422-024-00989-2)
Supplement: Supplementary file 9 — Supplementary information, Figure S9 [file 41422_2024_989_MOESM9_ESM.pdf]

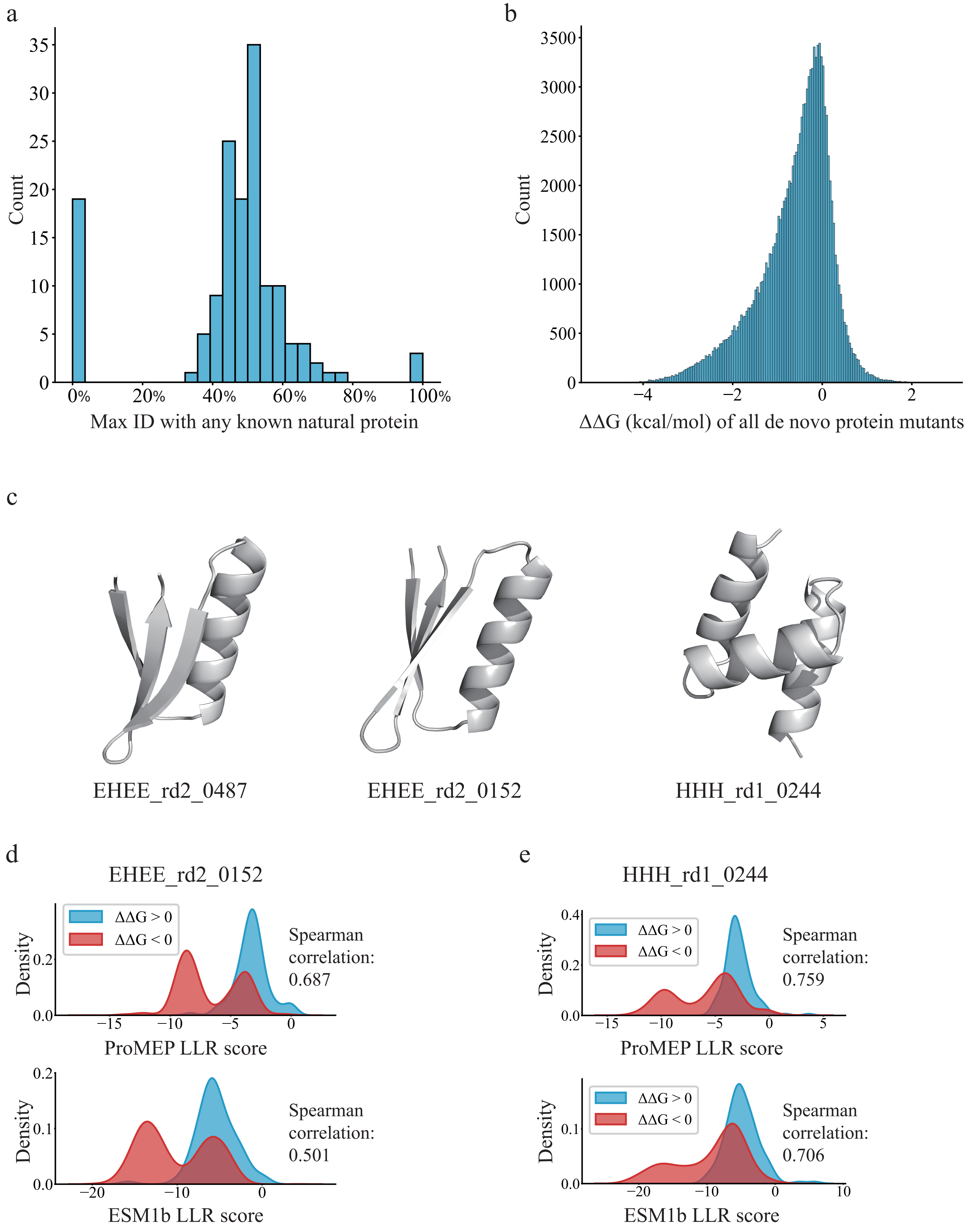

Figure S9 | Analysis of de novo designed proteins in the stability benchmark. a, Distribution of Max ID of all de novo designed proteins. We use Max ID as a measurement of the maximum identity of a de novo designed protein with any available natural protein in the Non-Redundant (NR) protein sequence database. **b,** Distribution of measured changes in protein thermodynamic stability (ΔΔG) of all missense variants on de novo designed proteins. **c,** Predicted structures (via ESMFold) of 3 de novo proteins with max ID < 10%. **d,** The distribution of mutation effects scores of ProMEP and ESM1b across two sets of variants on two additional de novo designed protein domains (EHEE_rd2_0152 and HHH_rd1_0244). Changes in protein thermodynamic stability (ΔΔG) is used to distinguish different class of variants.
