# Supplementary material for: Zero-shot prediction of mutation effects with multimodal deep representation learning guides protein engineering
Source: Cell Res. 2024 Jul 5;34(9):630–47. doi: 10.1038/s41422-024-00989-2 (PMC11369238; doi:10.1038/s41422-024-00989-2)
Supplement: Supplementary file 10 — Supplementary information, Figure S10 [file 41422_2024_989_MOESM10_ESM.pdf]

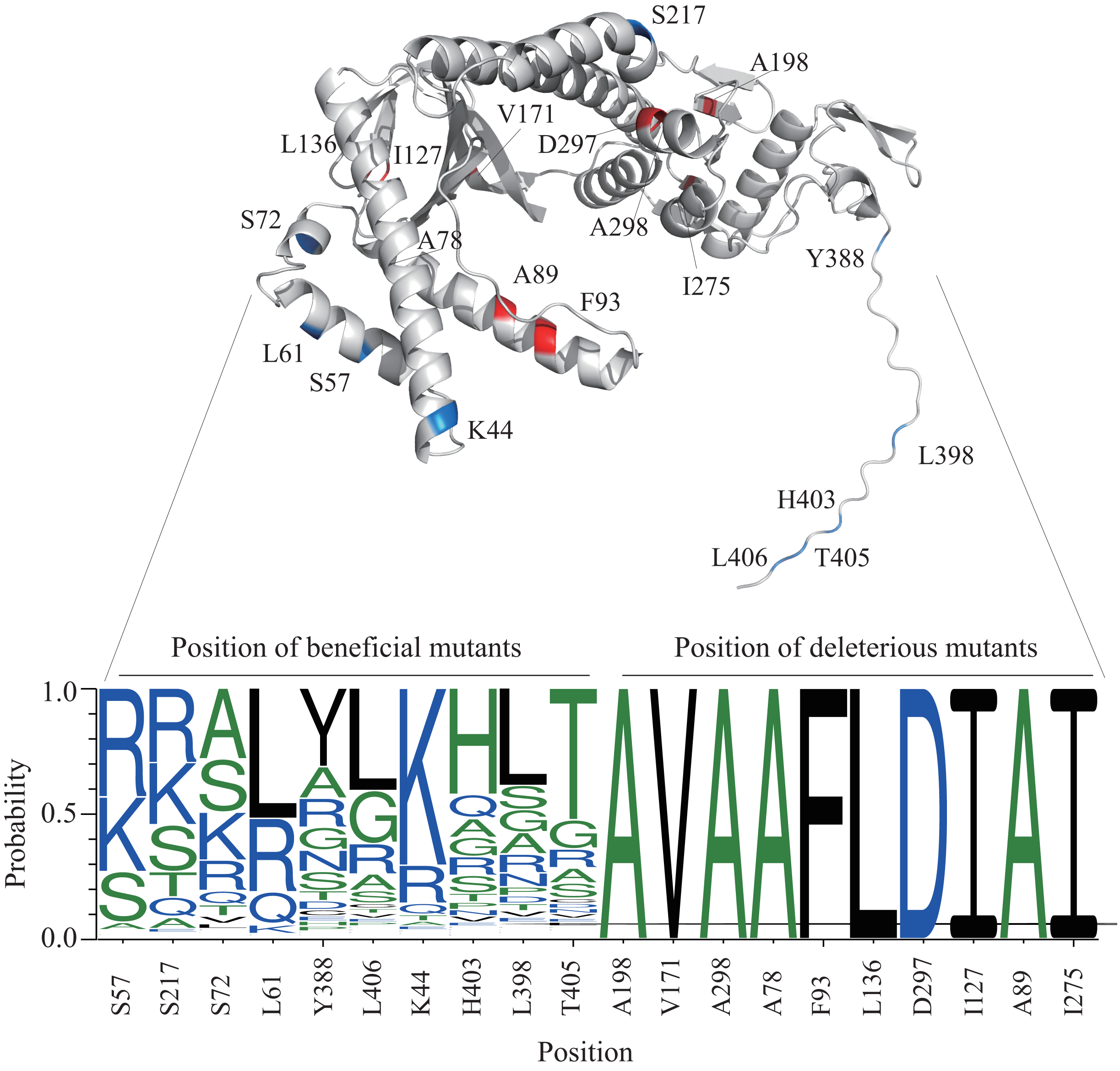

Figure S10 | The probability distributions of all amino acid types at altered positions within TnpB. ProMEP exhibits a high degree of confidence in predicting top-ranked beneficial mutations (e.g., S57R and S217R) as well as all deleterious mutations. For deleterious mutations (e.g., A198R and V171R), the wild-type amino acid predominates the probability distribution.
