# Supplementary material for: Zero-shot prediction of mutation effects with multimodal deep representation learning guides protein engineering
Source: Cell Res. 2024 Jul 5;34(9):630–47. doi: 10.1038/s41422-024-00989-2 (PMC11369238; doi:10.1038/s41422-024-00989-2)
Supplement: Supplementary file 11 — Supplementary information, Figure S11 [file 41422_2024_989_MOESM11_ESM.pdf]

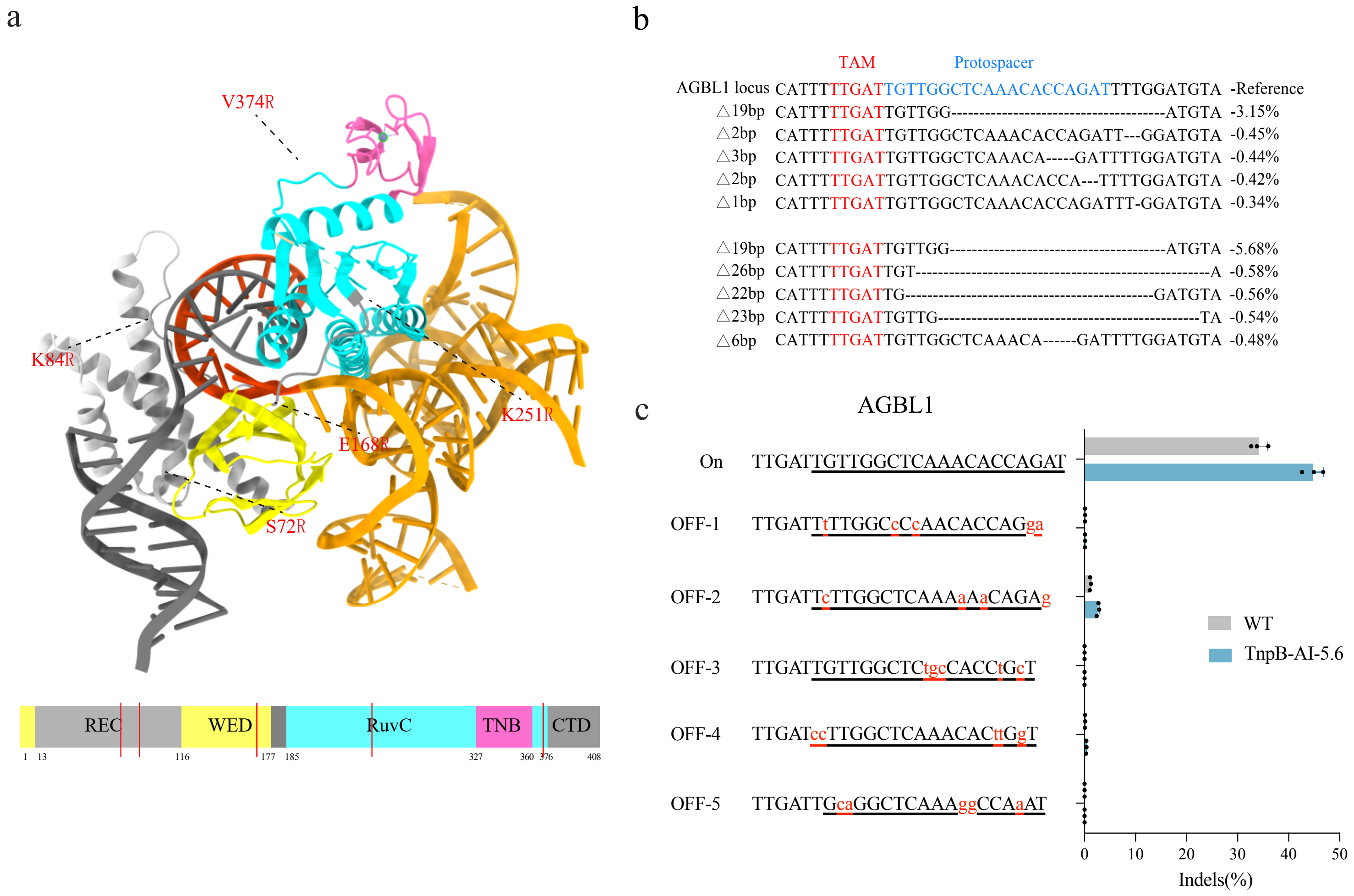

Figure S11 | Protein engineering of TnpB via ProMEP. a, Structures of the TnpB–ωRNA–target DNA complex (PDB:8h1J), amino acid positions obtained by ProMEP are indicated in red. **b,** Distribution of sizes and positions of deletion generated by the TnpB and TnpB-AI-5.6. The TAM and 20-bp target are shown in red and blue, respectively. **c,** Cas9-dependent DNA on- and off-target analysis of the *AGBL1* target by TnpB and TnpB-AI-5.6 in HEK293T cells. Data are mean ± s.d., n = 3 independent biological replicates.
