# Supplementary material for: Zero-shot prediction of mutation effects with multimodal deep representation learning guides protein engineering
Source: Cell Res. 2024 Jul 5;34(9):630–47. doi: 10.1038/s41422-024-00989-2 (PMC11369238; doi:10.1038/s41422-024-00989-2)
Supplement: Supplementary file 12 — Supplementary information, Figure S12 [file 41422_2024_989_MOESM12_ESM.pdf]

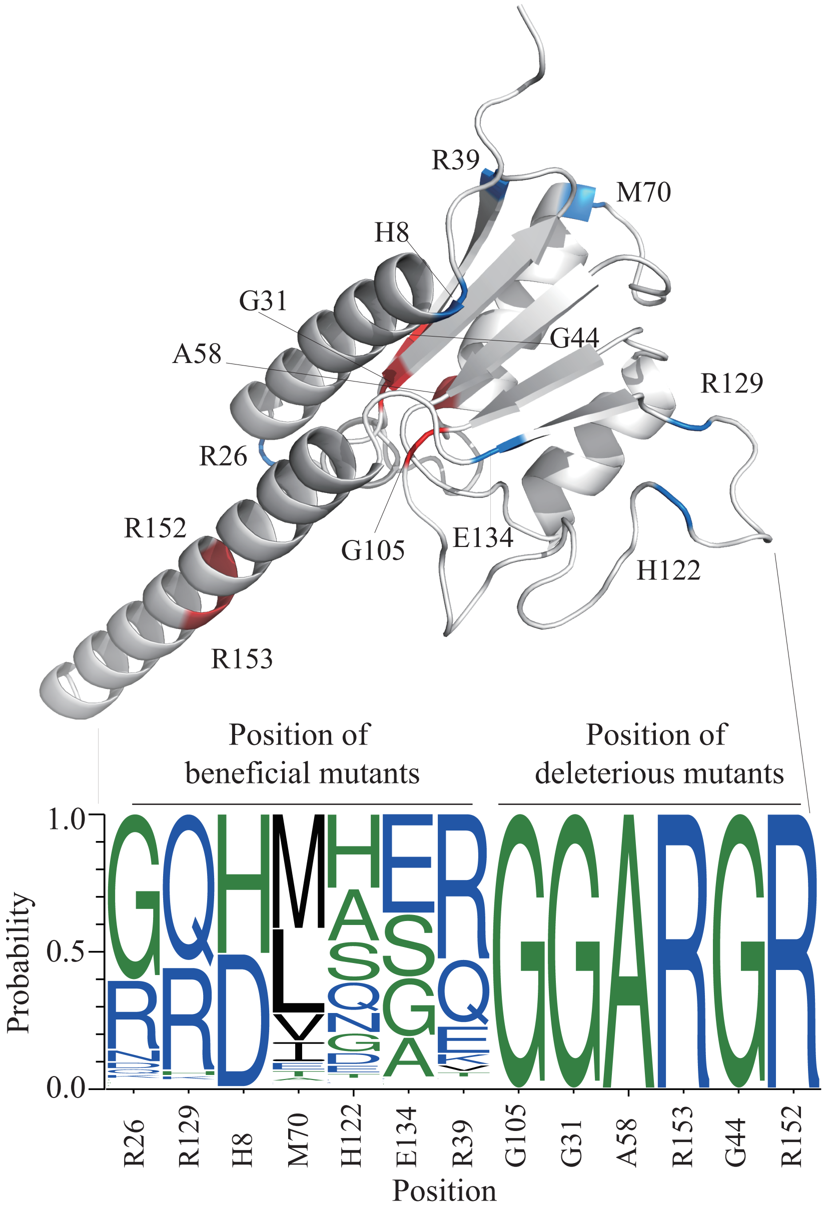

Figure S12 | The probability distributions of all amino acid types at altered positions within TadA. ProMEP demonstrates high confidence in predicting alterations at positions within TadA. Similarly, for deleterious mutations (e.g., G105K and G31L), the wild-type amino acid predominates the probability distribution.
