# Supplementary material for: Zero-shot prediction of mutation effects with multimodal deep representation learning guides protein engineering
Source: Cell Res. 2024 Jul 5;34(9):630–47. doi: 10.1038/s41422-024-00989-2 (PMC11369238; doi:10.1038/s41422-024-00989-2)
Supplement: Supplementary file 13 — Supplementary information, Figure S13 [file 41422_2024_989_MOESM13_ESM.pdf]

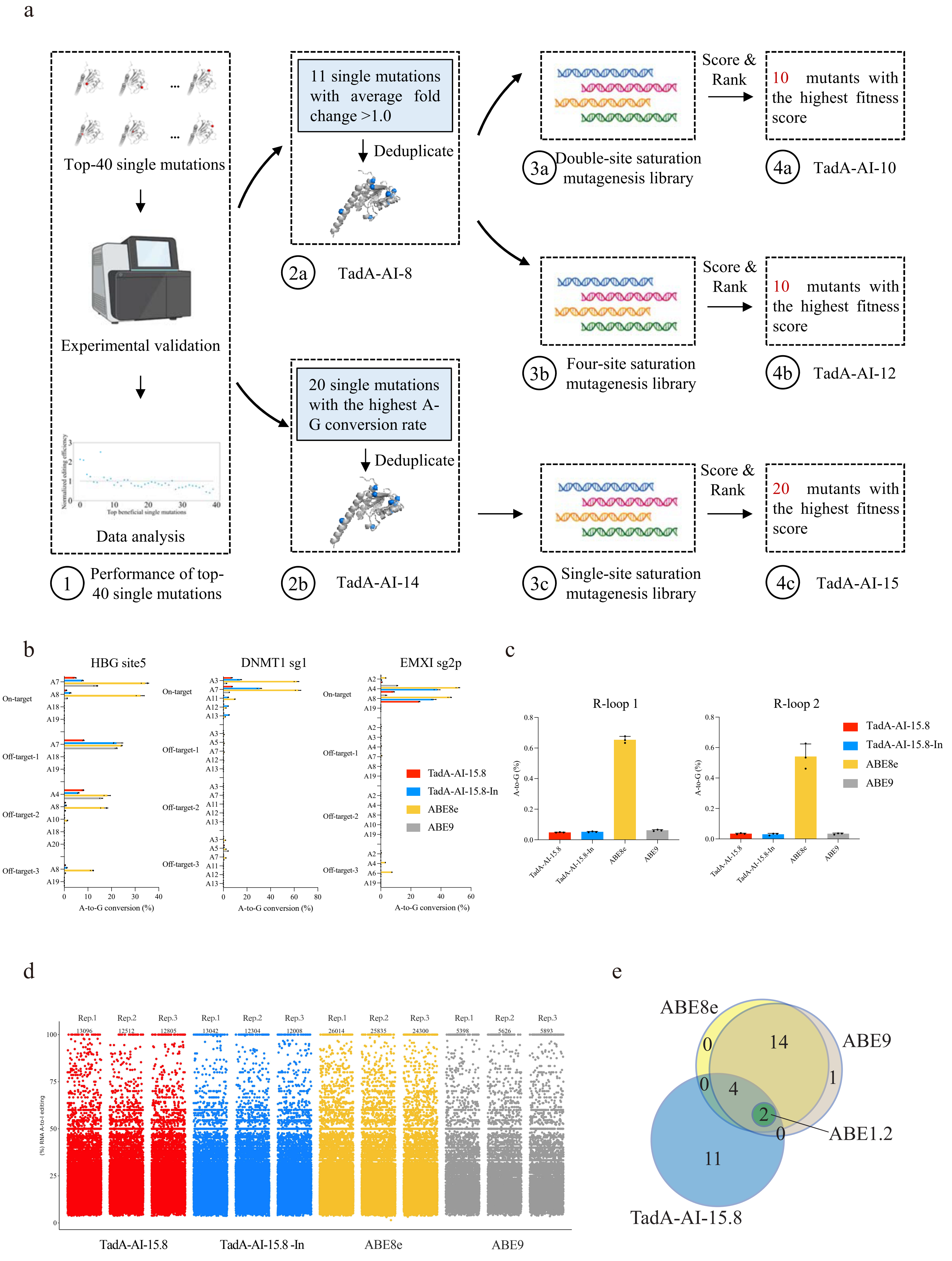
a
Top-40 single mutations
Experimental validation
Data analysis
1 Performance of top-40 single mutations
11 single mutations with average fold change >1.0
Deduplicate
2a TadA-AI-8
20 single mutations with the highest A-G conversion rate
Deduplicate
2b TadA-AI-14
3a Double-site saturation mutagenesis library
3b Four-site saturation mutagenesis library
3c Single-site saturation mutagenesis library
Score & Rank
10 mutants with the highest fitness score
4a TadA-AI-10
10 mutants with the highest fitness score
4b TadA-AI-12
20 mutants with the highest fitness score
4c TadA-AI-15
Normalized editing efficiency
Top beneficial single mutations
b
HBG site5
DNMT1 sg1
EMXI sg2p
On-target
Off-target-1
Off-target-2
Off-target-3
A-to-G conversion (%)
TadA-AI-15.8
TadA-AI-15.8-In
ABE8e
ABE9
c
R-loop 1
R-loop 2
A-to-G (%)
d
Rep.1
Rep.2
Rep.3
13096
12512
12805
13042
12304
12008
26014
25835
24300
5398
5626
5893
(%) RNA A-to-I editing
TadA-AI-15.8
TadA-AI-15.8 -In
ABE8e
ABE9
e
ABE8e
ABE9
ABE1.2
TadA-AI-15.8
14
0
0
4
1
2
0
11

Figure S13 | Protein engineering of TadA via ProMEP. a, ProMEP is employed to guide the screening of TadA mutants with more than 10 mutations. Initially, ProMEP is utilized to identify 40 beneficial single mutants with the highest fitness scores. The editing efficiency of these mutants is then assessed, and subsequently, two variants, namely TadA-AI-8 and TadA-AI-14, are constructed. The TadA-AI-8 variant is created by selecting 11 single mutations with an average fold change greater than 1.0, while ensuring that redundant mutation sites are eliminated during the deduplication process (e.g., for position 134, if both E134G and E134S are mutated, the mutation with higher editing efficiency is retained). Additionally, another strategy is employed to construct the TadA-AI-14 variant, which involved the deduplication of 20 single mutations with the highest A-G conversion rate. Subsequently, three virtual mutagenesis libraries are constructed: a Double-site saturation mutagenesis library (consisting of 4,420,806 mutants) based on TadA-AI-8, a Four-site saturation mutagenesis library (consisting of 24,359,335 mutants) also based on TadA-AI-8, and a Single-site saturation mutagenesis library (consisting of 2,869 mutants) based on TadA-AI-14. The fitness scores for each variant in these virtual libraries are calculated using ProMEP, and subsequently, 40 mutants with the highest fitness scores are selected for further evaluation. **b,** Cas9-dependent DNA on- and off-target analyses of the indicated targets were conducted using TadA-AI-15.8, TadA-AI-15.8-In, ABE8e, and ABE9 in HEK293T cells. Data are mean ± s.d. and n = 3 independent experiments. **c,** Cas9-independent DNA off-target analyses of TadA-AI-15.8, TadA-AI-15.8-In, ABE8e, and ABE9 were performed using the modified orthogonal R-loop assay at each R-loop site with a nSaCas9-sgRNA plasmid. Data are mean ± s.d. of n = 3 independent experiments. **d,** RNA-seq was utilized to evaluate RNA off-target editing activities of TadA-AI-15.8, TadA-AI-15.8-In, ABE8e, and ABE9. Jitter plots from RNA-seq experiments in HEK293T cells demonstrated the efficiencies of A-to-I conversions (y-axis) with TadA-AI-15.8, TadA-AI-15.8-In, ABE8e, and ABE9. Each biological replicate (Rep.) and the total number of modified bases are detailed at the top. **e,** Overlap of mutation sites between ABE1.2, TadA-AI-15.8, ABE8e, and ABE9.
