# Supplementary material for: Zero-shot prediction of mutation effects with multimodal deep representation learning guides protein engineering
Source: Cell Res. 2024 Jul 5;34(9):630–47. doi: 10.1038/s41422-024-00989-2 (PMC11369238; doi:10.1038/s41422-024-00989-2)
Supplement: Supplementary file 14 — Supplementary information, Table S1 [file 41422_2024_989_MOESM14_ESM.pdf]

Table S1 | Performance comparison on function annotation datasets.

Type	Method	EC		EC-384	GO-BP		GO-MF		GO-CC	
		Fmax	AUPR	Accuracy	Fmax	AUPR	Fmax	AUPR	Fmax	AUPR
w/o pretraining	CNN [93]	0.545	0.526	0.517	0.244	0.159	0.354	0.351	0.287	0.204
	ResNet [94]	0.605	0.590	0.241	0.280	0.205	0.405	0.434	0.304	0.214
	LSTM [94]	0.425	0.414	0.110	0.225	0.156	0.321	0.334	0.283	0.192
	Transformer [94]	0.238	0.218	0.266	0.264	0.156	0.211	0.177	0.405	0.210
	GCN [96]	0.320	0.319	0.673	0.252	0.136	0.195	0.147	0.329	0.175
	GAT [97]	0.368	0.320	0.556	0.284	0.171	0.317	0.329	0.385	0.249
	GVP [98]	0.489	0.482	0.655	0.326	0.224	0.426	0.458	0.420	0.279
	GraphQA [99]	0.509	0.543	0.608	0.308	0.199	0.329	0.347	0.413	0.265
Sequence pretraining	UniRep [7]	0.698	0.554	0.733	0.375	0.276	0.505	0.523	0.407	0.3
	ESM [23]	0.864	0.889	0.831	0.452	**0.332**	**0.657**	0.639	0.477	0.324
	ProtTrans [24]	0.838	0.859	0.72.2	0.279	0.188	0.456	0.464	0.408	0.234
Structure pretraining	GearNet [33]	**0.874**	0.892	**0.875**	**0.490**	0.292	0.654	0.596	0.488	0.336
Sequence+Structure pretraining	DeepFRI [34]	0.631	0.547	0.633	0.399	0.282	0.465	0.462	0.460	0.363
	LM-GVP [95]	0.664	0.710	-	0.417	0.302	0.515	0.580	**0.527**	**0.423**
	Ours	**0.882**	**0.908**	**0.901**	**0.521**	0.299	**0.660**	**0.656**	**0.568**	0.410
