# Supplementary material for: Zero-shot prediction of mutation effects with multimodal deep representation learning guides protein engineering
Source: Cell Res. 2024 Jul 5;34(9):630–47. doi: 10.1038/s41422-024-00989-2 (PMC11369238; doi:10.1038/s41422-024-00989-2)
Supplement: Supplementary file 15 — Supplementary information, Table S2 [file 41422_2024_989_MOESM15_ESM.pdf]

Table S2 | Performance comparison on PPI datasets.

Type	Method	PPI-Mouse		PPI-Fly		PPI-Ecoli	
		F1	AUPR	F1	AUPR	F1	AUPR
w/o pretraining	CNN [93]	0.418	0.526	0.294	0.317	0.435	0.457
	ResNet [94]	0.361	0.515	0.317	0.434	0.473	0.507
	LSTM [94]	0.467	0.549	0.417	0.431	0.787	0.904
	Transformer [94]	0.503	0.543	0.377	0.388	0.365	0.401
Sequence pretraining	UniRep [7]	0.498	0.566	0.583	0.620	0.514	0.584
	ESM [23]	0.580	**0.686**	0.692	0.764	**0.597**	**0.695**
	ProtTrans [24]	**0.605**	0.685	**0.711**	**0.786**	0.590	0.679
Sequence+Structure pretraining	Ours	**0.620**	**0.707**	**0.720**	**0.790**	**0.629**	0.667
