# Supplementary material for: Zero-shot prediction of mutation effects with multimodal deep representation learning guides protein engineering
Source: Cell Res. 2024 Jul 5;34(9):630–47. doi: 10.1038/s41422-024-00989-2 (PMC11369238; doi:10.1038/s41422-024-00989-2)
Supplement: Supplementary file 16 — Supplementary information, Table S3 [file 41422_2024_989_MOESM16_ESM.pdf]

Table S3 | Description of 15 function-related datasets.

Task	Dataset	Train	Valid	Test	Number of classes	Classifier	Size of representation of each protein
EC	PDB	15,550	1,729	1,919	538	MLP	1,280
	New-392	227,362	-	392	5,242	CLEAN	1,280
	Price-149	227.362	-	149	5,242	CLEAN	1,280
	Reaction	29,215	2,562	5,651	384	MLP	1,280
GO	BP	29,898	3,322	3,415	1,943	MLP	1,280
	CC	29,898	3,322	3,415	320	MLP	1,280
	MF	29,898	3,322	3,415	489	MLP	1,280
PPI (Cross-species)	Fly	-	-	55,000	2	MLP	1,280
	Mouse	-	-	55,000	2	MLP	1,280
	Ecoli	-	-	22,000	2	MLP	1,280
	Human	421,746	52,725	-	2	MLP	1280
PPI (Virus-human)	Denovo	9,754	-	850	2	MLP	1,280
	EBOLA	22,682	-	300	2	MLP	1,280
	H1N1	21,616	-	762	2	MLP	1,280
PPI (Multi-class)	SHS148K	35,585	-	8,903	7	GNN	L * 1280
	STRING	473,112	-	120,287	7	GNN	L * 1280
