# Supplementary material for: Zero-shot prediction of mutation effects with multimodal deep representation learning guides protein engineering
Source: Cell Res. 2024 Jul 5;34(9):630–47. doi: 10.1038/s41422-024-00989-2 (PMC11369238; doi:10.1038/s41422-024-00989-2)
Supplement: Supplementary file 17 — Supplementary information, Table S4 [file 41422_2024_989_MOESM17_ESM.pdf]

Table S4 | Number of proteins in the test set with 30%, 40%, 50%, 70% and 95% sequence identity to the training set.

Dataset	30%	40%	50%	70%	95%
EC-PDB	720	902	1,117	1,476	1,919
GO-MF GO-BP GO-CC	1,717	1,937	2,199	2,733	3,416
