# Supplementary material for: Zero-shot prediction of mutation effects with multimodal deep representation learning guides protein engineering
Source: Cell Res. 2024 Jul 5;34(9):630–47. doi: 10.1038/s41422-024-00989-2 (PMC11369238; doi:10.1038/s41422-024-00989-2)
Supplement: Supplementary file 18 — Supplementary information, Table S5 [file 41422_2024_989_MOESM18_ESM.pdf]

Table S5 | Number of proteins in the test set with 30%, 40%, 50%, 70% and 95% structure identity to the training set.

Dataset	30%	40%	50%	70%	95%
EC-PDB	117	369	647	1056	1539
GO-MF<br>GO-BP<br>GO-CC	30	185	738	2,045	2,933
