# Supplementary material for: Zero-shot prediction of mutation effects with multimodal deep representation learning guides protein engineering
Source: Cell Res. 2024 Jul 5;34(9):630–47. doi: 10.1038/s41422-024-00989-2 (PMC11369238; doi:10.1038/s41422-024-00989-2)
Supplement: Supplementary file 19 — Supplementary information, Table S6 [file 41422_2024_989_MOESM19_ESM.pdf]

Table S6 | Number of proteins in virus-human datasets.

Dataset	Human proteins	Virus proteins
Denovo	2,341	445
EBOLA	7,816	659
H1N1	6,636	641
