# Supplementary material for: Zero-shot prediction of mutation effects with multimodal deep representation learning guides protein engineering
Source: Cell Res. 2024 Jul 5;34(9):630–47. doi: 10.1038/s41422-024-00989-2 (PMC11369238; doi:10.1038/s41422-024-00989-2)
Supplement: Supplementary file 20 — Supplementary information, Table S7 [file 41422_2024_989_MOESM20_ESM.pdf]

Table S7 | Performance metrics used in this study.

Benchmark	Method	How performance metrics were obtained
EC/ EC-384/ GO-BP/ GO-MF/ GO-CC	CNN	Cited from Zhang et al., Protein Representation Learning by Geometric Structure Pretraining.
	ResNet	Cited from Zhang et al., Protein Representation Learning by Geometric Structure Pretraining.
	LSTM	Cited from Zhang et al., Protein Representation Learning by Geometric Structure Pretraining.
	Transformer	Cited from Zhang et al., Protein Representation Learning by Geometric Structure Pretraining.
	GCN	Cited from Zhang et al., Protein Representation Learning by Geometric Structure Pretraining.
	GAT	Cited from Zhang et al., Protein Representation Learning by Geometric Structure Pretraining.
	GVP	Cited from Zhang et al., Protein Representation Learning by Geometric Structure Pretraining.
	GraphQA	Cited from Zhang et al., Protein Representation Learning by Geometric Structure Pretraining.
	UniRep	Obtained by ourselves
	ESM	Cited from Zhang et al., Protein Representation Learning by Geometric Structure Pretraining.
	ProtTrans	Obtained by ourselves
	GearNet	Cited from Zhang et al., Protein Representation Learning by Geometric Structure Pretraining.
	DeepFRI	Cited from Zhang et al., Protein Representation Learning by Geometric Structure Pretraining.
	LM-GVP	Cited from Zhang et al., Protein Representation Learning by Geometric Structure Pretraining.
	Ours	Obtained by ourselves
EC-New-392/ EC-Price-149	Ours-CLEAN	Obtained by ourselves
	CLEAN	Cited from Yu et al., Enzyme function prediction using contrastive learning.
	ProtInfer	Cited from Yu et al., Enzyme function prediction using contrastive learning.
	DeepEC	Cited from Yu et al., Enzyme function prediction using contrastive learning.
	ECPred	Cited from Yu et al., Enzyme function prediction using contrastive learning.
PPI-Mouse/ PPI-Fly/ PPI-Ecoli	CNN	Obtained by ourselves
	ResNet	Obtained by ourselves
	LSTM	Obtained by ourselves
	Transformer	Obtained by ourselves
	UniRep	Obtained by ourselves
	ESM	Obtained by ourselves
	ProtTrans	Obtained by ourselves
	Ours	Obtained by ourselves
PPI-Denovo/PPI-EBOLA/ PPI-H1NI	Ours	Obtained by ourselves
	MTT	Cited from Dong et al., A multitask transfer learning framework for the prediction of virus-human protein-protein interactions.
	Denovo	Cited from Dong et al., A multitask transfer learning framework for the prediction of virus-human protein-protein interactions.
	Generalized	Cited from Dong et al., A multitask transfer learning framework for the prediction of virus-human protein-protein interactions.
PPI-SHS148K/PPI-STRING	Ours	Obtained by ourselves
	GNN-PPI	Cited from Lv et al., Learning Unknown from Correlations: Graph Neural Network for Inter-novel-protein Interaction Prediction
	OntoProtein	Cited from Lv et al., Learning Unknown from Correlations: Graph Neural Network for Inter-novel-protein Interaction Prediction
	PIPR	Cited from Lv et al., Learning Unknown from Correlations: Graph Neural Network for Inter-novel-protein Interaction Prediction

	ProtBert	Cited from Lv et al., Learning Unknown from Correlations: Graph Neural Network for Inter-novel-protein Interaction Prediction
	Multi-modal	Cited from Lv et al., Learning Unknown from Correlations: Graph Neural Network for Inter-novel-protein Interaction Prediction
ProteinGym	ProMEP	Obtained by ourselves
	AlphaMissense	Retrieve predicted scores from AlphaMissense and calculated by ourselves
	ESM2_650M	Obtained by ourselves
	ESM2_3B	Obtained by ourselves
	ProstT5	Obtained by ourselves
	GEMME	Cited from Notin et al., Tranception: protein fitness prediction with autoregressive transformers and inference-time retrieval
	Tranception	Cited from Notin et al., Tranception: protein fitness prediction with autoregressive transformers and inference-time retrieval
	EVE	Cited from Notin et al., Tranception: protein fitness prediction with autoregressive transformers and inference-time retrieval
	MSA	Cited from Notin et al., Tranception: protein fitness prediction with autoregressive transformers and inference-time retrieval
	ESM1v	Cited from Notin et al., Tranception: protein fitness prediction with autoregressive transformers and inference-time retrieval
	DeepSequence	Cited from Notin et al., Tranception: protein fitness prediction with autoregressive transformers and inference-time retrieval
	EVmutation	Cited from Notin et al., Tranception: protein fitness prediction with autoregressive transformers and inference-time retrieval
	RITA	Cited from Notin et al., Tranception: protein fitness prediction with autoregressive transformers and inference-time retrieval
	Unirep	Cited from Notin et al., Tranception: protein fitness prediction with autoregressive transformers and inference-time retrieval
	ESM1b	Cited from Notin et al., Tranception: protein fitness prediction with autoregressive transformers and inference-time retrieval
	ProtGPT2	Cited from Notin et al., Tranception: protein fitness prediction with autoregressive transformers and inference-time retrieval
Pathogenicity-de novo variants	ProMEP	Obtained by ourselves
	AlphaMissense	Cited from Cheng et al., Accurate proteome-wide missense variant effect prediction with AlphaMissense.
	ESM2_650M	Obtained by ourselves
	ESM2_3B	Obtained by ourselves
	ProstT5	Obtained by ourselves
	ESM1b	Obtained by ourselves
	ESM1v	Obtained by ourselves
	Tranception	Obtained by ourselves
Stability-Natural /Stability-De novo	ProMEP<br>ESM2_650M	Obtained by ourselves<br>Obtained by ourselves
	ESM2_3B	Obtained by ourselves
	ProstT5	Obtained by ourselves
	ESM1b	Obtained by ourselves
	ESM1v	Obtained by ourselves
	Tranception	Obtained by ourselves
	GEMME	Obtained by ourselves
Swiss-Prot-1325	ProMEP	Obtained by ourselves
	DeepFRI	Obtained by ourselves
	HEAL	Obtained by ourselves
	Random	Obtained by ourselves
CASP12/ TS115/ CB513	ProMEP<br>ESM1b	Obtained by ourselves<br>Obtained by ourselves
	Alignment	Cited from Rao et al., Evaluating Protein Transfer Learning with TAPE
	ResNet	Cited from Rao et al., Evaluating Protein Transfer Learning with TAPE
	UniRep	Cited from Rao et al., Evaluating Protein Transfer Learning with TAPE
	Transformer	Cited from Rao et al., Evaluating Protein Transfer Learning with TAPE

	LSTM	Cited from Rao et al., Evaluating Protein Transfer Learning with TAPE
SCOP-100/ CATH-100	ProMEP	Obtained by ourselves
	ESM1b	Obtained by ourselves
	ProtTrans	Obtained by ourselves
	UniRep	Obtained by ourselves
SCOPe v2.07	ProMEP	Obtained by ourselves
	GraSR	Cited from Xia et al., Fast protein structure comparison through effective representation learning with contrastive graph neural networks.
	DeepFold	Cited from Xia et al., Fast protein structure comparison through effective representation learning with contrastive graph neural networks.
	SGM	Cited from Xia et al., Fast protein structure comparison through effective representation learning with contrastive graph neural networks.
	SSEM	Cited from Xia et al., Fast protein structure comparison through effective representation learning with contrastive graph neural networks.
