# Supplementary material for: Zero-shot prediction of mutation effects with multimodal deep representation learning guides protein engineering
Source: Cell Res. 2024 Jul 5;34(9):630–47. doi: 10.1038/s41422-024-00989-2 (PMC11369238; doi:10.1038/s41422-024-00989-2)
Supplement: Supplementary file 21 — Supplementary information, Table S8 [file 41422_2024_989_MOESM21_ESM.pdf]

Table S8 | Fold class of 13,265 domains from the SCOPe database.

Fold	Number of proteins
a : All alpha proteins	2286
b : All beta proteins)	2757
c : Alpha and beta proteins (a/b)	4148
d : Alpha and beta proteins (a+b)	3378
e : Multi-domain proteins (alpha and beta)	279
f : Membrane and cell surface proteins and peptides	213
g : Small proteins	204
