# Supplementary material for: Zero-shot prediction of mutation effects with multimodal deep representation learning guides protein engineering
Source: Cell Res. 2024 Jul 5;34(9):630–47. doi: 10.1038/s41422-024-00989-2 (PMC11369238; doi:10.1038/s41422-024-00989-2)
Supplement: Supplementary file 22 — Supplementary information, Data S1 [file 41422_2024_989_MOESM22_ESM.pdf]

Data S1 | Probability of all types of amino acids for the altered positions in TnpB.

Position	A	G	V	L	I	S	T	C	M	D
S57	0.032158	0.0013066	0.0007346	0.0002806	0.0001898	0.1998542	0.0108124	5.61E-05	0.0001189	0.0001621
S217	0.0434991	0.0014824	0.0040376	0.0063269	0.0012203	0.1862501	0.0975606	0.0001294	0.000253	0.0030609
S72	0.2777464	0.0095248	0.0280905	0.021486	0.003515	0.2075054	0.043322	0.0004532	0.0021909	0.0016783
L61	0.0124579	0.0006434	0.0024448	0.512996	0.0033521	0.0020314	0.0024008	0.0001618	0.0004201	4.26E-05
Y388	0.1232047	0.0884171	0.0107882	0.0047071	0.0024158	0.0745146	0.0379007	0.0241428	0.0007731	0.0365198
L406	0.0658833	0.2086308	0.0204491	0.4074456	0.0050157	0.04842	0.0221376	0.0215214	0.00073	0.0105192
K44	0.020946	0.0030037	0.0024528	0.0042929	0.0007795	0.0102534	0.0261447	0.0003006	0.0002132	0.001555
H403	0.077097	0.0741175	0.0232805	0.0111124	0.0054742	0.069193	0.0342066	0.0092384	0.0007631	0.0098618
L398	0.0840522	0.0902175	0.0272396	0.3836856	0.0097271	0.0971585	0.0315892	0.0173317	0.0014439	0.030288
T405	0.0617812	0.1157233	0.0216101	0.0141229	0.0045762	0.0577804	0.5198766	0.0193784	0.0008259	0.0190759
A198	0.9999338	1.57E-05	1.78E-05	4.31E-06	3.50E-07	1.76E-05	6.48E-06	2.59E-06	4.05E-07	5.24E-08
V171	0.0003069	7.61E-07	0.9938625	0.0002843	0.0054999	4.25E-07	4.82E-06	9.01E-06	5.24E-06	5.30E-09
A298	0.9997502	4.61E-06	0.0001829	3.48E-07	1.43E-06	5.15E-05	2.96E-06	3.43E-06	6.79E-07	1.43E-08
A78	0.9996139	5.76E-06	8.87E-06	8.46E-08	6.52E-07	0.0003451	1.77E-05	6.39E-06	1.44E-07	8.45E-08
F93	2.98E-06	2.74E-07	1.27E-06	3.94E-05	1.39E-07	4.01E-06	6.78E-07	9.16E-06	2.89E-07	1.35E-08
L136	6.62E-06	6.32E-08	0.0001728	0.9995796	0.000117	5.83E-07	1.60E-06	1.52E-06	7.98E-06	4.31E-09
D297	2.63E-06	2.54E-05	2.02E-08	9.12E-09	4.24E-09	2.79E-06	6.86E-08	1.28E-07	1.73E-08	0.9999372
I127	5.15E-06	3.39E-08	0.0003066	2.12E-05	0.9996525	2.34E-08	1.09E-06	1.38E-07	7.65E-06	2.04E-10
A89	0.9998046	4.08E-06	2.48E-06	7.70E-09	2.72E-08	0.0001755	1.16E-05	2.57E-07	4.76E-09	4.07E-07
I275	9.78E-07	9.82E-08	0.0003465	3.52E-05	0.9995982	3.33E-07	7.99E-06	2.11E-07	6.08E-06	7.29E-08

Position	E	N	Q	K	F	Y	W	P	H	R
S57	0.0001052	0.0029366	0.0169109	0.2945923	3.91E-05	9.18E-05	7.94E-06	3.78E-05	0.0027871	0.4368179
S217	0.013082	0.0044001	0.0822831	0.2440734	0.000394	0.0017584	0.0004219	0.0002311	0.0047041	0.3048316
S72	0.0117059	0.0037788	0.054656	0.191143	0.0022782	0.0023513	0.0028962	0.0020748	0.0028425	0.1307606
L61	0.0002063	0.000653	0.1319554	0.0354259	0.0001616	6.12E-05	4.28E-05	4.91E-05	0.0031952	0.2912985
Y388	0.0179506	0.0843745	0.0074248	0.0107391	0.0012566	0.3116401	0.0016728	0.0181306	0.0209261	0.1225006
L406	0.0165588	0.0122359	0.0098367	0.0032708	0.0021529	0.002435	0.0004726	0.0133175	0.0076935	0.1212733
K44	0.0139864	0.0047156	0.0435375	0.7004274	0.0004574	0.0001852	8.62E-05	0.0003248	0.0087078	0.1576301
H403	0.0146517	0.0268936	0.095048	0.0099027	0.0022772	0.0031741	0.0001581	0.0299482	0.4198074	0.0837944
L398	0.0179397	0.0552193	0.0106739	0.0109879	0.0043593	0.0050396	0.0005657	0.0370076	0.0107217	0.0747505
T405	0.0174051	0.019192	0.0094998	0.0045701	0.0037467	0.003707	0.0007298	0.0104612	0.0091157	0.0868217
A198	2.07E-07	4.21E-08	1.35E-07	1.03E-08	2.35E-07	1.51E-07	3.70E-08	3.84E-09	1.06E-07	8.61E-08
V171	8.26E-08	2.97E-09	4.24E-08	7.46E-09	2.46E-05	1.04E-06	1.90E-07	1.01E-07	9.57E-09	7.64E-08
A298	2.16E-08	1.76E-06	8.78E-08	1.31E-08	5.70E-08	4.86E-08	2.23E-08	1.37E-08	5.03E-08	7.66E-08
A78	4.07E-08	6.48E-08	1.36E-08	9.14E-09	1.61E-07	3.31E-08	5.50E-08	7.69E-07	2.45E-08	7.33E-08
F93	1.03E-08	1.05E-07	1.97E-08	3.40E-09	0.9998674	7.19E-05	1.86E-06	3.35E-11	3.41E-07	7.20E-08
L136	7.88E-09	5.15E-08	2.69E-07	3.31E-08	0.0001094	4.92E-07	3.65E-07	8.35E-07	5.82E-07	7.10E-08
D297	1.67E-05	1.42E-05	1.86E-07	3.19E-08	1.29E-08	9.97E-08	1.15E-08	6.12E-11	4.08E-07	5.59E-08
I127	3.88E-10	1.16E-07	3.55E-09	4.20E-08	5.02E-06	3.76E-07	2.56E-08	2.92E-08	6.32E-09	4.35E-08
A89	2.69E-07	7.94E-07	6.38E-08	1.12E-08	6.30E-08	8.97E-09	1.03E-09	2.35E-11	3.03E-08	3.03E-08
I275	5.03E-08	4.19E-07	1.90E-08	4.14E-08	2.60E-06	1.34E-07	3.57E-08	1.02E-06	6.42E-09	2.43E-08
