# Supplementary material for: Zero-shot prediction of mutation effects with multimodal deep representation learning guides protein engineering
Source: Cell Res. 2024 Jul 5;34(9):630–47. doi: 10.1038/s41422-024-00989-2 (PMC11369238; doi:10.1038/s41422-024-00989-2)
Supplement: Supplementary file 23 — Supplementary information, Data S2 [file 41422_2024_989_MOESM23_ESM.pdf]

# Data S2 | Probability of all types of amino acids for the altered positions in TadA.

| Position | A | G | V | L | I | S | T | C | M | D |
|---|---|---|---|---|---|---|---|---|---|---|
| R26 | 1.01E-02 | 5.98E-01 | 1.38E-04 | 1.58E-03 | 3.06E-05 | 9.45E-03 | 8.38E-04 | 2.30E-03 | 7.69E-04 | 2.32E-02 |
| R129 | 2.74E-03 | 5.78E-04 | 1.05E-03 | 1.60E-03 | 3.81E-04 | 4.70E-03 | 3.20E-03 | 9.63E-04 | 1.90E-03 | 7.31E-04 |
| H8 | 5.11E-04 | 6.94E-05 | 1.04E-04 | 2.30E-05 | 4.41E-05 | 5.22E-04 | 2.35E-04 | 1.57E-04 | 1.01E-05 | 4.78E-01 |
| M70 | 1.25E-02 | 7.22E-05 | 1.02E-01 | 3.07E-01 | 7.49E-02 | 3.04E-03 | 2.68E-02 | 5.04E-03 | 4.15E-01 | 2.36E-04 |
| H122 | 1.91E-01 | 7.06E-02 | 2.04E-03 | 7.08E-03 | 6.20E-04 | 1.44E-01 | 2.04E-02 | 5.92E-03 | 3.09E-03 | 4.67E-02 |
| E134 | 1.45E-01 | 2.16E-01 | 1.10E-03 | 9.98E-05 | 1.25E-04 | 2.31E-01 | 8.25E-03 | 2.65E-03 | 4.36E-04 | 9.33E-03 |
| R39 | 6.22E-03 | 2.49E-04 | 2.59E-02 | 2.50E-03 | 7.82E-03 | 2.76E-03 | 2.14E-02 | 1.25E-03 | 2.78E-03 | 2.16E-03 |
| G105 | 1.46E-04 | 1.00E+00 | 6.64E-09 | 1.35E-09 | 4.01E-10 | 5.56E-06 | 4.65E-09 | 4.33E-07 | 4.47E-09 | 3.07E-08 |
| G31 | 4.45E-06 | 1.00E+00 | 5.28E-09 | 3.33E-10 | 1.09E-10 | 2.07E-06 | 9.01E-09 | 1.77E-07 | 5.94E-10 | 8.72E-09 |
| A58 | 1.00E+00 | 1.11E-04 | 8.42E-07 | 1.07E-08 | 8.85E-09 | 5.04E-05 | 2.04E-06 | 1.40E-06 | 3.64E-08 | 7.39E-09 |
| R153 | 1.30E-07 | 4.27E-07 | 3.04E-09 | 2.08E-08 | 2.93E-09 | 1.08E-06 | 6.01E-08 | 1.59E-07 | 6.56E-08 | 2.03E-08 |
| G44 | 2.61E-05 | 1.00E+00 | 5.34E-09 | 7.81E-10 | 1.72E-10 | 5.92E-06 | 4.96E-08 | 4.02E-07 | 4.61E-09 | 3.63E-07 |
| R152 | 1.40E-08 | 1.47E-08 | 2.44E-08 | 2.98E-07 | 3.91E-08 | 4.38E-08 | 1.49E-08 | 1.91E-07 | 2.43E-08 | 1.41E-10 |
| | | | | | | | | | | |
| Position | E | N | Q | K | F | Y | W | P | H | R |
| R26 | 6.05E-03 | 4.49E-02 | 1.91E-02 | 1.40E-02 | 6.38E-04 | 7.23E-04 | 4.44E-04 | 1.79E-06 | 8.81E-03 | 2.59E-01 |
| R129 | 3.32E-03 | 2.92E-03 | 5.57E-01 | 1.70E-02 | 7.44E-04 | 4.19E-03 | 2.78E-04 | 6.47E-04 | 2.17E-02 | 3.74E-01 |
| H8 | 2.60E-03 | 1.91E-03 | 3.46E-04 | 7.18E-06 | 2.71E-05 | 1.36E-04 | 3.81E-06 | 2.07E-04 | 5.15E-01 | 5.93E-06 |
| M70 | 3.23E-02 | 4.90E-04 | 1.48E-02 | 1.19E-03 | 1.02E-03 | 8.82E-04 | 3.14E-04 | 2.23E-05 | 1.52E-03 | 1.15E-03 |
| H122 | 2.78E-02 | 7.62E-02 | 1.10E-01 | 9.14E-03 | 1.06E-03 | 2.24E-03 | 2.58E-04 | 1.03E-04 | 2.70E-01 | 1.28E-02 |
| E134 | 3.59E-01 | 5.04E-03 | 1.05E-02 | 1.02E-03 | 3.66E-04 | 7.50E-04 | 1.97E-04 | 4.42E-03 | 4.11E-03 | 1.09E-03 |
| R39 | 9.88E-02 | 4.35E-03 | 2.40E-01 | 3.98E-02 | 5.22E-05 | 9.36E-05 | 2.34E-05 | 5.97E-07 | 3.65E-03 | 5.40E-01 |
| G105 | 4.23E-09 | 6.81E-09 | 1.05E-09 | 3.51E-10 | 4.08E-09 | 1.42E-09 | 8.67E-09 | 2.31E-09 | 7.31E-10 | 3.21E-09 |
| G31 | 2.53E-09 | 5.05E-09 | 8.73E-10 | 1.77E-10 | 1.02E-09 | 8.43E-10 | 5.26E-09 | 5.10E-10 | 8.26E-10 | 7.59E-09 |
| A58 | 5.10E-09 | 7.54E-09 | 1.71E-09 | 1.07E-10 | 4.00E-09 | 1.03E-09 | 3.04E-10 | 1.02E-05 | 1.02E-09 | 4.62E-10 |
| R153 | 5.30E-08 | 1.07E-06 | 2.28E-06 | 2.89E-05 | 5.05E-08 | 9.52E-07 | 4.12E-07 | 2.57E-10 | 2.38E-05 | 1.00E+00 |
| G44 | 3.37E-08 | 1.20E-07 | 1.20E-08 | 7.89E-09 | 7.19E-09 | 2.92E-09 | 2.92E-08 | 1.77E-09 | 9.93E-09 | 2.48E-08 |
| R152 | 4.02E-09 | 5.23E-09 | 2.45E-07 | 2.79E-06 | 2.11E-09 | 4.01E-09 | 4.98E-08 | 5.43E-11 | 1.12E-07 | 1.00E+00 |
