# Supplementary material for: Zero-shot prediction of mutation effects with multimodal deep representation learning guides protein engineering
Source: Cell Res. 2024 Jul 5;34(9):630–47. doi: 10.1038/s41422-024-00989-2 (PMC11369238; doi:10.1038/s41422-024-00989-2)
Supplement: Supplementary file 24 — Supplementary information, Data S3 [file 41422_2024_989_MOESM24_ESM.pdf]

Data S3 | Editing efficiency of 40 beneficial mutations in TadA.

PD1 sg4 :CTTCCA6CATGAGCGTGGTCA						
TadA mutation	A Positio	A6-to-G6(%)				single-site mutant mean/ABE1.2
		Rep1	Rep2	Rep3	mean	
R26G	A6	3.01189701	2.91213136	3.23280948	3.05227928	0.949297989
R129Q	A6	1.87265299	1.91752318	1.79444171	1.8615393	0.578962588
H8D	A6	3.09823966	3.56432823	3.47357457	3.37871416	1.05082342
M70L	A6	3.32252214	3.6871312	3.21946746	3.40970693	1.060462571
H122A	A6	2.06191029	2.38827756	2.45818257	2.30279014	0.716197256
E134S	A6	6.83443696	6.69358613	6.94931874	6.82578061	2.122905282
E134G	A6	8.04735099	7.80952989	8.33789811	8.06492633	2.508295488
H122S	A6	2.3903336	2.57830863	2.70234351	2.55699524	0.795258304
R39Q	A6	2.99274969	2.59641511	3.00882685	2.86599722	0.891361879
H122Q	A6	3.95508026	3.70623112	3.78414491	3.8151521	1.186561216
E134A	A6	5.85142974	7.36696092	6.89055764	6.70298277	2.084713578
H36L	A6	2.66287955	2.88925285	2.93832393	2.83015211	0.880213592
I156K	A6	2.27944131	2.61688981	2.75488948	2.55040687	0.793209233
H122N	A6	3.93654806	3.86395777	4.05448448	3.95166344	1.229017992
I156H	A6	3.01177357	3.04120757	3.09767352	3.05021822	0.948656972
H122G	A6	2.26025767	2.32505141	2.68407247	2.42312718	0.75362362
V4P	A6	2.11641333	2.10055729	2.24051794	2.15249619	0.669453912
M70V	A6	2.24273007	2.23659585	2.64148373	2.37360322	0.738221032
G100K	A6	2.01992232	2.37965596	2.28895697	2.22951175	0.693406739
V4T	A6	1.82901075	1.85536976	1.81994999	1.83477683	0.570639117
R39E	A6	2.52246117	2.86293515	3.490443	2.95861311	0.920166608
M70I	A6	2.74155221	2.41723773	2.4594091	2.53939968	0.789785859
R26N	A6	2.57162854	2.5987526	3.01379061	2.72805725	0.84846078
H122D	A6	2.30481081	2.31817206	2.36814172	2.33037487	0.72477646
H36K	A6	3.20794753	2.73754923	3.02580217	2.99043298	0.930062995
Q154A	A6	2.60752189	2.42278609	2.23172773	2.42067857	0.75286207
V4S	A6	2.35945153	2.73195062	2.52072282	2.53737499	0.789156153
N38D	A6	3.46796132	2.84799076	2.62905303	2.98166837	0.92733709
I136V	A6	2.21928251	2.89052742	2.91018043	2.67333012	0.831439942
V4Q	A6	2.46753336	2.31431436	2.39756313	2.39313695	0.744296274
V120I	A6	2.33920574	2.42672261	2.42972844	2.39855226	0.745980505
R51L	A6	3.59468169	3.65579152	3.58686819	3.61244713	1.123517372
S164K	A6	2.88779041	2.67035813	3.01541933	2.85785596	0.888829842
V102L	A6	1.43179028	1.38377968	1.3680322	1.39453405	0.433717969
Q154K	A6	3.79595761	4.46470681	4.67303839	4.31123427	1.340849131
I76L	A6	3.64035427	3.67062057	2.89613104	3.40236863	1.058180265
H122E	A6	1.7415255	2.21851859	2.43246313	2.13083574	0.662717234
G50T	A6	2.05449402	2.11821368	2.14680207	2.10650325	0.655149519
I60M	A6	1.29435986	1.1319407	1.28742364	1.23790807	0.385005279
L68E	A6	3.45195421	3.15568876	3.22695693	3.27819996	1.019562218
ABE1.2	A6	3.09377222	2.58409136	3.96804102	3.21530153	1
ABE8e	A6	40.3139543	39.4689847	43.2579172	41.0136187	12.75576125
NC	A6	0.78614751	0.45382239	0.31440429	0.51812473	0.161143434
