# Supplementary material for: Zero-shot prediction of mutation effects with multimodal deep representation learning guides protein engineering
Source: Cell Res. 2024 Jul 5;34(9):630–47. doi: 10.1038/s41422-024-00989-2 (PMC11369238; doi:10.1038/s41422-024-00989-2)
Supplement: Supplementary file 25 — Supplementary information, Data S4 [file 41422_2024_989_MOESM25_ESM.pdf]

Data S4 | Amino acid sequences of plasmids used in this study.

Amino acid sequences used this study for testing of TnpB mutants gene editing efficiency, related to Fig. 5

Component	Amino acid sequence
SV40 nuclear localization signal	PKKKRKV
Nuclear localization signal	KRPAATKKAGQAKKKK
TnpB-AI-3.1	MIRNKAFVVRLYPNAAQTELINRTLGSARFVYNHFLARRI<br>AAYKESGKGLTYGQTSRELTRLKQAEETSWLREVDKFAL<br>QNSLKNLETAYKNFFRTVKQSGKKVGFPRFRKKRTGESY<br>RTQFTNNNIQIGEGRLKLPKLGWVKTKGQQDIQGKILNVT<br>VRRIHEGHYEASVLCEVEIPYLPAAPKFAAGVDVGIKDFAI<br>VTDGVRFKHEQNPKYYRSTLKRLRKAQQTLSRRKKGSAR<br>YGKAKTKLARIHKRIVNKRQDFLHKLTTSLVREYEIIGTEH<br>LKPDNMRKNRRLALSISDAGWGEFIRQLEYKAAWYGRLV<br>SKVSPYFPSSQLCHDCGFKNPEVKNLAVRTWTCPNCGET<br>HDRDENAALNIRREALVAAGISDTLNAHGGYVRPASAGN<br>GLRSENHATLVV
TnpB-AI-3.2	MIRNKAFVVRLYPNAAQTELINRTLGSARFVYNHFLARRI<br>AAYKESGKGLTYGQTSRELTLLKQAEETRWLREVDKFAL<br>QNSLKNLETAYKNFFRTVKQSGKKVGFPRFRKKRTGESY<br>RTQFTNNNIQIGEGRLKLPKLGWVKTKGQQDIQGKILNVT<br>VRRIHEGHYEASVLCEVEIPYLPAAPKFAAGVDVGIKDFAI<br>VTDGVRFKHEQNPKYYRSTLKRLRKAQQTLSRRKKGSAR<br>YGKAKTKLARIHKRIVNKRQDFLHKLTTSLVREYEIIGTEH<br>LKPDNMRKNRRLALSISDAGWGEFIRQLEYKAAWYGRLV<br>SKVSPYFPSSQLCHDCGFKNPEVKNLAVRTWTCPNCGET<br>HDRDENAALNIRREALVAAGISDTLNAHGGYVRPASAGN<br>GLRSENHATLVV
TnpB-AI-3.3	MIRNKAFVVRLYPNAAQTELINRTLGSARFVYNHFLARRI<br>AAYKESGKGLTYGQTSRELTLLKQAEETSWLREVDKFAL<br>QNSLKNLETAYKNFFRTVKQSGKKVGFPRFRKKRTGESY<br>RTQFTNNNIQIGEGRLKLPKLGWVKTRGQQDIQGKILNVT<br>VRRIHEGHYEASVLCEVEIPYLPAAPKFAAGVDVGIKDFAI<br>VTDGVRFKHEQNPKYYRSTLKRLRKAQQTLSRRKKGSAR<br>YGKAKTKLARIHKRIVNKRQDFLHKLTTSLVREYEIIGTEH<br>LKPDNMRKNRRLALSISDAGWGEFIRQLEYKAAWYGRLV<br>SKVSPYFPSSQLCHDCGFKNPEVKNLAVRTWTCPNCGET<br>HDRDENAALNIRREALVAAGISDTLNAHGGYVRPASAGN<br>GLRSENHATLVV

TnpB-AI-3.4	MIRNKAFVVRLYPNAAQTELINRTLGSARFVYNHFLARRI<br>AAYKESGKGLTYGQTSRELTLLKQAEETSWLREVDKFAL<br>QNSLKNLETAYKNFFRTVKQSGKKVGFPRFRKKRTGESY<br>RTQFTNNNIQIGEGRLKLPKLGWVKTKGQQDIQGKILNVT<br>VRRIHEGHYEASVLCEVEIPYLPAAPKFAAGVDVGIKDFAI<br>VTDGVRFRHEQNPKYYRSTLKRLRKAQQTLSRRKKGSAR<br>YGKAKTKLARIHKRIVNKRQDFLHKLTTSLVREYEIIGTEH<br>LKPDNMRKNRRLALSISDAGWGEFIRQLEYKAAWYGRLV<br>SKVSPYFPSSQLCHDCGFKNPEVKNLAVRTWTCPNCGET<br>HDRDENAALNIRREALVAAGISDTLNAHGGYVRPASAGN<br>GLRSENHATLVV
TnpB-AI-3.5	MIRNKAFVVRLYPNAAQTELINRTLGSARFVYNHFLARRI<br>AAYKESGKGLTYGQTSRELTRLKQAEETRWLSEVDKFAL<br>QNSLKNLETAYKNFFRTVKQSGKKVGFPRFRKKRTGESY<br>RTQFTNNNIQIGEGRLKLPKLGWVKTKGQQDIQGKILNVT<br>VRRIHEGHYEASVLCEVEIPYLPAAPKFAAGVDVGIKDFAI<br>VTDGVRFKHEQNPKYYRSTLKRLRKAQQTLSRRKKGSAR<br>YGKAKTKLARIHKRIVNKRQDFLHKLTTSLVREYEIIGTEH<br>LKPDNMRKNRRLALSISDAGWGEFIRQLEYKAAWYGRLV<br>SKVSPYFPSSQLCHDCGFKNPEVKNLAVRTWTCPNCGET<br>HDRDENAALNIRREALVAAGISDTLNAHGGYVRPASAGN<br>GLRSENHATLVV
TnpB-AI-3.6	MIRNKAFVVRLYPNAAQTELINRTLGSARFVYNHFLARRI<br>AAYKESGKGLTYGQTSRELTRLKQAEETSWLSEVDKFAL<br>QNSLKNLETAYKNFFRTVKQSGKKVGFPRFRKKRTGESY<br>RTQFTNNNIQIGEGRLKLPKLGWVKTRGQQDIQGKILNVT<br>VRRIHEGHYEASVLCEVEIPYLPAAPKFAAGVDVGIKDFAI<br>VTDGVRFKHEQNPKYYRSTLKRLRKAQQTLSRRKKGSAR<br>YGKAKTKLARIHKRIVNKRQDFLHKLTTSLVREYEIIGTEH<br>LKPDNMRKNRRLALSISDAGWGEFIRQLEYKAAWYGRLV<br>SKVSPYFPSSQLCHDCGFKNPEVKNLAVRTWTCPNCGET<br>HDRDENAALNIRREALVAAGISDTLNAHGGYVRPASAGN<br>GLRSENHATLVV
TnpB-AI-3.7	MIRNKAFVVRLYPNAAQTELINRTLGSARFVYNHFLARRI<br>AAYKESGKGLTYGQTSRELTLLKQAEETSWLREVDKFAL<br>QNSLKNLETAYKNFFRTVKQSGKKVGFPRFRKKRTGESY<br>RTQFTNNNIQIGEGRLKLPKLGWVKTKGQQDIQGKILNVT<br>VRRIHEGHYEASVLCEVEIPYLPAAPKFAAGVDVGIKDFAI<br>VTDGVRFKHEQNPKYYRSTLKRLRKAQQTLSRRKKGSAR<br>YGKAKTKLARIHKRIVNKRQDFLHKLTTSLVREYEIIGTEH<br>LKPDNMRKNRRLALSISDAGWGEFIRQLEYKAAWYGRLV<br>SKVSPYFPSSQLCHDCGFKNPEVKNLAVRTWTCPNCGET<br>HDRDENAALNIRREALVAAGISDTLNAHGGYVRRASAGN<br>GLRSENHATLVV

TnpB-AI-3.8	MIRNKAFVVRLYPNAAQTELINRTLGSARFVYNHFLARRIAAYKESGKGLTYGQTSRELTLLKQAEETSWLREVDKFALQNSLKNLETAYKNFFRTVKQSGKKVGFPRFRKKRTGESYRTQFTNNNIQIGEGRLKLPKLGWVKTKGQQDIQGKILNVTVRRIHEGHYEASVLCEVEIPYLPAAPKFAAGVDVGIKDFAIVTDGVRFKHEQNPKYYRSTLKRLRKAQQTLSRRKKGSARYGKAKTKLARIHKRIVNKRQDFLHKLTTSLVREYEIIGTEHLKPDNMRKNRRLALSISDAGWGEFIRQLEYKAAWYGRLVSKVSPYFPSSQLCHDCGFKNPEVKNLAVRTWTCPNCGETHDRDENAALNIRREALVAAGISDTRNAHGGYVRPASAGNGLRSENHATLVV
TnpB-AI-3.9	MIRNKAFVVRLYPNAAQTELINRTLGSARFVYNHFLARRIAAYKESGKGLTYGQTSRELTRLKQAEETSWLSEVDKFALQNSLKNLETAYKNFFRTVKQSGKKVGFPRFRKKRTGESYRTQFTNNNIQIGEGRLKLPKLGWVKTKGQQDIQGKILNVTVRRIHEGHYEASVLCEVEIPYLPAAPKFAAGVDVGIKDFAIVTDGVRFRHEQNPKYYRSTLKRLRKAQQTLSRRKKGSARYGKAKTKLARIHKRIVNKRQDFLHKLTTSLVREYEIIGTEHLKPDNMRKNRRLALSISDAGWGEFIRQLEYKAAWYGRLVSKVSPYFPSSQLCHDCGFKNPEVKNLAVRTWTCPNCGETHDRDENAALNIRREALVAAGISDTLNAHGGYVRPASAGNGLRSENHATLVV
TnpB-AI-3.10	MIRNKAFVVRLYPNAAQTELINRTLGSARFVYNHFLARRIAAYKESGKGLTYGQTSRELTLLKQAEETSWLREVDKFALQNSLKNLETAYKNFFRTVKQSGKKVGFPRFRKKRTGESYRTQFTNNNIQIGEGRLKLPKLGWVKTKGQQDIQGKILNVTVRRIHEGHYEASVLCEVEIPYLPAAPKFAAGVDVGIKDFAIVTDGVRFKHEQNPKYYRSTLKRLRKAQQTLSRRKKGSARYGKAKTKLARIHKRIVNKRQDFLHKLTTRLVREYEIIGTEHLKPDNMRKNRRLALSISDAGWGEFIRQLEYKAAWYGRLVSKVSPYFPSSQLCHDCGFKNPEVKNLAVRTWTCPNCGETHDRDENAALNIRREALVAAGISDTLNAHGGYVRPASAGNGLRSENHATLVV
TnpB-AI-5.1	MIRNKAFVVRLYPNAAQTELINRTLGSARFVYNHFLARRIAAYKESGKGLTYGQTSRELTLLKQAEETSWLREVDKFALQNSLKNLETAYKNFFRRVKQSGKKVGFPRFRKKRTGESYRTQFTNNNIQIGEGRLKLPKLGWVKTKGQQDIQGKILNVTVRRIHEGHYRASVLCEVEIPYLPAAPKFAAGVDVGIKDFAIVTDGVRFKHEQNPKYYRSTLKRLRKAQQTLSRRKKGSARYGKAKTKLARIHKRIVNKRQDFLHKLTTSLVREYEIIGTEHLKPDNMRKNRRLALSISDAGWGEFIRQLEYKAAWYGRLVSKVSPYFPSSQLCHDCGFKNPEVKNLAVRTWTCPNCGETHDRDENAALNIRREALRAAGISDTLNAHGGYVRPASAGNGLRSENHATLVV

TnpB-AI-5.2	MIRNKAFVVRLYPNAAQTELINRTLGSARFVYNHFLARRI<br>AAYKESGKGLTYGQTSRELTLLKQAEETSWLREVDKFAL<br>QNSLRNLETAYKNFFRTVKQSGKKVGFPRFRKKRTGESYR<br>TQFTNNNIQIGEGRLKLPKLGWVKTKGQQDIQGKILNVTV<br>RRIHEGHYRASVLCEVEIPYLPAAPKFAAGVDVGIKDFAIV<br>TDGVRFKHEQNPKYYRSTLKRLRKAQQTLSRRKKGSARY<br>GKAKTKLARIHKRIVNKRQDFLHKLTTSLVREYEIIGTEHL<br>KPDNMRKNRRLALSISDAGWGEFIRQLEYKAAWYGRLVS<br>KVSPYFPSSQLCHDCGFKNPEVKNLAVRTWTCPNCGETH<br>DRDENAALNIRREALRAAGISDTLNAHGGYVRPASAGNG<br>LRSENHATLVV
TnpB-AI-5.3	MIRNKAFVVRLYPNAAQTELINRTLGSARFVYNHFLARRI<br>AAYKESGKGLTYGQTSRELTLLKQAEETSWLREVDKFAL<br>QNSLKNLETAYKNFFRTVKQSGKKVGFPRFRKKRTGESY<br>RTQFTNNNIQIGEGRLKLPKLGWVKTKGQQDIQGKILNVT<br>VRRIHEGHYRASVLCEVEIPYLPAAPKFAAGVDVGIKDFAI<br>VTDGVRFKHEQNPKYYRSTLKRLRKAQQTLSRRKKGSAR<br>YGKAKTKLARIHRRIVNKRQDFLHKLTTSLVREYEIIGTEH<br>LKPDNMRKNRRLALSISDAGWGEFIRQLEYKAAWYGRLV<br>SKVSPYFPSSQLCHDCGFKNPEVKNLAVRTWTCPNCGET<br>HDRDENAALNIRREALRAAGISDTLNAHGGYVRPASAGN<br>GLRSENHATLVV
TnpB-AI-5.4	MIRNKAFVVRLYPNAAQTELINRTLGSARFVYNHFLARRI<br>AAYKESGKGLTYGQTSRELTLLKQAEETSWLREVDKFAL<br>QNSLRNLETAYKNFFRRVKQSGKKVGFPRFRKKRTGESY<br>RTQFTNNNIQIGEGRLKLPKLGWVKTKGQQDIQGKILNVT<br>VRRIHEGHYRASVLCEVEIPYLPAAPKFAAGVDVGIKDFAI<br>VTDGVRFKHEQNPKYYRSTLKRLRKAQQTLSRRKKGSAR<br>YGKAKTKLARIHKRIVNKRQDFLHKLTTSLVREYEIIGTEH<br>LKPDNMRKNRRLALSISDAGWGEFIRQLEYKAAWYGRLV<br>SKVSPYFPSSQLCHDCGFKNPEVKNLAVRTWTCPNCGET<br>HDRDENAALNIRREALVAAGISDTLNAHGGYVRPASAGN<br>GLRSENHATLVV
TnpB-AI-5.5	MIRNKAFVVRLYPNAAQTELINRTLGSARFVYNHFLARRI<br>AAYKESGKGLTYGQTSSELTLLKQAEETSWLREVDKFAL<br>QNSLRNLETAYKNFFRRVKQSGKKVGFPRFRKKRTGESY<br>RTQFTNNNIQIGEGRLKLPKLGWVKTKGQQDIQGKILNVT<br>VRRIHEGHYRASVLCEVEIPYLPAAPKFAAGVDVGIKDFAI<br>VTDGVRFKHEQNPKYYRSTLKRLRKAQQTLSRRKKGSAR<br>YGKAKTKLARIHKRIVNKRQDFLHKLTTSLVREYEIIGTEH<br>LKPDNMRKNRRLALSISDAGWGEFIRQLEYKAAWYGRLV<br>SKVSPYFPSSQLCHDCGFKNPEVKNLAVRTWTCPNCGET<br>HDRDENAALNIRREALRAAGISDTLNAHGGYVRPASAGN<br>GLRSENHATLVV

TnpB-AI-5.6	MIRNKAFVVRLYPNAAQTELINRTLGSARFVYNHFLARRI<br>AAYKESGKGLTYGQTSSELTLLKQAEETSWLREVDKFAL<br>QNSLRNLETAYKNFFRTVKQSGKKVGFPRFRKKRTGESYR<br>TQFTNNNIQIGEGRLKLPKLGWVKTKGQQDIQGKILNVTV<br>RRIHEGHYRASVLCEVEIPYLPAAPKFAAGVDVGIKDFAIV<br>TDGVRFKHEQNPKYYRSTLKRLRKAQQTLSRRKKGSARY<br>GKAKTKLARIHRRIVNKRQDFLHKLTTSLVREYEIIGTEHL<br>KPDNMRKNRRLALSISDAGWGEFIRQLEYKAAWYGRLVS<br>KVSPYFPSSQLCHDCGFKNPEVKNLAVRTWTCPNCGETH<br>DRDENAALNIRREALRAAGISDTLNAHGGYVRPASAGNG<br>LRSENHATLVV
TnpB-AI-5.7	MIRNKAFVVRLYPNAAQTELINRTLGSARFVYNHFLARRI<br>AAYKESGKGLTYGQTSSELTLLKQAEETSWLREVDKFAL<br>QNSLKNLETAYKNFFRRVKQSGKKVGFPRFRKKRTGESY<br>RTQFTNNNIQIGEGRLKLPKLGWVKTKGQQDIQGKILNVT<br>VRRIHEGHYRASVLCEVEIPYLPAAPKFAAGVDVGIKDFAI<br>VTDGVRFKHEQNPKYYRSTLKRLRKAQQTLSRRKKGSAR<br>YGKAKTKLARIHRRIVNKRQDFLHKLTTSLVREYEIIGTEH<br>LKPDNMRKNRRLALSISDAGWGEFIRQLEYKAAWYGRLV<br>SKVSPYFPSSQLCHDCGFKNPEVKNLAVRTWTCPNCGET<br>HDRDENAALNIRREALRAAGISDTLNAHGGYVRPASAGN<br>GLRSENHATLVV
TnpB-AI-5.8	MIRNKAFVVRLYPNAAQTELINRTLGSARFVYNHFLARRI<br>AAYKESGKGLTYGQTSRELTLLKQAEETSWLREVDKFAL<br>QNSLKNLETAYKNFFRTVKQSGKKVGFPRFRKKRTGESY<br>RTQFTNNNIQIGEGRLKLPKLGWVKTKGQQDIQGKILNVT<br>VRRIHEGHYRASVLCEVEIPYLPAAPKFAAGVDVGIKDFAI<br>VTDGVRFKHEQNPKYYRSTLKRLRKAQQTLSRRKKGSAR<br>YGKAKTKLARIHKRIVNKRQDFLHKLTTSLVREYEIIGTEH<br>LKPDNMRKNRRLALSISDAGWGEFIRQLEYKAAWYGRLV<br>SKVSRYFPSSQLCHDCGFKNPEVKNLAVRTWTCPNCGET<br>HDRDENAALNIRREALRAAGISDTLNAHGGYVRPASAGN<br>GLRSENHATLVV
TnpB-AI-5.9	MIRNKAFVVRLYPNAAQTELINRTLGSARFVYNHFLARRI<br>AAYKESGKGLTYGQTSRELTRLKQAEETSWLREVDKFAL<br>QNSLKNLETAYKNFFRTVKQSGKKVGFPRFRKKRTGESY<br>RTQFTNNNIQIGEGRLKLPKLGWVKTKGQQDIQGKILNVT<br>VRRIHEGHYRASVLCEVEIPYLPAAPKFAAGVDVGIKDFAI<br>VTDGVRFKHEQNPKYYRSTLKRLRKAQQTLSRRKKGSAR<br>YGKAKTKLARIHKRIVNKRQDFLHKLTTSLVREYEIIGTEH<br>LKPDNMRKNRRLALSISDAGWGEFIRQLEYKAAWYGRLV<br>SKVSPYFPSSQLCHDCGFKNPEVKNLAVRTWTCPNCGET<br>HDRDENAALNIRREALRAAGISDTLNAHGGYVRPASAGN<br>GLRSENHATLVV

TnpB-AI-5.10	MIRNKAFVVRLYPNAAQTELINRTLGSARFVYNHFLARRI<br>AAYKESGKGLTYGQTSRELTLLKQAEETSWLREVDKFAL<br>QNSLKNLETAYKNFFRTVKRSGKKVGFPRFRKKRTGESYR<br>TQFTNNNIQIGEGRLKLPKLGWVKTKGQQDIQGKILNVTV<br>RRIHEGHYRASVLCEVEIPYLPAAPKFAAGVDVGIKDFAIV<br>TDGVRFKHEQNPKYYRSTLKRLRKAQQTLSRRKKGSARY<br>GKAKTKLARIHKRIVNKRQDFLHKLTTSLVREYEIIGTEHL<br>KPDNMRKNRRLALSISDAGWGEFIRQLEYKAAWYGRLVS<br>KVSPYFPSSQLCHDCGFKNPEVKNLAVRTWTCPNCGETH<br>DRDENAALNIRREALRAAGISDTLNAHGGYVRPASAGNG<br>LRSENHATLVV
dTnpB-D191A	MIRNKAFVVRLYPNAAQTELINRTLGSARFVYNHFLARRI<br>AAYKESGKGLTYGQTSSELTLLKQAEETSWLSEVDKFALQ<br>NSLKNLETAYKNFFRTVKQSGKKVGFPRFRKKRTGESYRT<br>QFTNNNIQIGEGRLKLPKLGWVKTKGQQDIQGKILNVTVR<br>RIHEGHYEASVLCEVEIPYLPAAPKFAAGVAVGIKDFAIVT<br>DGVRFKHEQNPKYYRSTLKRLRKAQQTLSRRKKGSARYG<br>KAKTKLARIHKRIVNKRQDFLHKLTTSLVREYEIIGTEHLK<br>PDNMRKNRRLALSISDAGWGEFIRQLEYKAAWYGRLVSK<br>VSPYFPSSQLCHDCGFKNPEVKNLAVRTWTCPNCGETHD<br>RDENAALNIRREALVAAGISDTLNAHGGYVRPASAGNGL<br>RSENHATLVV
dTnpB-AI-D191A ·S72R ·K84R · E168R ·K251R ·V374R	MIRNKAFVVRLYPNAAQTELINRTLGSARFVYNHFLARRI<br>AAYKESGKGLTYGQTSSELTLLKQAEETSWLREVDKFAL<br>QNSLRNLETAYKNFFRTVKQSGKKVGFPRFRKKRTGESYR<br>TQFTNNNIQIGEGRLKLPKLGWVKTKGQQDIQGKILNVTV<br>RRIHEGHYRASVLCEVEIPYLPAAPKFAAGVAVGIKDFAIV<br>TDGVRFKHEQNPKYYRSTLKRLRKAQQTLSRRKKGSARY<br>GKAKTKLARIHRRIVNKRQDFLHKLTTSLVREYEIIGTEHL<br>KPDNMRKNRRLALSISDAGWGEFIRQLEYKAAWYGRLVS<br>KVSPYFPSSQLCHDCGFKNPEVKNLAVRTWTCPNCGETH<br>DRDENAALNIRREALRAAGISDTLNAHGGYVRPASAGNG<br>LRSENHATLVV
XTEN linker	SGGSSGGSSGSETPGTSESATPESSGGSSGGS

TadA-linker-TadA*	SEVEFSHEYWMRHALTLAKRAWDEREVPVGAVLVHNNRVIGEGWNRPIGRHDPTAHAEIMALRQGGLVMQNYRLIDATLYVTLEPCVMCAGAMIHSRIGRVVFGARDAKTGAAGSLMDVLHHPGMNHRVEITEGILADECAALLSDFFRMRRQEIKAQKKAQSSTDSGGSSGGSSGSETPGTSESATPESSGGSSGGSSEVEFSHEYWMRHALTLAKRARDEREVPVGAVLVLNNRVIGEGWNRAIGLHDPTAHAEIMALRQGGLVMQNYRLIDATLYVTFEPCVMCAGAMIHSRIGRVVFGVRNSKRGAAGSLMNVLNYPGMNHRVEITEGILADECAALLCDFYRMPRQVFNAQKKAQSSIN
APOBEC3A*(Y130F)	EASPASGPRHLMDPHIFTSNFNNGIGRHKTYLCYEVERLDNGTSVKMDQHRGFLHNQAKNLLCGFYGRHAELRFLDLVPSLQLDPAQIYRVTWFISWSPCFSWGCAGEVRAFLQENTHVRLRIFAARIFDYDPLYKEALQMLRDAGAQVSIMTYDEFKHCWDTFVDHQGCPFQPWDGLDEHSQALSGRLRAILQNQGN
UGI-linker-UGI	TNLSDIIEKETGKQLVIQESILMLPEEVEEVIGNKPESDILVHTAYDESTDENVMLLTSDAPEYKPWALVIQDSNGENKIKMLSGGSGGSGGSTNLSDIIEKETGKQLVIQESILMLPEEVEEVIGNKPESDILVHTAYDESTDENVMLLTSDAPEYKPWALVIQDSNGENKIKML

Amino acid sequences used this study for testing of TadA mutants base editing efficiency, related to Fig. 6	
SV40 nuclear localization signal (NLS)	PKKKRKV
ABE8e	SEVEFSHEYWMRHALTLAKRARDEREVPVGAVLVLNNRVIGEGWNRAIGLHDPTAHAEIMALRQGGLVMQNYRLIDATLYVTFEPCVMCAGAMIHSRIGRVVFGVRNSKRGAAGSLMNVLNYPGMNHRVEITEGILADECAALLCDFYRMPRQVFNAQKKAQSSIN

ABE9	SEVEFSHEYWMRHALTLAKRARDEREVPVGAVLVLNNR VIGEGWNRAIGLHDPTAHAEIMALRQGGLVMQNYRLIDA TLYVTFEPCVMCAGAMIHSRIGRVVFGVRQSKRGAAGSL MNVLNYPGMNHRVEITEGILADECAALTCDFYRMPRQVF NAQKKAQSSIN
linker	SGGSSGGSSGSETPGTSESATPESSGGSSGGS
nSpCas9	DKKYSIGLAIGTNSVGWAVITDEYKVPSKKFKVLGNTDRH SIKKNLIGALLFDSGETAEATRLKRTARRRYTRRKNRICYL QEIFSNEMAKVDDSFFHRLEESFLVEEDKKHERHPIFGNIV DEVAYHEKYPTIYHLRKKLVDSTDKADLRLIYLALAHMIK FRGHFLIEGDLNPDNSDVDKLFIQLVQTYNQLFEENPINAS GVDAKAILSARLSKSRRLENLIAQLPGEKKNGLFGNLIALS LGLTPNFKSNFDLAEDAKLQLSKDTYDDDLDNLLAQIGDQ YADLFLAAKNLSDAILLSDILRVNTEITKAPLSASMIKRYD EHHQDLTLLKALVRQQLPEKYKEIFFDQSKNGYAGYIDGG ASQEEFYKFIKPILEKMDGTEELLVKLNREDLLRKQRTFDN GSIPHQIHLGELHAILRRQEDFYPFLKDNREKIEKILTFRIPY YVGPLARGNSRFAWMTRKSEETITPWNFEEVVDKGASAQ
Nuclear localization signal	KRPAATKKAGQAKKKK

TadA-A106V D108N（ABE1.2)	SEVEFSHEYWMRHALTLAKRAWDEREVPVGAVLVHNNRVIGEGWNRPIGRHDPTAHAEIMALRQGGLVMQNYRLIDATLYVTLEPCVMCAGAMIHSRIGRVVFGVRNAKTGAAGSLMDVLHHPGMNHRVEITEGILADECAALLSDFFRMRRQEIKAQKKAQSSTD
TadA-AI-8	SEVEFSDEYWMRHALTLAKRAWDEREVPVGAVLVHNNRVIGEGWNRPIGLHDPTAHAEIMALRQGGEVLQNYRLLDATLYVTLEPCVMCAGAMIHSRIGRVVFGVRNAKTGAAGSLMDVLNHPGMNHRVEITGGILADECAALLSDFFRMRRKEIKAQKKAQSSTD
TadA-AI-10.1	SEVEFSDEYWMRHALTLAKRAWDEGEVPVGAVLVHNNRVIGEGWNRPIGLHDPTAHAEIMALRQGGEVLQNYRLLDATLYVTLEPCVMCAGAMIHSRIGRVVFGVRNAKTGAAGSLMDVLNHPGMNHRVEITGGILADECAALLSDFFRMRRKEHKAQKKAQSSTD
TadA-AI-10.2	SEVEFSDEYWMRHALTLAKRAWDEGEVPVGAVLVHNNEVIGEGWNRPIGLHDPTAHAEIMALRQGGEVLQNYRLLDATLYVTLEPCVMCAGAMIHSRIGRVVFGVRNAKTGAAGSLMDVLNHPGMNHRVEITGGILADECAALLSDFFRMRRKEIKAQKKAQSSTD

TadA-AI-10.3	SEVEFSDEYWMRHALTLAKRAWDEGEVPVGAVLVKN NRVIGEGWNRPIGLHDPTAHAEIMALRQGGEVLQNYRL LDATLYVTLEPCVMCAGAMIHSRIGRVVFGVRNAKTG AAGSLMDVLNHPGMNHRVEITGGILADECAALLSDFFR MRRKEIKAQKKAQSSTD
TadA-AI-10.4	SEVEFSDEYWMRHALTLAKRAWDEGEVPVGAVLVHN DRVIGEGWNRPIGLHDPTAHAEIMALRQGGEVLQNYRL LDATLYVTLEPCVMCAGAMIHSRIGRVVFGVRNAKTG AAGSLMDVLNHPGMNHRVEITGGILADECAALLSDFFR MRRKEIKAQKKAQSSTD
TadA-AI-10.5	SENEFSDEYWMRHALTLAKRAWDEGEVPVGAVLVHN NRVIGEGWNRPIGLHDPTAHAEIMALRQGGEVLQNYRL LDATLYVTLEPCVMCAGAMIHSRIGRVVFGVRNAKTG AAGSLMDVLNHPGMNHRVEITGGILADECAALLSDFFR MRRKEIKAQKKAQSSTD
TadA-AI-10.6	SEVSFSDEYWMRHALTLAKRAWDEGEVPVGAVLVHN NRVIGEGWNRPIGLHDPTAHAEIMALRQGGEVLQNYRL LDATLYVTLEPCVMCAGAMIHSRIGRVVFGVRNAKTG AAGSLMDVLNHPGMNHRVEITGGILADECAALLSDFFR MRRKEIKAQKKAQSSTD

TadA-AI-10.7	SEVTFSDEYWMRHALTLAKRAWDEGEVPVGAVLVHN NRVIGEGWNRPIGLHDPTAHAEIMALRQGGEVLQNYRL LDATLYVTLEPCVMCAGAMIHSRIGRVVFGVRNAKTG AAGSLMDVLNHPGMNHRVEITGGILADECAALLSDFFR MRRKEIKAQKKAQSSTD
TadA-AI-10.8	SEKEFSDEYWMRHALTLAKRAWDEGEVPVGAVLVHN NRVIGEGWNRPIGLHDPTAHAEIMALRQGGEVLQNYRL LDATLYVTLEPCVMCAGAMIHSRIGRVVFGVRNAKTG AAGSLMDVLNHPGMNHRVEITGGILADECAALLSDFFR MRRKEIKAQKKAQSSTD
TadA-AI-10.9	SEVPFSDEYWMRHALTLAKRAWDEGEVPVGAVLVHN NRVIGEGWNRPIGLHDPTAHAEIMALRQGGEVLQNYRL LDATLYVTLEPCVMCAGAMIHSRIGRVVFGVRNAKTG AAGSLMDVLNHPGMNHRVEITGGILADECAALLSDFFR MRRKEIKAQKKAQSSTD
TadA-AI-10.10	SEVEFSDEYWMRHALTLAKRAWDEGEVPVGAVLVHN NKVIGEGWNRPIGLHDPTAHAEIMALRQGGEVLQNYRL LDATLYVTLEPCVMCAGAMIHSRIGRVVFGVRNAKTG AAGSLMDVLNHPGMNHRVEITGGILADECAALLSDFFR MRRKEIKAQKKAQSSTD

TadA-AI-12.1	SEVEFSDEYWMRHALTLAKRAWDEGEVPVGAVLVKN<br>NEVIGEGWNRPIGLHDPTAHAEIMALRQGGEVLQNYRL<br>LDATLYVTLEPCVMCAGAMIHSRIGRVVFGVRNAKTG<br>AAGSLMDVLNHPGMNHRVEITGGILADECAALLSDFFR<br>MRRKEHKAQKKAQSSTD
TadA-AI-12.2	SEVEFSDEYWMRHALTLAKRAWDEGEVPVGAVLVHN<br>DEVIGEGWNRPIGLHDPTAHAEIMALRQGGEVLQNYRL<br>LDATLYVTLEPCVMCAGAMIHSRIGRVVFGVRNAKTG<br>AAGSLMDVLNHPGMNHRVEITGGILADECAALLSDFFR<br>MRRKEHKAQKKAQSSTD
TadA-AI-12.3	SEVEFSDEYWMRHALTLAKRAWDEGEVPVGAVLVKN<br>DRVIGEGWNRPIGLHDPTAHAEIMALRQGGEVLQNYRL<br>LDATLYVTLEPCVMCAGAMIHSRIGRVVFGVRNAKTG<br>AAGSLMDVLNHPGMNHRVEITGGILADECAALLSDFFR<br>MRRKEHKAQKKAQSSTD
TadA-AI-12.4	SENEFSDEYWMRHALTLAKRAWDEGEVPVGAVLVHN<br>NEVIGEGWNRPIGLHDPTAHAEIMALRQGGEVLQNYRL<br>LDATLYVTLEPCVMCAGAMIHSRIGRVVFGVRNAKTG<br>AAGSLMDVLNHPGMNHRVEITGGILADECAALLSDFFR<br>MRRKEHKAQKKAQSSTD

TadA-AI-12.5	SEVSFSDEYWMRHALTLAKRAWDEGEVPVGAVLVHN NEVIGEGWNRPIGLHDPTAHAEIMALRQGGEVLQNYRL LDATLYVTLEPCVMCAGAMIHSRIGRVVFGVRNAKTG AAGSLMDVLNHPGMNHRVEITGGILADECAALLSDFFR MRRKEHKAQKKAQSSTD
TadA-AI-12.6	SEVTFSDEYWMRHALTLAKRAWDEGEVPVGAVLVHN NEVIGEGWNRPIGLHDPTAHAEIMALRQGGEVLQNYRL LDATLYVTLEPCVMCAGAMIHSRIGRVVFGVRNAKTG AAGSLMDVLNHPGMNHRVEITGGILADECAALLSDFFR MRRKEHKAQKKAQSSTD
TadA-AI-12.7	SEKEFSDEYWMRHALTLAKRAWDEGEVPVGAVLVHN NEVIGEGWNRPIGLHDPTAHAEIMALRQGGEVLQNYRL LDATLYVTLEPCVMCAGAMIHSRIGRVVFGVRNAKTG AAGSLMDVLNHPGMNHRVEITGGILADECAALLSDFFR MRRKEHKAQKKAQSSTD
TadA-AI-12.8	SEVPFSDEYWMRHALTLAKRAWDEGEVPVGAVLVHN NEVIGEGWNRPIGLHDPTAHAEIMALRQGGEVLQNYRL LDATLYVTLEPCVMCAGAMIHSRIGRVVFGVRNAKTG AAGSLMDVLNHPGMNHRVEITGGILADECAALLSDFFR MRRKEHKAQKKAQSSTD

TadA-AI-12.9	SEVEFSDEYWMRHALTLAKRAWDEGEVPVGAVLVHN<br>NEVIGEGWNRPIGLHDPTAHAEIMALRQGGEVLQNYRL<br>LDATLYVTLEPCVMCAGAMIHSRIGRVVFGVRNAKTG<br>AAGSLMDVLNHPGMNHRVEITGGILADECAALLSDFFR<br>MRRKEHKAQKKAQSSED
TadA-AI-12.10	SENEFSDEYWMRHALTLAKRAWDEGEVPVGAVLVKN<br>NRVIGEGWNRPIGLHDPTAHAEIMALRQGGEVLQNYRL<br>LDATLYVTLEPCVMCAGAMIHSRIGRVVFGVRNAKTG<br>AAGSLMDVLNHPGMNHRVEITGGILADECAALLSDFFR<br>MRRKEHKAQKKAQSSTD
TadA-AI-14	SEVEFSDEYWMRHALTLAKRAWDEGEVPVGAVLVKN<br>DEVIGEGWNRPIGLHDPTAHAEIMALRQGGEVLQNYRL<br>LDATLYVTLEPCVMCAGAMIHSRIGRVVFGVRNAKTG<br>AAGSLMDVLNHPGMNHRVEITGGILADECAALLSDFFR<br>MRRKEHKAQKKAQKSTD
TadA-AI-15.1	SENEFSDEYWMRHALTLAKRAWDEGEVPVGAVLVKN<br>DEVIGEGWNRPIGLHDPTAHAEIMALRQGGEVLQNYRL<br>LDATLYVTLEPCVMCAGAMIHSRIGRVVFGVRNAKTG<br>AAGSLMDVLNHPGMNHRVEITGGILADECAALLSDFFR<br>MRRKEHKAQKKAQKSTD

TadA-AI-15.2	SEVSFSDEYWMRHALTLAKRAWDEGEVPVGAVLVKNDEVIGEGWNRPIGLHDPTAHAEIMALRQGGEVLQNYRLLDATLYVTLEPCVMCAGAMIHSRIGRVVFGVRNAKTGAAGSLMDVLNHPGMNHRVEITGGILADECAALLSDFFRMRRKEHKAQKKAQKSTD
TadA-AI-15.3	SEVTFSDEYWMRHALTLAKRAWDEGEVPVGAVLVKNDEVIGEGWNRPIGLHDPTAHAEIMALRQGGEVLQNYRLLDATLYVTLEPCVMCAGAMIHSRIGRVVFGVRNAKTGAAGSLMDVLNHPGMNHRVEITGGILADECAALLSDFFRMRRKEHKAQKKAQKSTD
TadA-AI-15.4	SEKEFSDEYWMRHALTLAKRAWDEGEVPVGAVLVKNDEVIGEGWNRPIGLHDPTAHAEIMALRQGGEVLQNYRLLDATLYVTLEPCVMCAGAMIHSRIGRVVFGVRNAKTGAAGSLMDVLNHPGMNHRVEITGGILADECAALLSDFFRMRRKEHKAQKKAQKSTD
TadA-AI-15.5	SEVPFSDEYWMRHALTLAKRAWDEGEVPVGAVLVKNDEVIGEGWNRPIGLHDPTAHAEIMALRQGGEVLQNYRLLDATLYVTLEPCVMCAGAMIHSRIGRVVFGVRNAKTGAAGSLMDVLNHPGMNHRVEITGGILADECAALLSDFFRMRRKEHKAQKKAQKSTD

TadA-AI-15.6	SEVEFSDEYWMRHALTLAKRAWDEGEVPVGAVLVKN DEVIGEGWNRPIGLHDPTAHAEIMALRQGGEVLQNYRL LDATLYVTLEPCVMCAGAMIHSRIGRVVFGVRNAKTG AAGSLMDVLNHPGMNHRVEITGGILADECAALLSDFFR MRRKEHKAQKKAQKSED
TadA-AI-15.7	SEVEFSDEYWMRHALTLAKRAWDEGEVPVGAVLVKN DEVIGEGWNRPIGLHDPTAHAEIMALRQGGEVLQNYRL LDATLYVTLEPCVMCAGALIHSRIGRVVFGVRNAKTGA AGSLMDVLNHPGMNHRVEITGGILADECAALLSDFFRM RRKEHKAQKKAQKSTD
TadA-AI-15.8	SEVEFSDEYWMRHALTLAKRAWDEGEVPVGAVLVKN DEVIGEGWNRPIGLHDPSAHAEIMALRQGGEVLQNYRL LDATLYVTLEPCVMCAGAMIHSRIGRVVFGVRNAKTG AAGSLMDVLNHPGMNHRVEITGGILADECAALLSDFFR MRRKEHKAQKKAQKSTD
TadA-AI-15.9	SEVEFSDEYWMRHALTLAKRAWDEGEVPVGAVLVKN DEVIGEGWNRPIGLHDPTAHAEIMALRQGGEVLQNYRL LDATLYVTLEPCVMCAGAMIHSRIGRVVFGVRNAKTG AAGSLMDVLNHPGMNHRVEITGGILADECAALLSDFFR MRRKEHKAQKKAQKSAD

TadA-AI-15.10	SEVEFSDEYWMRHALTLAKRAWDEGEVPVGAVLVKN DEVIGEGWNRPIGLHDPTAHAEIMALRQGGEVLQNYRL LDATLYVTLEPCVMCAGAMIHSRIGRVVFGVRNAKTG AAGSLMDVLNHPGMNHRVEITGGILADECAALLSDFFR MRRKEHKAQKKAQKSSD
TadA-AI-15.11	SEVEFSDEYWMRHALTLAKRAWDEGEVPVGAVLVKN DEVIGEGWNRPIGLHDPTAHAEIMALRQGGEVLQNYRL LDATLYVTLEPCVMCAGAMIHSRIGRVVFGVRNAKTG AAGSLMDVLNHPGMNHRVEVTGGILADECAALLSDFF RMRRKEHKAQKKAQKSTD
TadA-AI-15.12	SEAEFSDEYWMRHALTLAKRAWDEGEVPVGAVLVKN DEVIGEGWNRPIGLHDPTAHAEIMALRQGGEVLQNYRL LDATLYVTLEPCVMCAGAMIHSRIGRVVFGVRNAKTG AAGSLMDVLNHPGMNHRVEITGGILADECAALLSDFFR MRRKEHKAQKKAQKSTD
TadA-AI-15.13	SEVEFSDEYWMRHALTLAKRAWDEGEVPVGAVLVKN DEVIGEGWNRPIGLHDPTAHAEIMALRQGGEVLQNYRL LDATLYVTLEPCVMCAGAIIHSRIGRVVFGVRNAKTGA AGSLMDVLNHPGMNHRVEITGGILADECAALLSDFFRM RRKEHKAQKKAQKSTD

TadA-AI-15.14	SEVEFSDEYWMRHALTLAKRAWDEGEVPVGAVLVKN DEVIGEGWNRPIGLHDPTAHAEIMALRQGGEVLQNYRL LDATLYVTLEPCVMCAGAMIHSRIGRVVFGVRNAKTG AAGSLMDVLNHPGLNHRVEITGGILADECAALLSDFFR MRRKEHKAQKKAQKSTD
TadA-AI-15.15	SEVQFSDEYWMRHALTLAKRAWDEGEVPVGAVLVKN DEVIGEGWNRPIGLHDPTAHAEIMALRQGGEVLQNYRL LDATLYVTLEPCVMCAGAMIHSRIGRVVFGVRNAKTG AAGSLMDVLNHPGMNHRVEITGGILADECAALLSDFFR MRRKEHKAQKKAQKSTD
TadA-AI-15.16	SEVEFSDEYWMRHALTLAKRAWDEGEVPVGAVLVKN DEVIGEGWNRPIGLHDPTAHAEIMALRQGGEVLQNYRL LDATLYVTLEPCVMCAGAMVHSRIGRVVFGVRNAKTG AAGSLMDVLNHPGMNHRVEITGGILADECAALLSDFFR MRRKEHKAQKKAQKSTD
TadA-AI-15.17	SEVEFSDEYWMRHALTLAKRAWDEGEVPVGAVLVKN DEVIGEGWNRPIGLHDPTAHAEIMALRQGGEVLQNYRL LDATLYVTLEPCVMCAGAMIHSRIQRVVFGVRNAKTG AAGSLMDVLNHPGMNHRVEITGGILADECAALLSDFFR MRRKEHKAQKKAQKSTD

TadA-AI-15.18	SEVEFSDEYWMRHALTLAKRAWDEGEVPVGAVLVKN DEVIGEGWNRPIGLHDPTAHAEIMALRQGGEVLQNYRL LDATLYVTLEPCVMCAGAMIHSRIGRVVFGVRNAKTG AAGSLMDVLNHPGMNHHVEITGGILADECAALLSDFFR MRRKEHKAQKKAQKSTD
TadA-AI-15.19	SEVEFSDEYWMRHALTLAKRAWDEGEVPVGAVLVKN DEVIGEGWNRPIGLHDPTAHAEIMALRQGGEVLQNYRL LDATLYVTLEPCVMCAGAMIHSRIGRVVFGVRNAKTG AAGSLMDVLNHPGMNHRVEITGGILAEECAALLSDFFR MRRKEHKAQKKAQKSTD
TadA-AI-15.20	SEVEFSDEYWMRHALTLAKRAWDEGEVPVGAVLVKN DEVIGEGWNRPIGLHDPTAHAEIMALRQGGEVLQNYRL LDATLYVTLEPCVMCAGAMIHSRIGRVVFGVRNAKTG AAGSLMDVLNHPGMNHRVEITGGILADECAALLSDFFR MRRKEHKAQKKAQKSDD
