# Supplementary material for: Zero-shot prediction of mutation effects with multimodal deep representation learning guides protein engineering
Source: Cell Res. 2024 Jul 5;34(9):630–47. doi: 10.1038/s41422-024-00989-2 (PMC11369238; doi:10.1038/s41422-024-00989-2)
Supplement: Supplementary file 26 — Supplementary information, Data S5 [file 41422_2024_989_MOESM26_ESM.pdf]

Data S5 | The list of target sites and primers.

TnpB target site	spacer sequence	TAM	PCR1 forward primer	PCR1 reverse primer
***EMX1* site1**	CTGTTTCTCAGGATGTTTGG	TTGAT	ACACTCTTTCCCTACACGACGCTCTTCCGATCT(barcode)ggtggttcaggcctccttcccac	GTGACTGGAGTTCAGACGTGTGCTCTTCCGATCT(barcode)caagatgctaagtgatgacagg
***EMX1* site2**	CTGCCCTCGTGGGTTTGTGGTT	TTGAT	ACACTCTTTCCCTACACGACGCTCTTCCGATCT(barcode)ggcccaggtgaaggtgtggttcc	GTGACTGGAGTTCAGACGTGTGCTCTTCCGATCT(barcode)gtacaaacggcagaagctgg
***EMX1* site3**	GCCCAAAGGTCAGATGATAG	TTGAT	ACACTCTTTCCCTACACGACGCTCTTCCGATCT(barcode)gcttcagtcctggtggctggttg	GTGACTGGAGTTCAGACGTGTGCTCTTCCGATCT(barcode)gcatgcaagctgcactccatgt
***EMX1* site4**	TCACTTACATAGATGTTTCC	TTGAT	ACACTCTTTCCCTACACGACGCTCTTCCGATCT(barcode)ggcttctttaggtaaccaaagcc	GTGACTGGAGTTCAGACGTGTGCTCTTCCGATCT(barcode)cagagatggctactggttggac
***EMX1* site5**	CAGTCCTCTAGCAAACTGAT	TTGAT	ACACTCTTTCCCTACACGACGCTCTTCCGATCT(barcode)gaaatatcggctggagccatagt	GTGACTGGAGTTCAGACGTGTGCTCTTCCGATCT(barcode)ggtaaattggattggccacttt
***EMX1* site6**	CAATGTGGAACTCACTGGAA	TTGAT	ACACTCTTTCCCTACACGACGCTCTTCCGATCT(barcode)ggtaccctttgcttcagcatccc	GTGACTGGAGTTCAGACGTGTGCTCTTCCGATCT(barcode)gagagagacgctgagagtgcga
***RNF2* site1**	CTCAAACTTTGAGGTTCATT	TTGAT	ACACTCTTTCCCTACACGACGCTCTTCCGATCT(barcode)gtcctgctggtcagaatcgttta	GTGACTGGAGTTCAGACGTGTGCTCTTCCGATCT(barcode)ggcacttcaaagaaacctgaca
***RNF2* site2**	CCACATACTCCTGTCATTAT	TTGAT	ACACTCTTTCCCTACACGACGCTCTTCCGATCT(barcode)gctctgctctaggaactctccag	GTGACTGGAGTTCAGACGTGTGCTCTTCCGATCT(barcode)gcagtgagctatgattgcctca
***RNF2* site3**	GAACATTTCTCTACCCAGGA	TTGAT	ACACTCTTTCCCTACACGACGCTCTTCCGATCT(barcode)ggccttcccagaaaagtagaact	GTGACTGGAGTTCAGACGTGTGCTCTTCCGATCT(barcode)gtccagggcctcatatgccaca

***RNF2* site4**	CCACAGAATATTACAGTTTA	TTGAT	ACACTCTTTCCCTACACGACGCTCTTCCGATCT(barcode)ggaaacaactggttgctggctga	GTGACTGGAGTTCAGACGTGTGCTCTTCCGATCT(barcode)gattcttgggtgaaccacaggg
***CLIC4* site1**	ACCTAAGAAGGAACCAGGAG	TTGAT	ACACTCTTTCCCTACACGACGCTCTTCCGATCT(barcode)ggaggtgaataggtgggttcgga	GTGACTGGAGTTCAGACGTGTGCTCTTCCGATCT(barcode)caggaaagtgtggggagaatga
***CLIC4* site2**	AGACCTTAACCTACTGCTAG	TTGAT	ACACTCTTTCCCTACACGACGCTCTTCCGATCT(barcode)gctccaggagagtagattccttg	GTGACTGGAGTTCAGACGTGTGCTCTTCCGATCT(barcode)gggcaacaactacccaccaaac
***CLIC4* site3**	GACATACATATATGTATATA	TTGAT	ACACTCTTTCCCTACACGACGCTCTTCCGATCT(barcode)ggaggagcagacagagttcaagt	GTGACTGGAGTTCAGACGTGTGCTCTTCCGATCT(barcode)gagctaccacatccagcctcat
***CLIC4* site4**	CTATCTAAAATAAAATGGAT	TTGAT	ACACTCTTTCCCTACACGACGCTCTTCCGATCT(barcode)ggcaggaggatcacctgaacctt	GTGACTGGAGTTCAGACGTGTGCTCTTCCGATCT(barcode)gaaccaccacatctggcccctа
***PCSK9* site1**	GTTGCAATACTGAGTCCTAA	TTGAT	ACACTCTTTCCCTACACGACGCTCTTCCGATCT(barcode)gagaagcagaaccagtaggatgt	GTGACTGGAGTTCAGACGTGTGCTCTTCCGATCT(barcode)ggagtaaggcaggttactctct
***PCSK9* site2**	CCCCAAAATTAACCATCACT	TTGAT	ACACTCTTTCCCTACACGACGCTCTTCCGATCT(barcode)ggcctgcccaagttatagagagt	GTGACTGGAGTTCAGACGTGTGCTCTTCCGATCT(barcode)ggaccagctggcttttccgaat
***VEGFA* site1**	TAGTCATCTTCTCCCCTATC	TTGAT	ACACTCTTTCCCTACACGACGCTCTTCCGATCT(barcode)cagttaccaaccccttgcccagg	GTGACTGGAGTTCAGACGTGTGCTCTTCCGATCT(barcode)gaccaccaatgggcacagaatc
***VEGFA* site2**	GGTGGAAAGCTTAGGGAAGT	TTGAT	ACACTCTTTCCCTACACGACGCTCTTCCGATCT(barcode)catcgaggttggcccaggattca	GTGACTGGAGTTCAGACGTGTGCTCTTCCGATCT(barcode)gctctgtaatgccactctttgg
***CCR5* site1**	TACCTAGTAGTCATTTCATG	TTGAT	ACACTCTTTCCCTACACGACGCTCTTCCGATCT(barcode)gatttccttcccatcccagctga	GTGACTGGAGTTCAGACGTGTGCTCTTCCGATCT(barcode)gctcagtgctatccctgaatga

***IFNG* site1**	GACCTTCTTTGCTCCAAAAC	TTGAT	ACACTCTTTCCCTACACGACGCTCTTCCGATCT(barcode)gctgagaagatgtgtgttctcct	GTGACTGGAGTTCAGACGTGTGCTCTTCCGATCT(barcode)gttggctagagacttgcagtgg
***AGBL1* site1**	TGTTGGCTCAAACACCAGAT	TTGAT	ACACTCTTTCCCTACACGACGCTCTTCCGATCT(barcode)gcctctgatactgacccatcagg	GTGACTGGAGTTCAGACGTGTGCTCTTCCGATCT(barcode)gttgcgagcgactgtatacata
**Cas9 target site**	**spacer sequence**	**PAM**	**PCR1 forward primer**	**PCR1 reverse primer**
***PD1* sg4**	CTTCCACATGAGCGTGGTCA	GGG	ACACTCTTTCCCTACACGACGCTCTTCCGATCT(barcode)agagcttcgtgctaaactggta	GTGACTGGAGTTCAGACGTGTGCTCTTCCGATCT(barcode)agaggtaggtgccgctgtcattg
***PPP1R12C* site 3**	GAGCTCACTGAACGCTGGCA	TGG	ACACTCTTTCCCTACACGACGCTCTTCCGATCT(barcode)cacttcgctcctgccaacct	GTGACTGGAGTTCAGACGTGTGCTCTTCCGATCT(barcode)atgggaatggtatggtgcgc
***PPP1R12C* site 14**	GGTCATACACTGGGCTGGCC	AGG	ACACTCTTTCCCTACACGACGCTCTTCCGATCT(barcode)gcaattcctcttctgcccgc	GTGACTGGAGTTCAGACGTGTGCTCTTCCGATCT(barcode)gggacacacgactgcatgga
***EGFR* sg43**	TCAGGGCATGAACTACTTGG	AGG	ACACTCTTTCCCTACACGACGCTCTTCCGATCT(barcode)ccagccataagtcctcgacg	GTGACTGGAGTTCAGACGTGTGCTCTTCCGATCT(barcode)gtgatcttgacatgctgcgg
***EGFR* sg50**	ATGCAGAAGGAGGCAAAGTA	AGG	ACACTCTTTCCCTACACGACGCTCTTCCGATCT(barcode)acctggcagccaggaacgta	GTGACTGGAGTTCAGACGTGTGCTCTTCCGATCT(barcode)ctgcgagctcacccagaatgtc
***CCR5* sg3**	GTAGAGCGGAGGCAGGAGGC	GGG	ACACTCTTTCCCTACACGACGCTCTTCCGATCT(barcode)gcacagggtggaacaagatggat	GTGACTGGAGTTCAGACGTGTGCTCTTCCGATCT(barcode)ggtcagagatggccaggttgag

***CCR5* sg5**	TTCAATGTAGACATCTATGT	AGG	ACACTCTTTCCCTACACGACGCTCTTCCGATCT(barcode)gagccaagctctccatctagtgg	GTGACTGGAGTTCAGACGTGTGCTCTTCCGATCT(barcode)gataatccatcttgttccaccc
***HBG* sg5**	CAAGGCTATTGGTCAAGGCA	AGG	ACACTCTTTCCCTACACGACGCTCTTCCGATCT(barcode)ctgctgaagggtgcttcctttta	GTGACTGGAGTTCAGACGTGTGCTCTTCCGATCT(barcode)ccttccccacactatctcaatg
**ABE site 20**	TTAAGCTGTAGTATTATGAA	GGG	ACACTCTTTCCCTACACGACGCTCTTCCGATCT(barcode)aagtgttcagctgcttttctttc	GTGACTGGAGTTCAGACGTGTGCTCTTCCGATCT(barcode)cctagcctccatgtcctatttc
**ABE site 27**	CGGGCATCAGAATTCCCTGG	AGG	ACACTCTTTCCCTACACGACGCTCTTCCGATCT(barcode)cactccctccttggtccaggttc	GTGACTGGAGTTCAGACGTGTGCTCTTCCGATCT(barcode)tgtccaagaagcaacagttctt
***CHM13* sg1**	GTCAAGAAAGCAGAGACTGC	CGG	ACACTCTTTCCCTACACGACGCTCTTCCGATCT(barcode)gtggcaggcagattatcattccc	GTGACTGGAGTTCAGACGTGTGCTCTTCCGATCT(barcode)tcttgggggtttcctgtcttcc
***EMX1* sg2p**	GACATCGATGTCCTCCCCAT	TGG	ACACTCTTTCCCTACACGACGCTCTTCCGATCT(barcode)ggcccaggtgaaggtgtggttcc	GTGACTGGAGTTCAGACGTGTGCTCTTCCGATCT(barcode)ctgccctcgtgggtttgtggtt
***RUNX1* sg1**	GAAGAGGGTGCATTTTCAGG	AGG	ACACTCTTTCCCTACACGACGCTCTTCCGATCT(barcode)agagatgtagggctagaggggtg	GTGACTGGAGTTCAGACGTGTGCTCTTCCGATCT(barcode)cacctctcatgaagcactgtgg
***DNMT1* sg1**	GGAGTGAGGGAAACGGCCCC	AGG	ACACTCTTTCCCTACACGACGCTCTTCCGATCT(barcode)cgtggccccatctttctcaagg	GTGACTGGAGTTCAGACGTGTGCTCTTCCGATCT(barcode)cgttcacggagactgaacactc
***FANCF* site 1**	GGAATCCCTTCTGCAGCACC	TGG	ACACTCTTTCCCTACACGACGCTCTTCCGATCT(barcode)gcgccgatggatgtggcgca	GTGACTGGAGTTCAGACGTGTGCTCTTCCGATCT(barcode)ggcgtatcatttcgcggatg
**ABE site 5**	GATGAGATAATGATGAGTCA	GGG	ACACTCTTTCCCTACACGACGCTCTTCCGATCT(barcode)aacctagaccgctgaacttttcc	GTGACTGGAGTTCAGACGTGTGCTCTTCCGATCT(barcode)gcgcctggtcacattgacttta

**ABE site 8**	GTAAACAAAGCATAGACTGA	GGG	ACACTCTTTCCCTACACGACGCTCTTCCGATCT(barcode)ctgccgtgggagacaattcatat	GTGACTGGAGTTCAGACGTGTGCTCTTCCGATCT(barcode)gttctgtttgtggccaagaatt
**ABE site 10**	GAACATAAAGAATAGAATGA	TGG	ACACTCTTTCCCTACACGACGCTCTTCCGATCT(barcode)ggtacatatacacaatggagtca	GTGACTGGAGTTCAGACGTGTGCTCTTCCGATCT(barcode)catccccacattatcctccacc
**HEK site 7**	GGAACACAAAGCATAGACTG	CGG	ACACTCTTTCCCTACACGACGCTCTTCCGATCT(barcode)ggcaggacgtctgcccaata	GTGACTGGAGTTCAGACGTGTGCTCTTCCGATCT(barcode)attgtccagccccatctgtc
***FANCF* sg5**	GGACTCTCTGATGAAGACCC	AGG	ACACTCTTTCCCTACACGACGCTCTTCCGATCT(barcode)ttaccacctggtgcagcaactct	GTGACTGGAGTTCAGACGTGTGCTCTTCCGATCT(barcode)caggctgctgagaaacctggcg
***FGF6* sg4**	ATAAGCCCCTGCCGCCATGC	CGG	ACACTCTTTCCCTACACGACGCTCTTCCGATCT(barcode)atatcatcaactagggacggagc	GTGACTGGAGTTCAGACGTGTGCTCTTCCGATCT(barcode)gggccatccaccttgcctctca
***HBG* sg10**	GCTAAACTCCACCCATGGGT	TGG	ACACTCTTTCCCTACACGACGCTCTTCCGATCT(barcode)agcagtatcctcttgggggcccc	GTGACTGGAGTTCAGACGTGTGCTCTTCCGATCT(barcode)cctcagacgttccagaagcgag
**HEK site 2**	GAACACAAAGCATAGACTGC	GGG	ACACTCTTTCCCTACACGACGCTCTTCCGATCT(barcode)acaatgataacaagacctggctg	GTGACTGGAGTTCAGACGTGTGCTCTTCCGATCT(barcode)agtgagaagccagtggaataca
**ABE site11**	GGACAGGCAGCATAGACTGT	GGG	ACACTCTTTCCCTACACGACGCTCTTCCGATCT(barcode)ccctaaaccacctgcagaggac	GTGACTGGAGTTCAGACGTGTGCTCTTCCGATCT(barcode)agacagcaattttgccaaagac
**ABE site 4**	GAGCAAAGAGAATAGACTGT	AGG	ACACTCTTTCCCTACACGACGCTCTTCCGATCT(barcode)ccttaagtgttcagctgcttttc	GTGACTGGAGTTCAGACGTGTGCTCTTCCGATCT(barcode)cactgcacctagcctccatgtc
**ABE site 14**	GGCTAAAGACCATAGACTGT	GGG	ACACTCTTTCCCTACACGACGCTCTTCCGATCT(barcode)gcctctcatttagaacctgaagc	GTGACTGGAGTTCAGACGTGTGCTCTTCCGATCT(barcode)caccgtgcccggccaaagat

**ABE site 16**	GGGAATAAATCATAGAATCC	TGG	ACACTCTTTCCCTACACGACGCTCTTCCGATCT(barcode)tccagcaacacgcggggagg	GTGACTGGAGTTCAGACGTGTGCTCTTCCGATCT(barcode)ttctgaccccatgcaccctc
***CCR5* sg1p**	TAATAATTGATGTCATAGAT	TGG	ACACTCTTTCCCTACACGACGCTCTTCCGATCT(barcode)aacaccagtgagtagagcgg	GTGACTGGAGTTCAGACGTGTGCTCTTCCGATCT(barcode)atgtagacatctatgtaggc
