# Supplementary material for: Zero-shot prediction of mutation effects with multimodal deep representation learning guides protein engineering
Source: Cell Res. 2024 Jul 5;34(9):630–47. doi: 10.1038/s41422-024-00989-2 (PMC11369238; doi:10.1038/s41422-024-00989-2)
Supplement: Supplementary file 27 — Supplementary information, Data S6 [file 41422_2024_989_MOESM27_ESM.pdf]

Data S6 | The list of off-target sites and primers.

site name	spacer sequence	PAM	PCR1 forward primer	PCR1 reverse primer
***AGBL1* On-target**	TGTTGGCTCAAACACCAGAT	TTGAT	ACACTCTTTCCCTACACGACGCTCTTCCGATCT(barcode)ctctccacgcagatttctcttct	GTGACTGGAGTTCAGACGTGTGCTCTTCCGATCT(barcode)ctctccacgcagatttctcttct
***AGBL1* OFF-1**	TtTTGGCcCcAACACCAGga	TTGAT	ACACTCTTTCCCTACACGACGCTCTTCCGATCT(barcode)ggactctaaagcattccacacaa	GTGACTGGAGTTCAGACGTGTGCTCTTCCGATCT(barcode)ggaaaggggcaacaaagaggga
***AGBL1* OFF-2**	TcTTGGCTCAAAaAaCAGAg	TTGAT	ACACTCTTTCCCTACACGACGCTCTTCCGATCT(barcode)ccttagggctgcccaggaactaa	GTGACTGGAGTTCAGACGTGTGCTCTTCCGATCT(barcode)ccttgaacagccacctaaaaag
***AGBL1* OFF-3**	TGTTGGCTCtgcCACCtGcT	TTGAT	ACACTCTTTCCCTACACGACGCTCTTCCGATCT(barcode)ccaggcccttccctaaactctgc	GTGACTGGAGTTCAGACGTGTGCTCTTCCGATCT(barcode)gctcggcctaggaagaagtgcc
***AGBL1* OFF-4**	ccTTGGCTCAAACACttGgT	TTGAT	ACACTCTTTCCCTACACGACGCTCTTCCGATCT(barcode)caggctaggccagtcctagagg	GTGACTGGAGTTCAGACGTGTGCTCTTCCGATCT(barcode)aggaaaactgaggcttagcaac
***AGBL1* OFF-5**	GcaGGCTCAAAggCCAaAT	TTGAT	ACACTCTTTCCCTACACGACGCTCTTCCGATCT(barcode)gcaacattcagttcactgccaag	GTGACTGGAGTTCAGACGTGTGCTCTTCCGATCT(barcode)gaagccctagacataggagggt
***HBG* Site5 On-target**	GTGGGGAAGGGGCCCCCAAG	AGG	ACACTCTTTCCCTACACGACGCTCTTCCGATCT(barcode)atgggtggagtttagccagggac	GTGACTGGAGTTCAGACGTGTGCTCTTCCGATCT(barcode)tggtgggagaagaaaactagct
***HBG* Site5 OFF-1**	GgtGGGAtGGGGtCCCCAAG	TGG	ACACTCTTTCCCTACACGACGCTCTTCCGATCT(barcode)ggaatttggccctaaacctggag	GTGACTGGAGTTCAGACGTGTGCTCTTCCGATCT(barcode)gtcagtgaagacacaggaattg
***HBG* Site5 OFF-2**	GgtaGGgAGaGGCCCCCAga	GGG	ACACTCTTTCCCTACACGACGCTCTTCCGATCT(barcode)gttcctctcatctgctctcatct	GTGACTGGAGTTCAGACGTGTGCTCTTCCGATCT(barcode)ggtagaaagaggactccaggag

***HBG* Site5 OFF-3**	GgtGGGgAGcGGCCCCCcAG	TGG	ACACTCTTTCCCTACACGACGCTCTTCCGATCT(barcode)ggtagggagcagaaagtgtagcg	GTGACTGGAGTTCAGACGTGTGCTCTTCCGATCT(barcode)catgcctggccagaatcgagca
***DNMT1* sg1 On-target**	GGAGTGAGGGAAACGGCCCC	AGG	ACACTCTTTCCCTACACGACGCTCTTCCGATCT(barcode)cgtggccccatctttctcaagg	GTGACTGGAGTTCAGACGTGTGCTCTTCCGATCT(barcode)cgttcacggagactgaacactc
***DNMT1* sg1 OFF-1**	GGAGaGAGGGtAAtGGCCCC	AGG	ACACTCTTTCCCTACACGACGCTCTTCCGATCT(barcode)atcagacggcttccttccccgc	GTGACTGGAGTTCAGACGTGTGCTCTTCCGATCT(barcode)ctctgggacatttccataagct
***DNMT1* sg1 OFF-2**	GGAGTGAGGGAAAgGGCtCt	GGG	ACACTCTTTCCCTACACGACGCTCTTCCGATCT(barcode)gctatgaaggaaacaatgagcg	GTGACTGGAGTTCAGACGTGTGCTCTTCCGATCT(barcode)tgcctgcccatcttgtgcccac
***DNMT1* sg1 OFF-3**	GGAGaGAGGGAAACGGtCtC	AGG	ACACTCTTTCCCTACACGACGCTCTTCCGATCT(barcode)gggaggcacgaagacaggaggg	GTGACTGGAGTTCAGACGTGTGCTCTTCCGATCT(barcode)taaggggagccagcgacctcta
***EMX1* sg2p On-target**	GACATCGATGTCCTCCCCAT	TGG	ACACTCTTTCCCTACACGACGCTCTTCCGATCT(barcode)ggcccaggtgaaggtgtggttcc	GTGACTGGAGTTCAGACGTGTGCTCTTCCGATCT(barcode)ctgccctcgtgggtttgtggtt
***EMX1* sg2p OFF-1**	GAaATCaAgGTCCTCCCCAT	AGG	ACACTCTTTCCCTACACGACGCTCTTCCGATCT(barcode)ggctggtacacccatctctcatc	GTGACTGGAGTTCAGACGTGTGCTCTTCCGATCT(barcode)gatgtgatatgagctgaagggc
***EMX1* sg2p OFF-2**	GACATCGATagCCTCCCCAc	TGG	ACACTCTTTCCCTACACGACGCTCTTCCGATCT(barcode)gcatgtcaggatctcaactctt	GTGACTGGAGTTCAGACGTGTGCTCTTCCGATCT(barcode)agcaacaggaagccagagtagc
***EMX1* sg2p OFF-3**	cACATaGgTGTCCTCCCCAT	AGG	ACACTCTTTCCCTACACGACGCTCTTCCGATCT(barcode)gctcgtcagaacggtgccctcc	GTGACTGGAGTTCAGACGTGTGCTCTTCCGATCT(barcode)tatgtacatttctgggctcttc
**R-loop 1**	GTGGTAGACAGCATGTGTCCTA	AAGGGT	ACACTCTTTCCCTACACGACGCTCTTCCGATCT(barcode)tggtggagtgctctgtgtttg	GTGACTGGAGTTCAGACGTGTGCTCTTCCGATCT(barcode)tgcagtctcctgcttctctg

**R-loop 2**	ATTTACAGCCTGGCCTTTGGGG	TCGGGT	ACACTCTTTCCCTACACGACGCTCTTCCGATCT(barcode)ctacagaaaggtcagcagcta	GTGACTGGAGTTCAGACGTGTGCTCTTCCGATCT(barcode)ggacatttccaccgcaaaatg
